# Clinical Outcomes of Cervical Adenocarcinoma In Situ According to Conservative or Demolitive Treatment: A Systematic Review and Meta-Analysis

**DOI:** 10.3390/cancers17111839

**Published:** 2025-05-30

**Authors:** Giovanni Delli Carpini, Camilla Cicoli, Marco Bernardi, Jacopo Di Giuseppe, Luca Giannella, Andrea Ciavattini

**Affiliations:** Gynecologic Section, Department of Odontostomatologic and Specialized Clinical Sciences, Università Politecnica delle Marche, 60123 Ancona, Italy; g.dellicarpini@staff.univpm.it (G.D.C.); camilla.cicoli@ospedaliriuniti.marche.it (C.C.); marco.bernardi@ospedaliriuniti.marche.it (M.B.); jacopo.digiuseppe@ospedaliriuniti.marche.it (J.D.G.); l.giannella@staff.univpm.it (L.G.)

**Keywords:** adenocarcinoma in situ, conization, conservative treatment, hysterectomy, fertility sparing, recurrence, neoplasm residual

## Abstract

Background/Objectives: The objective of this systematic review and meta-analysis was to compare the risk of recurrence and residual disease between conservative and demolitive treatment in patients diagnosed with cervical adenocarcinoma in situ (AIS). Methods: Bibliographic databases (CENTRAL, PubMed, Cochrane Database of Systematic Reviews, and Google Scholar) were searched for studies published up to December 2024 reporting both conservative and demolitive treatment outcomes. Exclusion criteria were the lack of outcomes of interest, the reporting of only one treatment modality, and a lack of follow-up. The ROBINS-I tool was used to assess the risk of bias. The evaluated outcomes were AIS or invasive recurrence and AIS or invasive residual after margin positivity at the first excision. A meta-analysis with a fixed-effect model and the Mantel–Haenszel method for risk ratio (RR) with a 95% confidence interval (CI) was performed. Heterogeneity was evaluated with the chi-squared test and quantified with the I2 method. A meta-regression was performed using the year of publication, CKC use, margin positivity, and follow-up duration as predictors. Results: Nineteen studies were included, reporting data about 5934 patients with AIS diagnosis after conization. The meta-analysis showed a higher risk of recurrence as AIS in conservatively treated patients (11 studies, RR = 8.44, 95% CI 3.36–21.19, *p* < 0.001, I2 = 0.0%, *p* = 0.73), while no differences were observed in the risk of recurrence as invasive adenocarcinoma (10 studies, RR = 1.67, 95% CI 0.82–3.39, *p* = 0.16, I2 = 0.00%, *p* = 0.48). No difference between the two treatment modalities in terms of AIS residual (nine studies, RR = 0.89, 95% CI 0.62–1.26, *p* = 0.50, I2 = 33%, *p* = 0.15) or invasive residual (three studies, RR = 0.48, 95% CI 0.09–2.41, *p* = 0.37, I2 = 0.0%, *p* = 0.94) after margin positivity at the first excision emerged. The meta-regression showed no association with the predictors. Conclusions: The safety profile of a conservative treatment for AIS patients seems not to differ from that of a demolitive approach in terms of invasive recurrence or residual, while it seems to be associated with a higher risk of AIS recurrence. Future research should focus on optimizing follow-up strategies to detect AIS recurrences early.

## Simple Summary

The standard treatment with hysterectomy for cervical adenocarcinoma in situ (AIS) may not often be indicated, given the high incidence of AIS before the completion of reproductive desire. We aimed to evaluate the safety of the conservative approach in terms of recurrence after treatment and residual in the case of re-excision for positive margins with respect to hysterectomy. This systematic review and meta-analysis showed that patients subjected to conservative treatment seem not to be at higher risk of invasive disease in terms of recurrence or residual. In comparison, they seem to present a higher risk of AIS recurrence. Therefore, the conservative approach seems to be safe, even if particular attention should be given to the identification of AIS recurrence during follow-up.

## 1. Introduction

Cervical cancer is the second most common gynecological malignancy and the second leading cause of cancer death in patients of reproductive age worldwide [1]. Human papillomavirus (HPV) is responsible for nearly 100% of squamous cell cervical carcinomas and for about 85% of adenocarcinomas [2]. Adenocarcinomas account for about 15–25% of all cervical cancers [3]. Adenocarcinoma in situ (AIS) is a glandular intraepithelial lesion arising from the glandular epithelium of the endocervix that is considered the precursor of adenocarcinoma [4]. While the incidence of squamous cell carcinoma has decreased over the past two decades as a result of the adoption of screening programs, the incidence of invasive adenocarcinoma and AIS is increasing, particularly in patients in their 30s and 40s (11.2 per 100,000 persons [5]), when the reproductive desire is still strong [6,7,8,9]. Early diagnosis and treatment of AIS are challenging due to the extension into the glandular crypts within the cervical canal, multifocality with skip lesions, and unspecific colposcopic features. Thus, the diagnostic performance of conventional screening methods and colposcopy is limited [10]. Late diagnosis is common, with an 8% risk of concomitant invasive adenocarcinoma [11]. 

A total hysterectomy is recommended for AIS by most guidelines, also due to the low reliability of the negative margin regarding the complete removal of the lesion [12,13,14,15]. Nevertheless, since more than 68% of AIS cases occur in patients under the age of 35 who have not completed their reproductive desire, hysterectomy may not often be acceptable. For this reason, conservative treatment with different techniques (electrosurgery, laser, or cold knife) has been proposed [15,16]. However, patients subjected to conservative treatment should not be at higher risk of residual disease, undiagnosed concomitant invasive lesions (particularly in the case of positive margin of the first excision [11]), or invasive recurrence during follow-up. Therefore, there is a need for reliable data about the safety of the conservative approach compared to the hysterectomy. In this context, a systematic review with a meta-analysis of studies reporting both conservative and demolitive treatment safety outcomes may be of clinical utility for the counseling, treatment choice, and follow-up of AIS patients. The objective of this systematic review with a meta-analysis was to compare the risk of recurrence during follow-up and the risk of residual disease in the case of margin positivity at the first excision for AIS between conservative and demolitive treatment.

## 2. Materials and Methods

This review was conducted according to Preferred Reporting Items for Systematic Reviews and Meta-Analyses (PRISMA) guidelines (Supplementary File S1) [17] and Meta-analyses Of Observational Studies in Epidemiology (MOOSE) guidelines (Supplementary File S2) [18]. This review was registered with the International Prospective Register of Systematic Reviews (PROSPERO, registration number 2024 CRD42024499302) on 15 January 2024. The search was performed without language or date restrictions. The inclusion criteria were as follows: (1) reports available in full text and (2) reporting data about the outcome of AIS for both conservative and demolitive approaches, without a follow-up limit. The exclusion criteria were as follows: (1) studies lacking outcomes of interest; (2) reporting about only one treatment modality; (3) studies lacking follow-up data; (4) studies including patients with invasive adenocarcinoma or squamous carcinoma; (5) cross-sectional studies, case reports, narrative reviews, editorials, or letters to the editor. Unpublished manuscripts and conference abstracts were not included. Bibliographic databases (CENTRAL, PubMed, Cochrane Database of Systematic Reviews, and Google Scholar) were searched for studies published up to December 2024 on 20 January 2025. A gray literature search was also performed. Reference lists of eligible studies were searched for additional studies to be included. The search was re-run on 01 April 2025. 

The main domains of the search strategy were “Adenocarcinoma in Situ”, “Conization”, “Hysterectomy”, “Fertility-sparing”, “Recurrence”, and “Residual”. 

The complete search strategy was as follows: ((“Adenocarcinoma in Situ” [Mesh] OR “adenocarcinoma in situ” [tw] OR “high-grade cervical glandular intraepithelial neoplasia” [tw] OR “Adenocarcinoma in situ of the Uterine Cervix” [tw] OR “Adenocarcinoma of the Uterine Cervix” [tw] NOT lung [tw]) AND (“Conization” [Mesh] OR “Conservative Treatment” [Mesh] OR “Electrosurgery” [Mesh] OR “Lasers” [Mesh] OR “Hysterectomy” [Mesh] OR “Hysterectomy, Vaginal” [Mesh] OR “treatment *” [tw] OR “management” [tw] OR “conservative treatment *” [tw] OR “conservatively treated” [tw] OR conization [tw] OR conization [tw] OR “cervical excision *” [tw] OR “excision *” [tw] OR “loop electrosurgical excision procedure *” [tw] OR LEEP [tw] OR “large loop excision of the transformation zone” [tw] OR LLETZ [tw] OR “cold knife conization *” [tw] OR “cold-knife conization *” [tw] OR “laser conization *” [tw] OR “fertility sparing” [tw] OR fertility-sparing [tw] OR “excisional treatment *” [tw] OR “excision procedur *” [tw] OR “demolitive treatment” [tw] OR hysterectomy [tw])) AND (“Recurrence” [Mesh] OR “Neoplasm Recurrence, Local” [Mesh] OR “Treatment Outcome” [Mesh] OR “Neoplasm, Residual” [Mesh] OR residual * [tw] OR “occult disease *” [tw] OR “residual lesion *” [tw] OR recur * [tw] OR “outcome *” [tw] OR “persist *” [tw] OR “treatment failure *” [tw] OR “persistent disease *” [tw] OR “invasive recurrence *” [tw] OR “disease progression” [tw] OR “progression” [tw] OR “progressive disease” [tw]).

Two authors (G.D.C. and C.C.) independently screened the articles obtained from the initial search by reviewing the titles and abstracts. The articles obtained from the first selection were thoroughly reviewed. Two authors (J.D.G. and M.B.) independently extracted the data from each included study. Data were retrieved from the manuscripts where available. If not, the authors were contacted by email and asked for the original data, with a reminder sent after 15 days in the case of no response. Disagreements were solved through discussion; in the case of failure of discussion, a different author (A.C.) made the final judgment. 

The population was composed of patients who had a histopathological diagnosis of AIS after cervical conization and were subsequently subjected to conservative or demolitive treatment with the availability of follow-up data. Patients were excluded in the case of concomitant squamous carcinoma or invasive adenocarcinoma at diagnosis. The intervention of interest was the “conservative treatment”, defined as the management of patients diagnosed with AIS after conization (electrosurgery, laser, or cold knife [15,16]) without a hysterectomy being performed. Patients subjected to re-conization for a positive margin at the first conization were considered conservatively managed. The comparator was the “demolitive treatment”, defined as laparoscopic, laparotomic, vaginal, or robotic total hysterectomy with or without salpingo-oophorectomy. The primary outcomes were AIS or invasive recurrence, defined as any histopathological diagnosis during follow-up of AIS or invasive disease, respectively, and AIS or invasive residual disease after positive margin, defined as any histopathological evidence of AIS or invasive disease at the second excision performed in the case of margin positivity at the first conization for conservative treatment or at the hysterectomy performed after margin positivity at the first conization for demolitive treatment. All the identified outcomes were dichotomic. The following data items were extracted: year of publication, country, study design, study period, age, parity, menopause, oral contraceptive use, smoking, HPV positivity, abnormal cytology, colposcopic impression, histology at biopsy, surgical techniques, cone length, co-existence with squamous disease, endocervical curettage (ECC) performed, abnormal ECC, margin positivity, second conization, rate of complications, pregnancy rate, follow-up modality, follow-up duration, treatment modality for recurrence, and number of included patients. The ROBINS-I tool (updated on 20 October 2016) was used for risk of bias assessment [19]. Two authors (G.D.C. and L.G.) performed the assessment independently. Disagreements were solved through discussion; if the discussion failed, a different author (A.C.) made the final judgment. 

The measure of effect was the risk ratio (RR) with a 95% confidence interval (CI) of the risk of recurrence of conservative treatment compared to demolitive treatment and of the risk of residual disease at the second excision performed after margin positivity at the first excision compared to hysterectomy. 

The baseline elements of the included studies were qualitatively synthesized. Data regarding the outcome of interest were collected as absolute numbers; when data were expressed as percentages, they were converted into absolute numbers. Studies were grouped for synthesis according to the reported outcomes (recurrence and/or residual). R software (version 4.4.1) with the “metafor” package [20] and RevMan software (version 9.0.0) were used. A meta-analysis with a fixed-effect model and the Mantel–Haenszel method [21] was performed for the RR with 95% CI determination for the study outcomes, comparing conservative and demolitive approaches. Studies with zero events in both arms of treatment were excluded from the meta-analysis [21]. Statistical heterogeneity between included studies was evaluated with the chi-squared test and quantified with the I2 method. A visual display for data presentation was included (forest plot). A funnel plot was generated to evaluate small-study effects, with the risk ratio as the effect estimate and the standard error as the measure of precision. The Egger test was used to evaluate funnel plot asymmetry using weighted regression with the multiplicative dispersion method. A meta-regression was performed using four continuous predictors: year of publication, rate of CKC use per study, rate of margin positivity per study (only for AIS recurrence and invasive recurrence), and follow-up duration per study. Each predictor was tested individually due to the limited number of included studies to avoid overfitting. The results were reported as regression coefficients (log-relative risks) with 95% confidence intervals. A *p* value < 0.05 was considered as statistically significative. The Grades of Recommendation, Assessment, Development and Evaluation (GRADE) approach was used for certainty of evidence assessment, with the following domains: risk of bias, inconsistency, indirectness, imprecision, and publication bias [22]. An overall judgment of the level of certainty was defined according to the GRADE indications [22], using GRADEpro GDT software (https://www.gradepro.org) [23]. Two authors (G.D.C. and C.C.) performed the assessment independently. Disagreements were solved through discussion; if the discussion failed, a different author (A.C.) made the final judgment. A finding summary table was used to report the results of the assessment of certainty.

## 3. Results

### 3.1. Study Selection, Study Characteristics, and Risk of Bias of Included Studies

**Figure 1:**
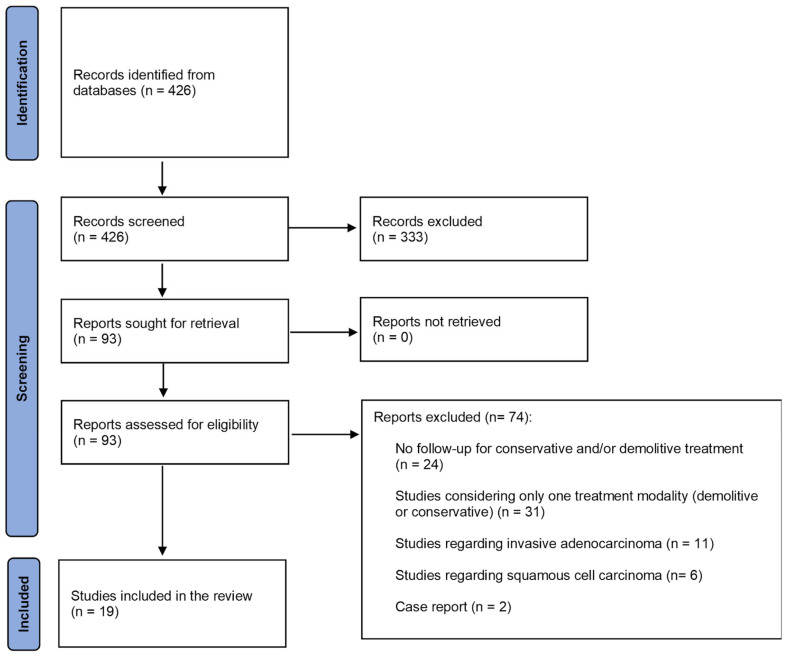
Flow diagram of study selection.

Nineteen studies were included [24,25,26,27,28,29,30,31,32,33,34,35,36,37,38,39,40,41,42], reporting data about 5934 patients diagnosed with AIS after conization. Among them, 4621 (77.9%) were subjected to conservative treatment, and 1313 (22.1%) to demolitive treatment. Figure 1 reports the flow diagram of the study selection process. Table 1 summarizes the characteristics of each included study. Table 2 reports the intervention details for each study. The complete risk of bias evaluation is available as Supplementary File S3.

### 3.2. Patient Characteristics

The mean reported age ranged from 32.1 to 44.2 years (19 studies) [24,25,26,27,28,29,30,31,32,33,34,35,36,37,38,39,40,41,42]. Among the seven studies [30,32,33,34,35,36,42] reporting data for conservatively treated patients and patients subjected to hysterectomy, the mean age ranged from 29 to 37.6 years and 40 to 46 years, respectively. The HPV positivity rate at diagnosis ranged from 66.2% to 97.9% (six studies) [25,29,32,33,37,42]. Margin positivity ranged from 17.1% to 57.5% (19 studies) [24,25,26,27,28,29,30,31,32,33,34,35,36,37,38,39,40,41,42]. Three studies reported data about complications of conservative treatment: 1.2–9.5% bleeding (three studies) [26,27,38], 3.6% pelvic pain (one study) [26], 3.6% cervical stenosis (one study) [26], 9.5% infection (one study) [38], and 4.8% bladder perforation (one study) [38]. The pregnancy rate was reported in five studies and ranged from 10.8% to 28.3% [25,27,36,40,42]. No case of preterm birth was reported (two studies) [28,40]. Follow-up duration varied widely between the 19 included studies (5.6–117.6 months) [24,25,26,27,28,29,30,31,32,33,34,35,36,37,38,39,40,41,42]. Six studies reported that all patients subjected to conservative treatment with AIS or invasive recurrence underwent hysterectomy [24,25,32,36,37,42]. One study reported a new conservative treatment in the case of AIS recurrence [33]. In the case of recurrence after demolitive treatment, one study reported electrocautery or excision of vaginal disease, and one study reported the use of chemoradiotherapy [33,42]. The synthesis of the remaining characteristics is reported in Supplementary Files S4 and S5.

### 3.3. Synthesis of Results

#### 3.3.1. AIS Recurrence (16 Studies)

**Figure 2:**
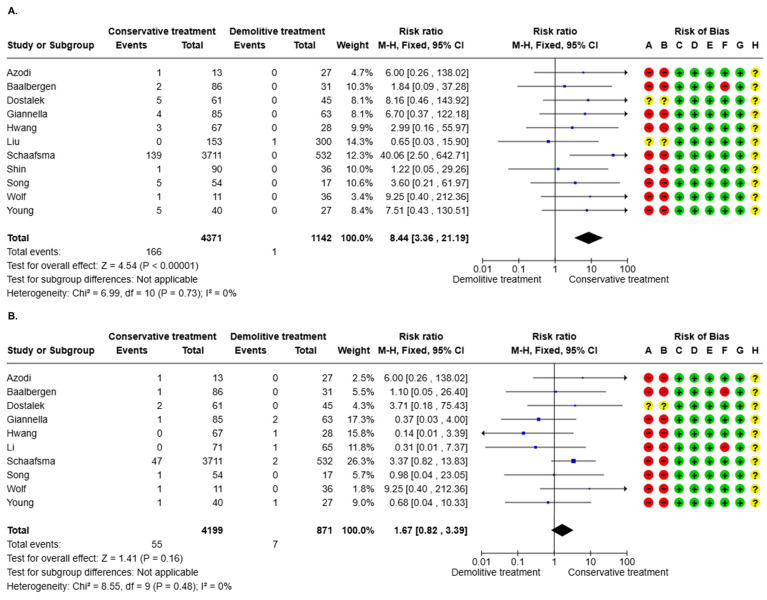
(**A**) Adenocarcinoma in situ (AIS) recurrence according to treatment modality. (**B**) Invasive recurrence according to treatment modality.

Five studies were excluded from the meta-analysis since they had zero events in both arms of treatment. Conservative treatment was associated with an increased risk of AIS recurrence (RR = 8.44, 95% CI 3.36–21.19, *p* < 0.001). The statistical heterogeneity was low (I2 = 0.0%, *p* = 0.73) (Figure 2A). Two (18.2%) studies presented a “moderate” overall risk of bias, while 9/11 (81.8%) had a “serious” overall risk of bias. The Egger test results were b = 16.15, 95% CI 3.44–28.85, *p* = 0.03 (Supplementary File S6). The overall level of certainty was “very low” (Table 3).

#### 3.3.2. Invasive Recurrence (16 Studies)

Six studies were excluded from the meta-analysis since they had zero events in both arms of treatment. No difference in the risk of invasive recurrence between conservative and demolitive treatment was evidenced (RR = 1.67, 95% CI 0.82–3.39, *p* = 0.16). The statistical heterogeneity was low (I2 = 0.0%, *p* = 0.48) (Figure 2B). One (10.0%) study presented a “moderate” overall risk of bias, while 9/10 (90.0%) had a “serious” overall risk of bias. The Egger test results were b = 1.76, 95% CI −1.33–4.85, *p* = 0.32 (Supplementary File S6). The overall level of certainty was “very low” (Table 3).

#### 3.3.3. AIS Residual After Positive Margin (10 Studies)

**Figure 3:**
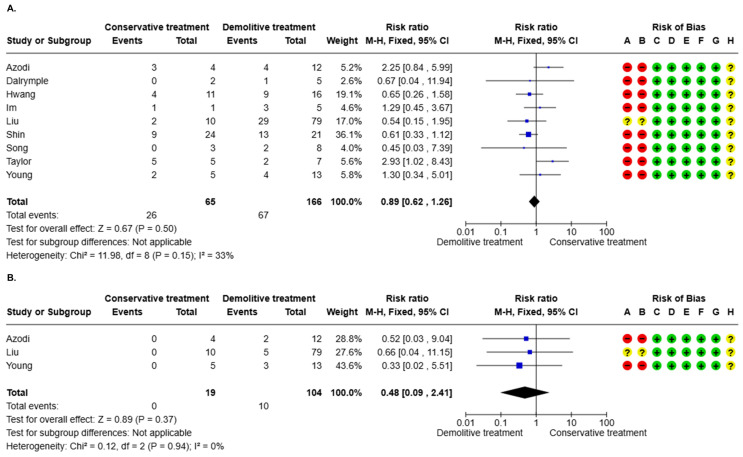
(**A**) AIS residual according to treatment modality. (**B**) Invasive residual according to treatment modality.

One study was excluded from the meta-analysis since it had zero events in both arms of treatment. No difference in the risk of AIS residual after a positive margin between conservative and demolitive treatment was evidenced (RR = 0.89, 95% CI 0.62–1.26, *p* = 0.50). The statistical heterogeneity was moderate (I2 = 33%, *p* = 0.15) (Figure 3A). One (11.1%) study presented a “moderate” overall risk of bias, while 8/9 (88.9%) had a “serious” overall risk of bias. The Egger test results were b = −0.23, 95% CI −1.62–1.17, *p* = 0.72 (Supplementary File S6). The overall level of certainty was “very low” (Table 3).

#### 3.3.4. Invasive Residual After Positive Margin (10 Studies)

Seven studies were excluded from the meta-analysis since they had zero events in both arms of treatment. No difference in the risk of invasive residual after a positive margin between conservative and demolitive treatment was evidenced (RR = 0.48, 95% CI 0.09–2.41, *p* = 0.37). The statistical heterogeneity was low (I2 = 0.0%, *p* = 0.94) (Figure 3B). One (33.3%) study presented a “moderate” overall risk of bias, while the remaining 2/3 (66.7%) had a “serious” overall risk of bias. The Egger test results were b = −22.32, 95% CI −426.42–381.79, *p* = 0.62 (Supplementary File S6). The overall level of certainty was “very low” (Table 3).

### 3.4. Meta-Regression

Meta-regression showed no association of the predictors with AIS recurrence (year of publication: coefficient 0.0224, 95% CI −0.0670 to 0.1118, *p* = 0.6234; CKC use: coefficient −0.0224, 95% CI −0.0323 to 0.0275, *p* = 0.8754; margin positivity: coefficient 0.0416, 95% CI −0.0355 to 0.1187, *p* = 0.2900; follow-up duration: coefficient 0.0257, 95% CI −0.0114 to 0.0629, *p* = 0.1741). No association was also found with invasive recurrence (year of publication: coefficient 0.0004, 95% CI −0.0865 to 0.0873, *p* = 0.9932; CKC use: coefficient −0.0184, 95% CI −0.0099 to 0.0468, *p* = 0.2027; margin positivity: coefficient 0.0562, 95% CI −0.0420 to 0.1544, *p* = 0.2618; follow-up duration: coefficient 0.0131, 95% CI −0.0085 to 0.0348, *p* = 0.2345). AIS residual was not associated with the year of publication (coefficient −0.0045, 95% CI −0.0632 to 0.0541, *p* = 0.8799), CKC use (coefficient 0.0099, 95% CI −0.0119 to 0.0317, *p* = 0.8910), or follow-up duration (coefficient −0.0150, 95% CI −0.0448 to 0.0148, *p* = 0.3233). No association emerged with invasive residual (year of publication: coefficient 0.0152, 95% CI −0.1566 to 0.1870, *p* = 0.1733; CKC use: coefficient −0.0365, 95% CI −0.3646 to 0.2916, *p* = 0.8274; follow-up duration: coefficient 0.0188, 95% CI −0.0944 to 0.1320, *p* = 0.7452).

## 4. Discussion

### 4.1. Summary of Main Results

The conservative approach for AIS diagnosed after conization seems to be associated with a higher risk of recurrence as an in situ lesion than the demolitive approach. There seems to be no difference in the risk of recurrence as an invasive lesion. In the case of margin positivity at the first conization, there seems to be no difference in the risk of residual disease between repeat conization and hysterectomy. The overall certainty of the evidence was very low. The year of publication, CKC use, margin positivity, or follow-up duration seemed to have no impact on the risk of recurrence or residual.

### 4.2. Comparison with Existing Literature

To date, most guidelines recommend hysterectomy as the standard treatment for AIS [12,13,14,15]. This choice is indicated mainly due to the nonspecific colposcopic features of AIS and to the lower reliability of the negative margin regarding the complete removal of the lesion [15]. However, the diagnosis of AIS in patients with reproductive desire requires evaluating conservative management to preserve fertility [6,7,8,9]. Reliable data about its safety should support this choice compared to the standard treatment with hysterectomy.

To begin with, patients subjected to a conservative approach should not be at higher risk of undiagnosed invasive residual. This condition is crucial since it is reported that the risk of concomitant invasive disease at the first conization after an AIS diagnosis at cervical biopsy is about 7.8% [43], and the risk of adenocarcinoma at the re-excision or hysterectomy after margin positivity was reported to be 5.9% [11]. The results of this paper demonstrate how the risk of invasive disease at re-excision or hysterectomy in the case of margin positivity at the first conization for AIS seems not to differ between the conservative and demolitive approaches (RR = 0.48, 95% CI 0.09–2.41, *p* = 0.37).

In addition, conservative management should not determine a higher risk of invasive recurrence. The results of this systematic review and meta-analysis have highlighted how the risk of invasive disease during follow-up seems not to differ between the conservative and demolitive approaches (RR = 1.67, 95% CI 0.82–3.39, *p* = 0.16), with a pooled incidence of 1.3% for the conservative treatment. This incidence is slightly higher than the pooled incidence of 0.9% for invasive recurrence reported in the systematic review by Baalbergen et al., which only included studies about conservative treatment [11]. Similar results were found for squamous pre-invasive lesions in the review of Soutter et al. about follow-up after cervical intraepithelial neoplasia (CIN) treatment, where no difference was found in the incidence of invasive recurrence between hysterectomy and conservative treatment (39 vs. 56 per 100,000 patient-years, RR = 0.69, 95% CI 0.27–1.44, *p* = 0.33) [44].

Patients subjected to conservative management seem to present a higher risk of AIS recurrence during follow-up (RR = 8.44, 95% CI 3.36–21.19, *p* < 0.001). This higher risk was expected, given that the glandular epithelium and the cervix itself are maintained in the conservative approach. In this regard, Soutter et al. described a similar situation regarding CIN treatment [44]. Indeed, the authors reported that the incidence of post-treatment disease after CIN treatment was lower in patients subjected to hysterectomy in comparison to local therapy (397 vs. 1605 per 100,000 patient-years, RR = 0.25, 95% CI 0.19–0.32) [44].

Even if the relative risk of AIS recurrence seems to favor hysterectomy, the absolute risk must also be taken into account when counseling AIS patients with reproductive desires. In this systematic review and meta-analysis, the pooled risk of AIS recurrence for conservative management was 3.8%. This risk level is comparable to the risk of CIN2+ recurrence after cervical excision, which is reported to be 5.3–5.8% [45,46,47] and is widely accepted since the gold standard for CIN2+ is the excisional treatment, regardless of reproductive desire [12]. However, it should be acknowledged that AIS and CIN have different clinical behaviors. AIS is localized higher in the cervical canal, with extension to the glandular crypts, thus limiting the diagnostic performance of cervical cytology [48]. Even if the human papillomavirus (HPV) test seems to present a better predictive value during follow-up after conservative management [49], the unspecific colposcopic features of AIS [15,16] could pose a diagnostic challenge in the case of HPV test positivity. Therefore, the identification of an AIS recurrence during follow-up after conservative management may be more complex than identifying a CIN recurrence.

The lack of association between conservative treatment modality and risk of recurrence or residual is in line with the 2017 systematic review and meta-analysis of Jiang et al., highlighting that LEEP appeared to be as effective as CKC in terms of residual and recurrence [50]. Margin positivity was reported as a risk factor for recurrence after conservative treatment for AIS [11]. Our meta-regression did not show an association for margin positivity at first excision. This could be explained by the fact that the included studies reported data about surgical margin positivity at the first excision but did not stratify the results according to re-excision. Thus, a higher percentage of patients may have been sent to follow-up after obtaining negative margins at the second or third excision, with a lower risk of recurrence.

### 4.3. Strengths and Limitations

This is the first systematic review and meta-analysis that has evaluated the clinical outcomes of AIS according to the modality of treatment, including a comparator (hysterectomy) as an integral part of the analysis. Even if this choice may have limited the number of included studies, it was necessary to adhere strictly to the PICO model. Nevertheless, some limitations should be recognized. Firstly, all included studies were retrospective. In addition, more than 80% of included studies presented a serious risk of bias, mainly due to a lack of control for confounders or missing data. Those factors determined a “very low” certainty of evidence for the four outcomes, thus requiring caution in interpreting the results. The significant time span of recruitment of the included studies (1984–2023) was undoubtedly associated with heterogeneous clinical management. For example, endocervical curettage was not systematically performed, and follow-up strategies were different, with limited reported use of HPV tests. The follow-up duration varied across studies, making it challenging to evaluate recurrence outcomes for fixed time intervals or beyond the maximum follow-up period of the included studies. The lack of reported data about relevant factors such as HPV positivity did not allow us to perform additional analyses. We have considered only glandular recurrences to improve consistency between initial and recurrent histopathological diagnoses. However, the number of squamous recurrences was low and would not likely have altered the results. It was impossible to discriminate the histopathological AIS subtype (HPV-associated/HPV-independent or usual-type/not usual-type). Regarding the outcome “AIS recurrence”, the Egger test indicated potential publication bias, suggesting that smaller studies with non-significant results may be underrepresented in the literature.

### 4.4. Implications

Our results support the safety of choice for a conservative approach in AIS patients and may be helpful in counseling and treatment planning, particularly in patients with childbearing desires. However, the “very low” certainty of evidence cannot allow defining a strong recommendation but rather a conditional one. Indeed, prospective trials with standardized clinical management and methodology regarding long-term outcomes and follow-up strategies, including HPV tests, are needed. Designing randomized controlled trials will probably be challenging since randomization between conservative and demolitive treatment could potentially be performed only after childbearing completion. Future studies may also contribute to answering more specific clinical questions. For example, the Cancer Council of Australia did not find studies in 2022 to answer their question about comparing completion hysterectomy or ongoing surveillance by co-testing with cytology. Indeed, they based their recommendation “In women who have been treated for AIS by excision, with clear margins, there is no evidence to support completion hysterectomy. In this situation, hysterectomy is not recommended” on expert opinion [51].

## 5. Conclusions

The safety profile of a conservative approach for patients diagnosed with AIS seems not to differ from that of a demolitive approach in terms of invasive residual or recurrence. However, conservative management seems to be associated with a higher risk of AIS recurrence during follow-up. Clinicians should consider these aspects in managing patients with a histopathological diagnosis of AIS. The higher risk of AIS recurrence should be explained, also taking into account the absolute risk of recurrence and the need for strict and adequate follow-up. In the case of margin positivity, repeat conization did not expose the patient to a higher residual risk and could be chosen as an effective alternative. Follow-up strategies should be implemented to detect recurrence early.

## Figures and Tables

**Table 1:** Characteristics of the included studies.

First Author	Year	Country	Study Design	Study Period
Azodi et al. [24]	1999	USA	Retrospective	1988–1996
Baalbergen et al. [25]	2015	Holland	Retrospective	1989–2012
Bryson et al. [26]	2004	Canada	Retrospective	1990–2003
Dalrymple et al. [27]	2008	Australia	Retrospective	-
Dostalek et al. [28]	2023	Czech Republic	Retrospective	2002–2023
Giannella et al. [29]	2022	Italy	Retrospective	2012–2016
Hwang et al. [30]	2004	Canada	Retrospective	1980–2002
Im et al. [31]	1995	USA	Retrospective	1988–1994
Li et al. [32]	2013	USA	Retrospective	2000–2010
Liu et al. [33]	2022	China	Retrospective	2007–2021
Omnes et al. [34]	2003	France	Retrospective	1974–2000
Schaafsma et al. [35]	2025	The Netherlands	Retrospective	1990–2021
Shin et al. [36]	2000	USA	Retrospective	1987–1999
Song et al. [37]	2015	Republic of Korea	Retrospective	2000–2011
Tay et al. [38]	1999	Singapore	Retrospective	1991–1996
Taylor et al. [39]	2014	USA	Retrospective	1998–2011
Wang et al. [40]	2020	China	Retrospective	2002–2018
Wolf et al. [41]	1997	USA	Retrospective	1984–1993
Young et al. [42]	2007	USA	Retrospective	1998–2006

**Table 2:** Intervention details for each included study.

First Author	Conservative Treatment	Demolitive Treatment
Azodi et al. [24]	CKC; LEEP; LC ± ECC	Piver ARH type I-III; VH; LAVH.
Baalbergen et al. [25]	CKC; LEEP; LC	Hysterectomy
Bryson et al. [26]	LEEP	Hysterectomy
Dalrymple et al. [27]	CKC; LC	LAVH; RH ± PLND
Dostalek et al. [28]	LEEP; CKC; SVT	Hysterectomy
Giannella et al. [29]	CKC; LEEP; LC	RH ± LPND
Hwang et al. [30]	CKC; LEEP; LC	RH
Im et al. [31]	CKC; LEEP; LC	EH; RH;VH
Li et al. [32]	Conization	Hysterectomy
Liu et al. [33]	CKC; LEEP	Hysterectomy
Omnes et al. [34]	CKC; LEEP; SVT	SH; RH
Schaafsma et al. [35]	CKC, LLETZ	Hysterectomy
Shin et al. [36]	CKC; LEEP	Hysterectomy
Song et al. [37]	LEEP	Hysterectomy
Tay et al. [38]	CKC; LLETZ; LC	Hysterectomy
Taylor et al. [39]	CKC; LEEP	Hysterectomy
Wang et al. [40]	CKC; ESC	Hysterectomy
Wolf et al. [41]	CKC; LEEP; LC	SH; RH
Young et al. [42]	CKC; LEEP; LC	Hysterectomy

CKC: cold-knife conization; ESC: electrosurgical conization; LEEP: loop electrosurgical excision procedure; LC: laser conization; LLETZ: large loop excision transformation zone; ECC: endocervical curettage; SVT: simple vaginal trachelectomy; LPND: systematic pelvic lymphadenectomy; ARH: abdominal radical hysterectomy I-II-III sec. Piver’s classification. VH: vaginal hysterectomy; LAVH: laparoscopically assisted vaginal hysterectomy; LPND: systematic pelvic lymphadenectomy; RH: radical hysterectomy; EH: extrafascial hysterectomy; SH: simple hysterectomy.

**Table 3:** Certainty assessment and finding summary table for the study outcomes.

Certainty Assessment							Summary of Findings				
Participants (Studies)	Risk of Bias	Inconsistency	Indirectness	Imprecision	Publication Bias	Overall Certainty of Evidence	Study Event Rates (%)		Relative Effect (95% CI)	Anticipated Absolute Effects	
							With Demolitive Treatment	With Conservative Treatment		Risk with Demolitive Treatment	Risk Difference with Conservative Treatment
**AIS recurrence**											
5513 (11 non-randomized studies)	serious ^a^	not serious	not serious	serious ^b^	none	Very Low ^a,b^	1/1142 (0.1%)	166/4371 (3.8%)	RR 8.44 (3.36 to 21.19)	1 per 1.000	7 more per 1.000 (from 2 more to 18 more)
**Invasive recurrence**											
5070 (10 non-randomized studies)	serious ^c^	not serious	not serious	serious ^d^	none	Very Low ^c,d^	7/871 (0.8%)	55/4199 (1.3%)	RR 1.67 (0.82 to 3.39)	8 per 1.000	5 more per 1.000 (from 1 fewer to 19 more)
**AIS residual**											
231 (9 non-randomized studies)	serious ^e^	not serious	not serious	serious ^f^	none	Very Low ^e,f^	67/166 (40.4%)	26/65 (40.0%)	RR 0.89 (0.62 to 1.26)	404 per 1.000	44 fewer per 1.000 (from 153 fewer to 105 more)
**Invasive residual**											
123 (3 non-randomized studies)	serious ^g^	not serious	not serious	serious ^h^	none	Very Low ^g,h^	10/104 (9.6%)	0/19 (0.0%)	RR 0.48 (0.09 to 2.41)	96 per 1.000	50 fewer per 1.000 (from 88 fewer to 136 more)

CI: confidence interval; RR: risk ratio. Explanations: ^a^ Risk of bias was deemed as “serious” for this outcome since 90% of studies resulted in a serious risk of bias according to ROBINS-I. ^b^ Imprecision was deemed as “serious” given the large confidence interval of RR. ^c^ Risk of bias was deemed as “serious” for this outcome since 88.9% of studies resulted in a serious risk of bias according to ROBINS-I. ^d^ Imprecision was deemed as “serious” given the low number of events in both conservative and demolitive treatment. ^e^ Risk of bias was deemed as “serious” for this outcome since 88.9% of studies resulted in a serious risk of bias according to ROBINS-I. ^f^ Imprecision was deemed as “serious” given the large confidence interval of RR. ^g^ Risk of bias was deemed as “serious” for this outcome since 66.7% of studies resulted in a serious risk of bias according to ROBINS-I. ^h^ Imprecision was deemed as “serious” given the large confidence interval of RR.

## Data Availability

Data are contained within the article and Supplementary Materials.

## Supplementary Materials

The following supporting information can be downloaded at https://www.mdpi.com/article/10.3390/cancers17111839/s1: Supplementary File S1—Preferred Reporting Items for Systematic Reviews and Meta-Analyses (PRISMA) checklist. Supplementary File S2—Meta-analyses Of Observational Studies in Epidemiology (MOOSE) checklist. Supplementary File S3—complete risk of bias evaluation. Supplementary File S4—Patient characteristics of the included studies. Supplementary File S5—Clinical characteristics of the included studies. Supplementary File S6—Funnel plots for recurrence during follow-up and residual after positive margin.

## Abbreviations

The following abbreviations are used in this manuscript:

AIS	Adenocarcinoma In Situ
CI	Confidence Interval
CIN	Cervical Intraepithelial Neoplasia
GRADE	Grades of Recommendation, Assessment, Development and Evaluation
HPV	Human Papillomavirus
MOOSE	Meta-analyses Of Observational Studies in Epidemiology
PRISMA	Preferred Reporting Items for Systematic Reviews and Meta-Analyses
PROSPERO	The International Prospective Register of Systematic Reviews
RR	Risk Ratio
