# Supplementary material for: Clinical Outcomes of Cervical Adenocarcinoma In Situ According to Conservative or Demolitive Treatment: A Systematic Review and Meta-Analysis
Source: Cancers (Basel). 2025 May 30;17(11):1839. doi: 10.3390/cancers17111839 (PMC12153624; doi:10.3390/cancers17111839)
Supplement: Supplementary file 1 [file cancers-17-01839-s001.zip › Revision Supplementary File S2.pdf]

MOOSE checklist

Clinical outcomes of cervical adenocarcinoma in situ according to conservative or demolitive treatment: a systematic review and meta-analysis

Item No	Recommendation	Reported on page No
**Reporting of**	**background should include**	
1	Problem definition	3
2	Hypothesis statement	3
3	Description of study outcome(s)	4
4	Type of exposure or intervention used	4
5	Type of study designs used	3
6	Study population	4
**Reporting of**	**search strategy should include**	
7	Qualifications of searchers (eg, librarians and investigators)	4
8	Search strategy, including time period included in the synthesis and key words	3-4
9	Effort to include all available studies, including contact with authors	4
10	Databases and registries searched	3
11	Search software used, name and version, including special features used (eg, explosion)	NA
12	Use of hand searching (eg, reference lists of obtained articles)	3
13	List of citations located and those excluded, including justification	3-4
14	Method of addressing articles published in languages other than English	3-4
15	Method of handling abstracts and unpublished studies	3-4
16	Description of any contact with authors	3-4
**Reporting of**	**methods should include**	
17	Description of relevance or appropriateness of studies assembled for assessing the hypothesis to be tested	4-5
18	Rationale for the selection and coding of data (eg, sound clinical principles or convenience)	4-5
19	Documentation of how data were classified and coded (eg, multiple raters, blinding and interrater reliability)	4-5
20	Assessment of confounding (eg, comparability of cases and controls in studies where appropriate)	4-5
21	Assessment of study quality, including blinding of quality assessors, stratification or regression on possible predictors of study results	4
22	Assessment of heterogeneity	5
23	Description of statistical methods (eg, complete description of fixed or random effects models, justification of whether the chosen models account for predictors of study results, dose-response models, or cumulative meta-analysis) in sufficient detail to be replicated	4-5
24	Provision of appropriate tables and graphics	4-5
**Reporting of**	**results should include**	
25	Graphic summarizing individual study estimates and overall estimate	Figure 1-2
26	Table giving descriptive information for each study included	Table 1-2
27	Results of sensitivity testing (eg, subgroup analysis)	NA
28	Indication of statistical uncertainty of findings	9
**Reporting of**	**discussion should include**	
29	Quantitative assessment of bias (eg, publication bias)	15
30	Justification for exclusion (eg, exclusion of non-English language citations)	NA
31	Assessment of quality of included studies	14-15
**Reporting of**	conclusions should include	
32	Consideration of alternative explanations for observed results	14
33	Generalization of the conclusions (ie, appropriate for the data presented and within the domain of the literature review)	14

MOOSE checklist

Clinical outcomes of cervical adenocarcinoma in situ according to conservative or demolitive treatment: a systematic review and meta-analysis

34	Guidelines for future research	15-16
35	Disclosure of funding source	17
