# Supplementary material for: Clinical Outcomes of Cervical Adenocarcinoma In Situ According to Conservative or Demolitive Treatment: A Systematic Review and Meta-Analysis
Source: Cancers (Basel). 2025 May 30;17(11):1839. doi: 10.3390/cancers17111839 (PMC12153624; doi:10.3390/cancers17111839)
Supplement: Supplementary file 1 [file cancers-17-01839-s001.zip › Revision Supplementary File S3.pdf]

The Risk Of Bias In Non-randomized Studies – of Interventions (ROBINS-I) assessment tool

(version for cohort-type studies)

Version 19 September 2016

This work is licensed under a Creative Commons Attribution-NonCommercial-NoDerivatives 4.0 International License.

Clinical outcomes of cervical adenocarcinoma in situ according to conservative or demolitive treatment: a systematic review and meta-analysis

ROBINS-I tool (Stage I): At protocol stage

Specify the review question

Participants	Patients with histopathological diagnosis of AIS after cervical excision
Experimental intervention	Conservative treatment
Comparator	Demolitive treatment (hysterectomy)
Outcomes	Disease recurrence during follow-up<br>Residual disease (at the second conization after margin positivity at the first excision or at the hysterectomy specimen)

List the confounding domains relevant to all or most studies

Age

List co-interventions that could be different between intervention groups and that could impact on outcomes

None

ROBINS-I tool (Stage II): For each study

Azodi 1999

Specify a target randomized trial specific to the study

Design	Individually randomized / Cluster randomized / Matched (e.g. cross-over)
Participants	Patients with histopathological diagnosis of AIS after cervical excision
Experimental intervention	Conservative treatment
Comparator	Demolitive treatment (hysterectomy)

Is your aim for this study…?

- ■ to assess the effect of *assignment to* intervention
- ☐ to assess the effect of *starting and adhering to* intervention

Specify the outcome

Specify which outcome is being assessed for risk of bias (typically from among those earmarked for the Summary of Findings table). Specify whether this is a proposed benefit or harm of intervention.

Recurrence
Residual

Specify the numerical result being assessed

In case of multiple alternative analyses being presented, specify the numeric result (e.g. RR = 1.52 (95% CI 0.83 to 2.77) and/or a reference (e.g. to a table, figure or paragraph) that uniquely defines the result being assessed.

Preliminary consideration of confounders

Complete a row for each important confounding domain (i) listed in the review protocol; and (ii) relevant to the setting of this particular study, or which the study authors identified as potentially important.

"Important" confounding domains are those for which, in the context of this study, adjustment is expected to lead to a clinically important change in the estimated effect of the intervention. "Validity" refers to whether the confounding variable or variables fully measure the domain, while "reliability" refers to the precision of the measurement (more measurement error means less reliability).

**(i) Confounding domains listed in the review protocol**				
Confounding domain	Measured variable(s)	Is there evidence that controlling for this variable was unnecessary?*	Is the confounding domain measured validly and reliably by this variable (or these variables)?	OPTIONAL: Is failure to adjust for this variable (alone) expected to favour the experimental intervention or the comparator?
Age	Not evaluated as confounder	No	-	-

**(ii) Additional confounding domains relevant to the setting of this particular study, or which the study authors identified as important**				
Confounding domain	Measured variable(s)	Is there evidence that controlling for this variable was unnecessary?*	Is the confounding domain measured validly and reliably by this variable (or these variables)?	OPTIONAL: Is failure to adjust for this variable (alone) expected to favour the experimental intervention or the comparator?
-	-	-	-	-

* In the context of a particular study, variables can be demonstrated not to be confounders and so not included in the analysis: (a) if they are not predictive of the outcome; (b) if they are not predictive of intervention; or (c) because adjustment makes no or minimal difference to the estimated effect of the primary parameter. Note that "no statistically significant association" is not the same as "not predictive".

Preliminary consideration of co-interventions

Complete a row for each important co-intervention (i) listed in the review protocol; and (ii) relevant to the setting of this particular study, or which the study authors identified as important.

"Important" co-interventions are those for which, in the context of this study, adjustment is expected to lead to a clinically important change in the estimated effect of the intervention.

**(i) Co-interventions listed in the review protocol**		
Co-intervention	Is there evidence that controlling for this co-intervention was unnecessary (e.g. because it was not administered)?	Is presence of this co-intervention likely to favour outcomes in the experimental intervention or the comparator
None	-	-

**(ii) Additional co-interventions relevant to the setting of this particular study, or which the study authors identified as important**		
Co-intervention	Is there evidence that controlling for this co-intervention was unnecessary (e.g. because it was not administered)?	Is presence of this co-intervention likely to favour outcomes in the experimental intervention or the comparator
None	-	-

Risk of bias assessment

Responses underlined in green are potential markers for low risk of bias, and responses in red are potential markers for a risk of bias. Where questions relate only to sign posts to other questions, no formatting is used.

Signalling questions	Description	Response options
**Bias due to confounding**		
1.1 Is there potential for confounding of the effect of intervention in this study?<br>**If N/PN to 1.1:** the study can be considered to be at low risk of bias due to confounding and no further signalling questions need be considered		Y / PY / PN / N
**If Y/PY to 1.1**: determine whether there is a need to assess time-varying confounding:		
1.2. Was the analysis based on splitting participants' follow up time according to intervention received?<br>**If N/PN**, answer questions relating to baseline confounding (1.4 to 1.6)<br>**If Y/PY**, go to question 1.3.		NA / Y / PY / PN / N / NI
1.3. Were intervention discontinuations or switches likely to be related to factors that are prognostic for the outcome?<br>**If N/PN**, answer questions relating to baseline confounding (1.4 to 1.6)<br>**If Y/PY**, answer questions relating to both baseline and time-varying confounding (1.7 and 1.8)		NA / Y / PY / PN / N / NI

**Questions relating to baseline confounding only**		
1.4. Did the authors use an appropriate analysis method that controlled for all the important confounding domains?		NA / Y / PY / PN / N / NI
1.5. **If** Y/PY **to 1.4**: Were confounding domains that were controlled for measured validly and reliably by the variables available in this study?		NA / Y / PY / PN / N / NI
1.6. Did the authors control for any post-intervention variables that could have been affected by the intervention?		NA / Y / PY / PN / N / NI
**Questions relating to baseline and time-varying confounding**		
1.7. Did the authors use an appropriate analysis method that controlled for all the important confounding domains and for time-varying confounding?		NA / Y / PY / PN / N / NI
1.8. **If** Y/PY **to 1.7**: Were confounding domains that were controlled for measured validly and reliably by the variables available in this study?		NA / Y / PY / PN / N / NI
**Risk of bias judgement**		Low / Moderate / Serious / Critical / NI
Optional: What is the predicted direction of bias due to confounding?		Favours experimental / Favours comparator / Unpredictable

Bias in selection of participants into the study		
2.1. Was selection of participants into the study (or into the analysis) based on participant characteristics observed after the start of intervention?<br>**If N/PN to 2.1:** go to 2.4		Y / PY / PN / N / NI
2.2. **If Y/PY to 2.1**: Were the post-intervention variables that influenced selection likely to be associated with intervention?		NA / Y / PY / PN / N / NI
2.3 **If Y/PY to 2.2**: Were the post-intervention variables that influenced selection likely to be influenced by the outcome or a cause of the outcome?		NA / Y / PY / PN / N / NI
2.4. Do start of follow-up and start of intervention coincide for most participants?		Y / PY / PN / N / NI
2.5. **If Y/PY to 2.2 and 2.3, or N/PN to 2.4**: Were adjustment techniques used that are likely to correct for the presence of selection biases?		NA / Y / PY / PN / N / NI
**Risk of bias judgement**		Low / Moderate / Serious / Critical / NI
Optional: What is the predicted direction of bias due to selection of participants into the study?		Favours experimental / Favours comparator / Towards null /Away from null / Unpredictable

Bias in classification of interventions		
3.1 Were intervention groups clearly defined?		Y / PY / PN / N / NI
3.2 Was the information used to define intervention groups recorded at the start of the intervention?		Y / PY / PN / N / NI
3.3 Could classification of intervention status have been affected by knowledge of the outcome or risk of the outcome?		Y / PY / PN / N / NI
**Risk of bias judgement**		Low / Moderate / Serious / Critical / NI
Optional: What is the predicted direction of bias due to classification of interventions?		Favours experimental / Favours comparator / Towards null /Away from null / Unpredictable

**Bias due to deviations from intended interventions**		
**If your aim for this study is to assess the effect of assignment to intervention, answer questions 4.1 and 4.2**		
4.1. Were there deviations from the intended intervention beyond what would be expected in usual practice?		Y / PY / PN / N / NI
4.2. **If Y/PY to 4.1**: Were these deviations from intended intervention unbalanced between groups *and* likely to have affected the outcome?		NA / Y / PY / PN / N / NI
**If your aim for this study is to assess the effect of starting and adhering to intervention, answer questions 4.3 to 4.6**		
4.3. Were important co-interventions balanced across intervention groups?		Y / PY / PN / N / NI
4.4. Was the intervention implemented successfully for most participants?		Y / PY / PN / N / NI
4.5. Did study participants adhere to the assigned intervention regimen?		Y / PY / PN / N / NI
4.6. **If N/PN to 4.3, 4.4 or 4.5**: Was an appropriate analysis used to estimate the effect of starting and adhering to the intervention?		NA / Y / PY / PN / N / NI
**Risk of bias judgement**		Low / Moderate / Serious / Critical / NI
Optional: What is the predicted direction of bias due to deviations from the intended interventions?		Favours experimental / Favours comparator / Towards null /Away from null / Unpredictable

Bias due to missing data		
5.1 Were outcome data available for all, or nearly all, participants?		Y / PY / PN / N / NI
5.2 Were participants excluded due to missing data on intervention status?		Y / PY / PN / N / NI
5.3 Were participants excluded due to missing data on other variables needed for the analysis?		Y / PY / PN / N / NI
5.4 **If PN/N to 5.1, or Y/PY to 5.2 or 5.3**: Are the proportion of participants and reasons for missing data similar across interventions?		NA / Y / PY / PN / N / NI
5.5 **If PN/N to 5.1, or Y/PY to 5.2 or 5.3**: Is there evidence that results were robust to the presence of missing data?		NA / Y / PY / PN / N / NI
**Risk of bias judgement**		Low / Moderate / Serious / Critical / NI
Optional: What is the predicted direction of bias due to missing data?		Favours experimental / Favours comparator / Towards null /Away from null / Unpredictable

Bias in measurement of outcomes		
6.1 Could the outcome measure have been influenced by knowledge of the intervention received?		Y / PY / PN / N / NI
6.2 Were outcome assessors aware of the intervention received by study participants?		Y / PY / PN / N / NI
6.3 Were the methods of outcome assessment comparable across intervention groups?		Y / PY / PN / N / NI
6.4 Were any systematic errors in measurement of the outcome related to intervention received?		Y / PY / PN / N / NI
**Risk of bias judgement**		Low / Moderate / Serious / Critical / NI
Optional: What is the predicted direction of bias due to measurement of outcomes?		Favours experimental / Favours comparator / Towards null /Away from null / Unpredictable

**Bias in selection of the reported result**		
Is the reported effect estimate likely to be selected, on the basis of the results, from...<br>7.1. ... multiple outcome *measurements* within the outcome domain?		Y / PY / PN / N / NI
7.2 ... multiple *analyses* of the intervention-outcome relationship?		Y / PY / PN / N / NI
7.3 ... different *subgroups*?		Y / PY / PN / N / NI
**Risk of bias judgement**		Low / Moderate / Serious / Critical / NI
Optional: What is the predicted direction of bias due to selection of the reported result?		Favours experimental / Favours comparator / Towards null /Away from null / Unpredictable

**Overall bias**		
**Risk of bias judgement**		Low / Moderate / Serious / Critical / NI
Optional: What is the overall predicted direction of bias for this outcome?		Favours experimental / Favours comparator / Towards null /Away from null / Unpredictable

This work is licensed under a Creative Commons Attribution-NonCommercial-NoDerivatives 4.0 International License.

Domain	Recurrence	Residual
Bias due to confounding	Serious risk	Serious risk
Bias in selection of participants into the study	Low risk	Low risk
Bias in classification of interventions	Low risk	Low risk
Bias due to deviations from intended interventions	Low risk	Low risk
Bias due to missing data	Low risk	Low risk
Bias in measurement of outcomes	Low risk	Low risk
Bias in selection of the reported result	Moderate risk	Moderate risk
**Overall**	**Serious risk**	**Serious risk**

Baalbergen 2015

Specify a target randomized trial specific to the study

Design	Individually randomized / Cluster randomized / Matched (e.g. cross-over)
Participants	Patients with histopathological diagnosis of AIS after cervical excision
Experimental intervention	Conservative treatment
Comparator	Demolitive treatment (hysterectomy)

Is your aim for this study…?

- ■ to assess the effect of *assignment to* intervention
- ☐ to assess the effect of *starting and adhering to* intervention

Specify the outcome

Specify which outcome is being assessed for risk of bias (typically from among those earmarked for the Summary of Findings table). Specify whether this is a proposed benefit or harm of intervention.

Recurrence
Residual

Specify the numerical result being assessed

In case of multiple alternative analyses being presented, specify the numeric result (e.g. RR = 1.52 (95% CI 0.83 to 2.77) and/or a reference (e.g. to a table, figure or paragraph) that uniquely defines the result being assessed.

Preliminary consideration of confounders

Complete a row for each important confounding domain (i) listed in the review protocol; and (ii) relevant to the setting of this particular study, or which the study authors identified as potentially important.

"Important" confounding domains are those for which, in the context of this study, adjustment is expected to lead to a clinically important change in the estimated effect of the intervention. "Validity" refers to whether the confounding variable or variables fully measure the domain, while "reliability" refers to the precision of the measurement (more measurement error means less reliability).

**(i) Confounding domains listed in the review protocol**				
Confounding domain	Measured variable(s)	Is there evidence that controlling for this variable was unnecessary?*	Is the confounding domain measured validly and reliably by this variable (or these variables)?	OPTIONAL: Is failure to adjust for this variable (alone) expected to favour the experimental intervention or the comparator?
Age	Not evaluated as confounder	No	-	-

**(ii) Additional confounding domains relevant to the setting of this particular study, or which the study authors identified as important**				
Confounding domain	Measured variable(s)	Is there evidence that controlling for this variable was unnecessary?*	Is the confounding domain measured validly and reliably by this variable (or these variables)?	OPTIONAL: Is failure to adjust for this variable (alone) expected to favour the experimental intervention or the comparator?
-	-	-	-	-

* In the context of a particular study, variables can be demonstrated not to be confounders and so not included in the analysis: (a) if they are not predictive of the outcome; (b) if they are not predictive of intervention; or (c) because adjustment makes no or minimal difference to the estimated effect of the primary parameter. Note that "no statistically significant association" is not the same as "not predictive".

Preliminary consideration of co-interventions

Complete a row for each important co-intervention (i) listed in the review protocol; and (ii) relevant to the setting of this particular study, or which the study authors identified as important.

"Important" co-interventions are those for which, in the context of this study, adjustment is expected to lead to a clinically important change in the estimated effect of the intervention.

**(i) Co-interventions listed in the review protocol**		
Co-intervention	Is there evidence that controlling for this co-intervention was unnecessary (e.g. because it was not administered)?	Is presence of this co-intervention likely to favour outcomes in the experimental intervention or the comparator
None	-	-

**(ii) Additional co-interventions relevant to the setting of this particular study, or which the study authors identified as important**		
Co-intervention	Is there evidence that controlling for this co-intervention was unnecessary (e.g. because it was not administered)?	Is presence of this co-intervention likely to favour outcomes in the experimental intervention or the comparator
None	-	-

Risk of bias assessment

Responses underlined in green are potential markers for low risk of bias, and responses in red are potential markers for a risk of bias. Where questions relate only to sign posts to other questions, no formatting is used.

Signalling questions	Description	Response options
**Bias due to confounding**		
1.1 Is there potential for confounding of the effect of intervention in this study?<br>**If N/PN to 1.1:** the study can be considered to be at low risk of bias due to confounding and no further signalling questions need be considered		Y / PY / PN / N
**If Y/PY to 1.1**: determine whether there is a need to assess time-varying confounding:		
1.2. Was the analysis based on splitting participants' follow up time according to intervention received?<br>**If N/PN**, answer questions relating to baseline confounding (1.4 to 1.6)<br>**If Y/PY**, go to question 1.3.		NA / Y / PY / PN / N / NI
1.3. Were intervention discontinuations or switches likely to be related to factors that are prognostic for the outcome?<br>**If N/PN**, answer questions relating to baseline confounding (1.4 to 1.6)<br>**If Y/PY**, answer questions relating to both baseline and time-varying confounding (1.7 and 1.8)		NA / Y / PY / PN / N / NI

**Questions relating to baseline confounding only**		
1.4. Did the authors use an appropriate analysis method that controlled for all the important confounding domains?		NA / Y / PY / PN / N / NI
1.5. **If Y/PY to 1.4**: Were confounding domains that were controlled for measured validly and reliably by the variables available in this study?		NA / Y / PY / PN / N / NI
1.6. Did the authors control for any post-intervention variables that could have been affected by the intervention?		NA / Y / PY / PN / N / NI
**Questions relating to baseline and time-varying confounding**		
1.7. Did the authors use an appropriate analysis method that controlled for all the important confounding domains and for time-varying confounding?		NA / Y / PY / PN / N / NI
1.8. **If Y/PY to 1.7**: Were confounding domains that were controlled for measured validly and reliably by the variables available in this study?		NA / Y / PY / PN / N / NI
**Risk of bias judgement**		Low / Moderate / Serious / Critical / NI
Optional: What is the predicted direction of bias due to confounding?		Favours experimental / Favours comparator / Unpredictable

Bias in selection of participants into the study		
2.1. Was selection of participants into the study (or into the analysis) based on participant characteristics observed after the start of intervention?<br>**If N/PN to 2.1:** go to 2.4		Y / PY / PN / N / NI
2.2. **If Y/PY to 2.1**: Were the post-intervention variables that influenced selection likely to be associated with intervention?		NA / Y / PY / PN / N / NI
2.3 **If Y/PY to 2.2**: Were the post-intervention variables that influenced selection likely to be influenced by the outcome or a cause of the outcome?		NA / Y / PY / PN / N / NI
2.4. Do start of follow-up and start of intervention coincide for most participants?		Y / PY / PN / N / NI
2.5. **If Y/PY to 2.2 and 2.3, or N/PN to 2.4**: Were adjustment techniques used that are likely to correct for the presence of selection biases?		NA / Y / PY / PN / N / NI
**Risk of bias judgement**		Low / Moderate / Serious / Critical / NI
Optional: What is the predicted direction of bias due to selection of participants into the study?		Favours experimental / Favours comparator / Towards null /Away from null / Unpredictable

Bias in classification of interventions		
3.1 Were intervention groups clearly defined?		Y / PY / PN / N / NI
3.2 Was the information used to define intervention groups recorded at the start of the intervention?		Y / PY / PN / N / NI
3.3 Could classification of intervention status have been affected by knowledge of the outcome or risk of the outcome?		Y / PY / PN / N / NI
**Risk of bias judgement**		Low / Moderate / Serious / Critical / NI
Optional: What is the predicted direction of bias due to classification of interventions?		Favours experimental / Favours comparator / Towards null /Away from null / Unpredictable

Bias due to deviations from intended interventions		
**If your aim for this study is to assess the effect of assignment to intervention, answer questions 4.1 and 4.2**		
4.1. Were there deviations from the intended intervention beyond what would be expected in usual practice?		Y / PY / PN / N / NI
4.2. **If Y/PY to 4.1**: Were these deviations from intended intervention unbalanced between groups *and* likely to have affected the outcome?		NA / Y / PY / PN / N / NI
**If your aim for this study is to assess the effect of starting and adhering to intervention, answer questions 4.3 to 4.6**		
4.3. Were important co-interventions balanced across intervention groups?		Y / PY / PN / N / NI
4.4. Was the intervention implemented successfully for most participants?		Y / PY / PN / N / NI
4.5. Did study participants adhere to the assigned intervention regimen?		Y / PY / PN / N / NI
4.6. **If N/PN to 4.3, 4.4 or 4.5**: Was an appropriate analysis used to estimate the effect of starting and adhering to the intervention?		NA / Y / PY / PN / N / NI
**Risk of bias judgement**		Low / Moderate / Serious / Critical / NI
Optional: What is the predicted direction of bias due to deviations from the intended interventions?		Favours experimental / Favours comparator / Towards null /Away from null / Unpredictable

Bias due to missing data		
5.1 Were outcome data available for all, or nearly all, participants?		Y / PY / PN / N / NI
5.2 Were participants excluded due to missing data on intervention status?		Y / PY / PN / N / NI
5.3 Were participants excluded due to missing data on other variables needed for the analysis?		Y / PY / PN / N / NI
5.4 **If PN/N to 5.1, or Y/PY to 5.2 or 5.3**: Are the proportion of participants and reasons for missing data similar across interventions?		NA / Y / PY / PN / N / NI
5.5 **If PN/N to 5.1, or Y/PY to 5.2 or 5.3**: Is there evidence that results were robust to the presence of missing data?		NA / Y / PY / PN / N / NI
**Risk of bias judgement**		Low / Moderate / Serious / Critical / NI
Optional: What is the predicted direction of bias due to missing data?		Favours experimental / Favours comparator / Towards null /Away from null / Unpredictable

Bias in measurement of outcomes		
6.1 Could the outcome measure have been influenced by knowledge of the intervention received?		Y / PY / PN / N / NI
6.2 Were outcome assessors aware of the intervention received by study participants?		Y / PY / PN / N / NI
6.3 Were the methods of outcome assessment comparable across intervention groups?		Y / PY / PN / N / NI
6.4 Were any systematic errors in measurement of the outcome related to intervention received?		Y / PY / PN / N / NI
**Risk of bias judgement**		Low / Moderate / Serious / Critical / NI
Optional: What is the predicted direction of bias due to measurement of outcomes?		Favours experimental / Favours comparator / Towards null /Away from null / Unpredictable

**Bias in selection of the reported result**		
Is the reported effect estimate likely to be selected, on the basis of the results, from...<br>7.1. ... multiple outcome *measurements* within the outcome domain?		Y / PY / PN / N / NI
7.2 ... multiple *analyses* of the intervention-outcome relationship?		Y / PY / PN / N / NI
7.3 ... different *subgroups*?		Y / PY / PN / N / NI
**Risk of bias judgement**		Low / Moderate / Serious / Critical / NI
Optional: What is the predicted direction of bias due to selection of the reported result?		Favours experimental / Favours comparator / Towards null /Away from null / Unpredictable

**Overall bias**		
**Risk of bias judgement**		Low / Moderate / Serious / Critical / NI
Optional: What is the overall predicted direction of bias for this outcome?		Favours experimental / Favours comparator / Towards null /Away from null / Unpredictable

This work is licensed under a Creative Commons Attribution-NonCommercial-NoDerivatives 4.0 International License.

Domain	Recurrence
Bias due to confounding	Serious risk
Bias in selection of participants into the study	Low risk
Bias in classification of interventions	Low risk
Bias due to deviations from intended interventions	Low risk
Bias due to missing data	Serious risk
Bias in measurement of outcomes	Low risk
Bias in selection of the reported result	Moderate risk
**Overall**	**Serious risk**

Bryson 2004

Specify a target randomized trial specific to the study

Design	Individually randomized / Cluster randomized / Matched (e.g. cross-over)
Participants	Patients with histopathological diagnosis of AIS after cervical excision
Experimental intervention	Conservative treatment
Comparator	Demolitive treatment (hysterectomy)

Is your aim for this study…?

- ■ to assess the effect of *assignment to* intervention
- ☐ to assess the effect of *starting and adhering to* intervention

Specify the outcome

Specify which outcome is being assessed for risk of bias (typically from among those earmarked for the Summary of Findings table). Specify whether this is a proposed benefit or harm of intervention.

Recurrence
Residual

Specify the numerical result being assessed

In case of multiple alternative analyses being presented, specify the numeric result (e.g. RR = 1.52 (95% CI 0.83 to 2.77) and/or a reference (e.g. to a table, figure or paragraph) that uniquely defines the result being assessed.

Preliminary consideration of confounders

Complete a row for each important confounding domain (i) listed in the review protocol; and (ii) relevant to the setting of this particular study, or which the study authors identified as potentially important.

"Important" confounding domains are those for which, in the context of this study, adjustment is expected to lead to a clinically important change in the estimated effect of the intervention. "Validity" refers to whether the confounding variable or variables fully measure the domain, while "reliability" refers to the precision of the measurement (more measurement error means less reliability).

**(i) Confounding domains listed in the review protocol**				
Confounding domain	Measured variable(s)	Is there evidence that controlling for this variable was unnecessary?*	Is the confounding domain measured validly and reliably by this variable (or these variables)?	OPTIONAL: Is failure to adjust for this variable (alone) expected to favour the experimental intervention or the comparator?
Age	Not evaluated as confounder	No	-	-

**(ii) Additional confounding domains relevant to the setting of this particular study, or which the study authors identified as important**				
Confounding domain	Measured variable(s)	Is there evidence that controlling for this variable was unnecessary?*	Is the confounding domain measured validly and reliably by this variable (or these variables)?	OPTIONAL: Is failure to adjust for this variable (alone) expected to favour the experimental intervention or the comparator?
-	-	-	-	-

* In the context of a particular study, variables can be demonstrated not to be confounders and so not included in the analysis: (a) if they are not predictive of the outcome; (b) if they are not predictive of intervention; or (c) because adjustment makes no or minimal difference to the estimated effect of the primary parameter. Note that "no statistically significant association" is not the same as "not predictive".

Preliminary consideration of co-interventions

Complete a row for each important co-intervention (i) listed in the review protocol; and (ii) relevant to the setting of this particular study, or which the study authors identified as important.

"Important" co-interventions are those for which, in the context of this study, adjustment is expected to lead to a clinically important change in the estimated effect of the intervention.

**(i) Co-interventions listed in the review protocol**		
Co-intervention	Is there evidence that controlling for this co-intervention was unnecessary (e.g. because it was not administered)?	Is presence of this co-intervention likely to favour outcomes in the experimental intervention or the comparator
None	-	-

**(ii) Additional co-interventions relevant to the setting of this particular study, or which the study authors identified as important**		
Co-intervention	Is there evidence that controlling for this co-intervention was unnecessary (e.g. because it was not administered)?	Is presence of this co-intervention likely to favour outcomes in the experimental intervention or the comparator
None	-	-

Risk of bias assessment

Responses underlined in green are potential markers for low risk of bias, and responses in red are potential markers for a risk of bias. Where questions relate only to sign posts to other questions, no formatting is used.

Signalling questions	Description	Response options
**Bias due to confounding**		
1.1 Is there potential for confounding of the effect of intervention in this study?<br><br>**If N/PN to 1.1:** the study can be considered to be at low risk of bias due to confounding and no further signalling questions need be considered		Y / PY / PN / N
**If Y/PY to 1.1**: determine whether there is a need to assess time-varying confounding:		
1.2. Was the analysis based on splitting participants' follow up time according to intervention received?<br><br>**If N/PN**, answer questions relating to baseline confounding (1.4 to 1.6)<br><br>**If Y/PY**, go to question 1.3.		NA / Y / PY / PN / N / NI
1.3. Were intervention discontinuations or switches likely to be related to factors that are prognostic for the outcome?<br><br>**If N/PN**, answer questions relating to baseline confounding (1.4 to 1.6)<br><br>**If Y/PY**, answer questions relating to both baseline and time-varying confounding (1.7 and 1.8)		NA / Y / PY / PN / N / NI

**Questions relating to baseline confounding only**		
1.4. Did the authors use an appropriate analysis method that controlled for all the important confounding domains?		NA / Y / PY / PN / N / NI
1.5. **If Y/PY to 1.4**: Were confounding domains that were controlled for measured validly and reliably by the variables available in this study?		NA / Y / PY / PN / N / NI
1.6. Did the authors control for any post-intervention variables that could have been affected by the intervention?		NA / Y / PY / PN / N / NI
**Questions relating to baseline and time-varying confounding**		
1.7. Did the authors use an appropriate analysis method that controlled for all the important confounding domains and for time-varying confounding?		NA / Y / PY / PN / N / NI
1.8. **If Y/PY to 1.7**: Were confounding domains that were controlled for measured validly and reliably by the variables available in this study?		NA / Y / PY / PN / N / NI
**Risk of bias judgement**		Low / Moderate / Serious / Critical / NI
Optional: What is the predicted direction of bias due to confounding?		Favours experimental / Favours comparator / Unpredictable

Bias in selection of participants into the study		
2.1. Was selection of participants into the study (or into the analysis) based on participant characteristics observed after the start of intervention?<br>**If N/PN to 2.1:** go to 2.4		Y / PY / PN / N / NI
2.2. **If Y/PY to 2.1**: Were the post-intervention variables that influenced selection likely to be associated with intervention?		NA / Y / PY / PN / N / NI
2.3 **If Y/PY to 2.2**: Were the post-intervention variables that influenced selection likely to be influenced by the outcome or a cause of the outcome?		NA / Y / PY / PN / N / NI
2.4. Do start of follow-up and start of intervention coincide for most participants?		Y / PY / PN / N / NI
2.5. **If Y/PY to 2.2 and 2.3, or N/PN to 2.4**: Were adjustment techniques used that are likely to correct for the presence of selection biases?		NA / Y / PY / PN / N / NI
**Risk of bias judgement**		Low / Moderate / Serious / Critical / NI
Optional: What is the predicted direction of bias due to selection of participants into the study?		Favours experimental / Favours comparator / Towards null /Away from null / Unpredictable

Bias in classification of interventions		
3.1 Were intervention groups clearly defined?		Y / PY / PN / N / NI
3.2 Was the information used to define intervention groups recorded at the start of the intervention?		Y / PY / PN / N / NI
3.3 Could classification of intervention status have been affected by knowledge of the outcome or risk of the outcome?		Y / PY / PN / N / NI
**Risk of bias judgement**		Low / Moderate / Serious / Critical / NI
Optional: What is the predicted direction of bias due to classification of interventions?		Favours experimental / Favours comparator / Towards null /Away from null / Unpredictable

Bias due to deviations from intended interventions		
**If your aim for this study is to assess the effect of assignment to intervention, answer questions 4.1 and 4.2**		
4.1. Were there deviations from the intended intervention beyond what would be expected in usual practice?		Y / PY / PN / N / NI
4.2. **If Y/PY to 4.1**: Were these deviations from intended intervention unbalanced between groups *and* likely to have affected the outcome?		NA / Y / PY / PN / N / NI
**If your aim for this study is to assess the effect of starting and adhering to intervention, answer questions 4.3 to 4.6**		
4.3. Were important co-interventions balanced across intervention groups?		Y / PY / PN / N / NI
4.4. Was the intervention implemented successfully for most participants?		Y / PY / PN / N / NI
4.5. Did study participants adhere to the assigned intervention regimen?		Y / PY / PN / N / NI
4.6. **If N/PN to 4.3, 4.4 or 4.5**: Was an appropriate analysis used to estimate the effect of starting and adhering to the intervention?		NA / Y / PY / PN / N / NI
**Risk of bias judgement**		Low / Moderate / Serious / Critical / NI
Optional: What is the predicted direction of bias due to deviations from the intended interventions?		Favours experimental / Favours comparator / Towards null /Away from null / Unpredictable

Bias due to missing data		
5.1 Were outcome data available for all, or nearly all, participants?		Y / PY / PN / N / NI
5.2 Were participants excluded due to missing data on intervention status?		Y / PY / PN / N / NI
5.3 Were participants excluded due to missing data on other variables needed for the analysis?		Y / PY / PN / N / NI
5.4 **If PN/N to 5.1, or Y/PY to 5.2 or 5.3**: Are the proportion of participants and reasons for missing data similar across interventions?		NA / Y / PY / PN / N / NI
5.5 **If PN/N to 5.1, or Y/PY to 5.2 or 5.3**: Is there evidence that results were robust to the presence of missing data?		NA / Y / PY / PN / N / NI
**Risk of bias judgement**		Low / Moderate / Serious / Critical / NI
Optional: What is the predicted direction of bias due to missing data?		Favours experimental / Favours comparator / Towards null /Away from null / Unpredictable

Bias in measurement of outcomes		
6.1 Could the outcome measure have been influenced by knowledge of the intervention received?		Y / PY / PN / N / NI
6.2 Were outcome assessors aware of the intervention received by study participants?		Y / PY / PN / N / NI
6.3 Were the methods of outcome assessment comparable across intervention groups?		Y / PY / PN / N / NI
6.4 Were any systematic errors in measurement of the outcome related to intervention received?		Y / PY / PN / N / NI
**Risk of bias judgement**		Low / Moderate / Serious / Critical / NI
Optional: What is the predicted direction of bias due to measurement of outcomes?		Favours experimental / Favours comparator / Towards null /Away from null / Unpredictable

Bias in selection of the reported result		
Is the reported effect estimate likely to be selected, on the basis of the results, from...		
7.1. ... multiple outcome *measurements* within the outcome domain?		Y / PY / PN / N / NI
7.2 ... multiple *analyses* of the intervention-outcome relationship?		Y / PY / PN / N / NI
7.3 ... different *subgroups*?		Y / PY / PN / N / NI
**Risk of bias judgement**		Low / Moderate / Serious / Critical / NI
Optional: What is the predicted direction of bias due to selection of the reported result?		Favours experimental / Favours comparator / Towards null /Away from null / Unpredictable

Overall bias		
**Risk of bias judgement**		Low / Moderate / Serious / Critical / NI
Optional: What is the overall predicted direction of bias for this outcome?		Favours experimental / Favours comparator / Towards null /Away from null / Unpredictable

This work is licensed under a Creative Commons Attribution-NonCommercial-NoDerivatives 4.0 International License.

**Domain**	**Recurrence**	**Residual**
Bias due to confounding	Serious risk	Serious risk
Bias in selection of participants into the study	Low risk	Low risk
Bias in classification of interventions	Low risk	Low risk
Bias due to deviations from intended interventions	Low risk	Low risk
Bias due to missing data	Low risk	Low risk
Bias in measurement of outcomes	Low risk	Low risk
Bias in selection of the reported result	Moderate risk	Moderate risk
**Overall**	**Serious risk**	**Serious risk**

Dalrymple 2007

Specify a target randomized trial specific to the study

Design	Individually randomized / Cluster randomized / Matched (e.g. cross-over)
Participants	Patients with histopathological diagnosis of AIS after cervical excision
Experimental intervention	Conservative treatment
Comparator	Demolitive treatment (hysterectomy)

Is your aim for this study…?

- ■ to assess the effect of *assignment to* intervention
- ☐ to assess the effect of *starting and adhering to* intervention

Specify the outcome

Specify which outcome is being assessed for risk of bias (typically from among those earmarked for the Summary of Findings table). Specify whether this is a proposed benefit or harm of intervention.

Recurrence<br>Residual

Specify the numerical result being assessed

In case of multiple alternative analyses being presented, specify the numeric result (e.g. RR = 1.52 (95% CI 0.83 to 2.77) and/or a reference (e.g. to a table, figure or paragraph) that uniquely defines the result being assessed.

Preliminary consideration of confounders

Complete a row for each important confounding domain (i) listed in the review protocol; and (ii) relevant to the setting of this particular study, or which the study authors identified as potentially important.

"Important" confounding domains are those for which, in the context of this study, adjustment is expected to lead to a clinically important change in the estimated effect of the intervention. "Validity" refers to whether the confounding variable or variables fully measure the domain, while "reliability" refers to the precision of the measurement (more measurement error means less reliability).

**(i) Confounding domains listed in the review protocol**				
Confounding domain	Measured variable(s)	Is there evidence that controlling for this variable was unnecessary?*	Is the confounding domain measured validly and reliably by this variable (or these variables)?	OPTIONAL: Is failure to adjust for this variable (alone) expected to favour the experimental intervention or the comparator?
Age	Not evaluated as confounder	No	-	-

**(ii) Additional confounding domains relevant to the setting of this particular study, or which the study authors identified as important**				
Confounding domain	Measured variable(s)	Is there evidence that controlling for this variable was unnecessary?*	Is the confounding domain measured validly and reliably by this variable (or these variables)?	OPTIONAL: Is failure to adjust for this variable (alone) expected to favour the experimental intervention or the comparator?
-	-	-	-	-

* In the context of a particular study, variables can be demonstrated not to be confounders and so not included in the analysis: (a) if they are not predictive of the outcome; (b) if they are not predictive of intervention; or (c) because adjustment makes no or minimal difference to the estimated effect of the primary parameter. Note that "no statistically significant association" is not the same as "not predictive".

Preliminary consideration of co-interventions

Complete a row for each important co-intervention (i) listed in the review protocol; and (ii) relevant to the setting of this particular study, or which the study authors identified as important.

"Important" co-interventions are those for which, in the context of this study, adjustment is expected to lead to a clinically important change in the estimated effect of the intervention.

**(i) Co-interventions listed in the review protocol**		
Co-intervention	Is there evidence that controlling for this co-intervention was unnecessary (e.g. because it was not administered)?	Is presence of this co-intervention likely to favour outcomes in the experimental intervention or the comparator
None	-	-

**(ii) Additional co-interventions relevant to the setting of this particular study, or which the study authors identified as important**		
Co-intervention	Is there evidence that controlling for this co-intervention was unnecessary (e.g. because it was not administered)?	Is presence of this co-intervention likely to favour outcomes in the experimental intervention or the comparator
None	-	-

Risk of bias assessment

Responses underlined in green are potential markers for low risk of bias, and responses in red are potential markers for a risk of bias. Where questions relate only to sign posts to other questions, no formatting is used.

Signalling questions	Description	Response options
**Bias due to confounding**		
1.1 Is there potential for confounding of the effect of intervention in this study?<br>**If N/PN to 1.1:** the study can be considered to be at low risk of bias due to confounding and no further signalling questions need be considered		Y / PY / PN / N
**If Y/PY to 1.1**: determine whether there is a need to assess time-varying confounding:		
1.2. Was the analysis based on splitting participants' follow up time according to intervention received?<br>**If N/PN**, answer questions relating to baseline confounding (1.4 to 1.6)<br>**If Y/PY**, go to question 1.3.		NA / Y / PY / PN / N / NI
1.3. Were intervention discontinuations or switches likely to be related to factors that are prognostic for the outcome?<br>**If N/PN**, answer questions relating to baseline confounding (1.4 to 1.6)<br>**If Y/PY**, answer questions relating to both baseline and time-varying confounding (1.7 and 1.8)		NA / Y / PY / PN / N / NI

**Questions relating to baseline confounding only**		
1.4. Did the authors use an appropriate analysis method that controlled for all the important confounding domains?		NA / Y / PY / PN / N / NI
1.5. **If Y/PY to 1.4**: Were confounding domains that were controlled for measured validly and reliably by the variables available in this study?		NA / Y / PY / PN / N / NI
1.6. Did the authors control for any post-intervention variables that could have been affected by the intervention?		NA / Y / PY / PN / N / NI
**Questions relating to baseline and time-varying confounding**		
1.7. Did the authors use an appropriate analysis method that controlled for all the important confounding domains and for time-varying confounding?		NA / Y / PY / PN / N / NI
1.8. **If Y/PY to 1.7**: Were confounding domains that were controlled for measured validly and reliably by the variables available in this study?		NA / Y / PY / PN / N / NI
**Risk of bias judgement**		Low / Moderate / Serious / Critical / NI
Optional: What is the predicted direction of bias due to confounding?		Favours experimental / Favours comparator / Unpredictable

Bias in selection of participants into the study		
2.1. Was selection of participants into the study (or into the analysis) based on participant characteristics observed after the start of intervention?<br>**If N/PN to 2.1:** go to 2.4		Y / PY / PN / N / NI
2.2. **If Y/PY to 2.1**: Were the post-intervention variables that influenced selection likely to be associated with intervention?		NA / Y / PY / PN / N / NI
2.3 **If Y/PY to 2.2**: Were the post-intervention variables that influenced selection likely to be influenced by the outcome or a cause of the outcome?		NA / Y / PY / PN / N / NI
2.4. Do start of follow-up and start of intervention coincide for most participants?		Y / PY / PN / N / NI
2.5. **If Y/PY to 2.2 and 2.3, or N/PN to 2.4**: Were adjustment techniques used that are likely to correct for the presence of selection biases?		NA / Y / PY / PN / N / NI
**Risk of bias judgement**		Low / Moderate / Serious / Critical / NI
Optional: What is the predicted direction of bias due to selection of participants into the study?		Favours experimental / Favours comparator / Towards null /Away from null / Unpredictable

Bias in classification of interventions		
3.1 Were intervention groups clearly defined?		Y / PY / PN / N / NI
3.2 Was the information used to define intervention groups recorded at the start of the intervention?		Y / PY / PN / N / NI
3.3 Could classification of intervention status have been affected by knowledge of the outcome or risk of the outcome?		Y / PY / PN / N / NI
**Risk of bias judgement**		Low / Moderate / Serious / Critical / NI
Optional: What is the predicted direction of bias due to classification of interventions?		Favours experimental / Favours comparator / Towards null /Away from null / Unpredictable

Bias due to deviations from intended interventions		
**If your aim for this study is to assess the effect of assignment to intervention, answer questions 4.1 and 4.2**		
4.1. Were there deviations from the intended intervention beyond what would be expected in usual practice?		Y / PY / PN / N / NI
4.2. **If Y/PY to 4.1**: Were these deviations from intended intervention unbalanced between groups *and* likely to have affected the outcome?		NA / Y / PY / PN / N / NI
**If your aim for this study is to assess the effect of starting and adhering to intervention, answer questions 4.3 to 4.6**		
4.3. Were important co-interventions balanced across intervention groups?		Y / PY / PN / N / NI
4.4. Was the intervention implemented successfully for most participants?		Y / PY / PN / N / NI
4.5. Did study participants adhere to the assigned intervention regimen?		Y / PY / PN / N / NI
4.6. **If N/PN to 4.3, 4.4 or 4.5**: Was an appropriate analysis used to estimate the effect of starting and adhering to the intervention?		NA / Y / PY / PN / N / NI
**Risk of bias judgement**		Low / Moderate / Serious / Critical / NI
Optional: What is the predicted direction of bias due to deviations from the intended interventions?		Favours experimental / Favours comparator / Towards null /Away from null / Unpredictable

Bias due to missing data		
5.1 Were outcome data available for all, or nearly all, participants?		Y / PY / PN / N / NI
5.2 Were participants excluded due to missing data on intervention status?		Y / PY / PN / N / NI
5.3 Were participants excluded due to missing data on other variables needed for the analysis?		Y / PY / PN / N / NI
5.4 **If PN/N to 5.1, or Y/PY to 5.2 or 5.3**: Are the proportion of participants and reasons for missing data similar across interventions?		NA / Y / PY / PN / N / NI
5.5 **If PN/N to 5.1, or Y/PY to 5.2 or 5.3**: Is there evidence that results were robust to the presence of missing data?		NA / Y / PY / PN / N / NI
**Risk of bias judgement**		Low / Moderate / Serious / Critical / NI
Optional: What is the predicted direction of bias due to missing data?		Favours experimental / Favours comparator / Towards null /Away from null / Unpredictable

Bias in measurement of outcomes		
6.1 Could the outcome measure have been influenced by knowledge of the intervention received?		Y / PY / PN / N / NI
6.2 Were outcome assessors aware of the intervention received by study participants?		Y / PY / PN / N / NI
6.3 Were the methods of outcome assessment comparable across intervention groups?		Y / PY / PN / N / NI
6.4 Were any systematic errors in measurement of the outcome related to intervention received?		Y / PY / PN / N / NI
**Risk of bias judgement**		Low / Moderate / Serious / Critical / NI
Optional: What is the predicted direction of bias due to measurement of outcomes?		Favours experimental / Favours comparator / Towards null /Away from null / Unpredictable

Bias in selection of the reported result		
Is the reported effect estimate likely to be selected, on the basis of the results, from...<br>7.1. ... multiple outcome *measurements* within the outcome domain?		Y / PY / PN / N / NI
7.2 ... multiple *analyses* of the intervention-outcome relationship?		Y / PY / PN / N / NI
7.3 ... different *subgroups*?		Y / PY / PN / N / NI
**Risk of bias judgement**		Low / Moderate / Serious / Critical / NI
Optional: What is the predicted direction of bias due to selection of the reported result?		Favours experimental / Favours comparator / Towards null /Away from null / Unpredictable

Overall bias		
**Risk of bias judgement**		Low / Moderate / Serious / Critical / NI
Optional: What is the overall predicted direction of bias for this outcome?		Favours experimental / Favours comparator / Towards null /Away from null / Unpredictable

This work is licensed under a Creative Commons Attribution-NonCommercial-NoDerivatives 4.0 International License.

**Domain**	**Residual**
Bias due to confounding	Serious risk
Bias in selection of participants into the study	Low risk
Bias in classification of interventions	Low risk
Bias due to deviations from intended interventions	Low risk
Bias due to missing data	Low risk
Bias in measurement of outcomes	Low risk
Bias in selection of the reported result	Moderate risk
**Overall**	**Serious risk**

Dostalek 2023

Specify a target randomized trial specific to the study

Design	Individually randomized / Cluster randomized / Matched (e.g. cross-over)
Participants	Patients with histopathological diagnosis of AIS after cervical excision
Experimental intervention	Conservative treatment
Comparator	Demolitive treatment (hysterectomy)

Is your aim for this study...?

■ to assess the effect of *assignment to* intervention

☐ to assess the effect of *starting and adhering to* intervention

Specify the outcome

Specify which outcome is being assessed for risk of bias (typically from among those earmarked for the Summary of Findings table). Specify whether this is a proposed benefit or harm of intervention.

Recurrence<br>Residual

Specify the numerical result being assessed

In case of multiple alternative analyses being presented, specify the numeric result (e.g. RR = 1.52 (95% CI 0.83 to 2.77) and/or a reference (e.g. to a table, figure or paragraph) that uniquely defines the result being assessed.

Preliminary consideration of confounders

Complete a row for each important confounding domain (i) listed in the review protocol; and (ii) relevant to the setting of this particular study, or which the study authors identified as potentially important.

"Important" confounding domains are those for which, in the context of this study, adjustment is expected to lead to a clinically important change in the estimated effect of the intervention. "Validity" refers to whether the confounding variable or variables fully measure the domain, while "reliability" refers to the precision of the measurement (more measurement error means less reliability).

**(i) Confounding domains listed in the review protocol**				
Confounding domain	Measured variable(s)	Is there evidence that controlling for this variable was unnecessary?*	Is the confounding domain measured validly and reliably by this variable (or these variables)?	OPTIONAL: Is failure to adjust for this variable (alone) expected to favour the experimental intervention or the comparator?
Age	Years (>40 years, >35 years, >30 years)	No	Yes	-

**(ii) Additional confounding domains relevant to the setting of this particular study, or which the study authors identified as important**				
Confounding domain	Measured variable(s)	Is there evidence that controlling for this variable was unnecessary?*	Is the confounding domain measured validly and reliably by this variable (or these variables)?	OPTIONAL: Is failure to adjust for this variable (alone) expected to favour the experimental intervention or the comparator?
HPV positivity during follow-up	Positive/Negative	No	Yes	-
HPV typing	16/18 - non 16/18	No	Yes	-
Presence of concomitant squamous lesions	Yes/No	No	Yes	-
Repeated cone biopsy	Yes/No	No	Yes	-

* In the context of a particular study, variables can be demonstrated not to be confounders and so not included in the analysis: (a) if they are not predictive of the outcome; (b) if they are not predictive of intervention; or (c) because adjustment makes no or minimal difference to the estimated effect of the primary parameter. Note that "no statistically significant association" is not the same as "not predictive".

Preliminary consideration of co-interventions

Complete a row for each important co-intervention (i) listed in the review protocol; and (ii) relevant to the setting of this particular study, or which the study authors identified as important.

"Important" co-interventions are those for which, in the context of this study, adjustment is expected to lead to a clinically important change in the estimated effect of the intervention.

**(i) Co-interventions listed in the review protocol**		
Co-intervention	Is there evidence that controlling for this co-intervention was unnecessary (e.g. because it was not administered)?	Is presence of this co-intervention likely to favour outcomes in the experimental intervention or the comparator
None	-	-

**(ii) Additional co-interventions relevant to the setting of this particular study, or which the study authors identified as important**		
Co-intervention	Is there evidence that controlling for this co-intervention was unnecessary (e.g. because it was not administered)?	Is presence of this co-intervention likely to favour outcomes in the experimental intervention or the comparator
None	-	-

Risk of bias assessment

Responses underlined in green are potential markers for low risk of bias, and responses in red are potential markers for a risk of bias. Where questions relate only to sign posts to other questions, no formatting is used.

Signalling questions	Description	Response options
**Bias due to confounding**		
1.1 Is there potential for confounding of the effect of intervention in this study?<br>**If N/PN to 1.1:** the study can be considered to be at low risk of bias due to confounding and no further signalling questions need be considered		Y / PY / PN / N
**If Y/PY to 1.1**: determine whether there is a need to assess time-varying confounding:		
1.2. Was the analysis based on splitting participants' follow up time according to intervention received?<br>**If N/PN**, answer questions relating to baseline confounding (1.4 to 1.6)<br>**If Y/PY**, go to question 1.3.		NA / Y / PY / PN / N / NI
1.3. Were intervention discontinuations or switches likely to be related to factors that are prognostic for the outcome?<br>**If N/PN**, answer questions relating to baseline confounding (1.4 to 1.6)<br>**If Y/PY**, answer questions relating to both baseline and time-varying confounding (1.7 and 1.8)		NA / Y / PY / PN / N / NI

**Questions relating to baseline confounding only**		
1.4. Did the authors use an appropriate analysis method that controlled for all the important confounding domains?		NA / Y / PY / PN / N / NI
1.5. **If Y/PY to 1.4**: Were confounding domains that were controlled for measured validly and reliably by the variables available in this study?		NA / Y / PY / PN / N / NI
1.6. Did the authors control for any post-intervention variables that could have been affected by the intervention?		NA / Y / PY / PN / N / NI
**Questions relating to baseline and time-varying confounding**		
1.7. Did the authors use an appropriate analysis method that controlled for all the important confounding domains and for time-varying confounding?		NA / Y / PY / PN / N / NI
1.8. **If Y/PY to 1.7**: Were confounding domains that were controlled for measured validly and reliably by the variables available in this study?		NA / Y / PY / PN / N / NI
**Risk of bias judgement**		Low / Moderate / Serious / Critical / NI
Optional: What is the predicted direction of bias due to confounding?		Favours experimental / Favours comparator / Unpredictable

Bias in selection of participants into the study		
2.1. Was selection of participants into the study (or into the analysis) based on participant characteristics observed after the start of intervention?<br>**If N/PN to 2.1:** go to 2.4		Y / PY / PN / N / NI
2.2. **If Y/PY to 2.1**: Were the post-intervention variables that influenced selection likely to be associated with intervention?		NA / Y / PY / PN / N / NI
2.3 **If Y/PY to 2.2**: Were the post-intervention variables that influenced selection likely to be influenced by the outcome or a cause of the outcome?		NA / Y / PY / PN / N / NI
2.4. Do start of follow-up and start of intervention coincide for most participants?		Y / PY / PN / N / NI
2.5. **If Y/PY to 2.2 and 2.3, or N/PN to 2.4**: Were adjustment techniques used that are likely to correct for the presence of selection biases?		NA / Y / PY / PN / N / NI
**Risk of bias judgement**		Low / Moderate / Serious / Critical / NI
Optional: What is the predicted direction of bias due to selection of participants into the study?		Favours experimental / Favours comparator / Towards null /Away from null / Unpredictable

Bias in classification of interventions		
3.1 Were intervention groups clearly defined?		Y / PY / PN / N / NI
3.2 Was the information used to define intervention groups recorded at the start of the intervention?		Y / PY / PN / N / NI
3.3 Could classification of intervention status have been affected by knowledge of the outcome or risk of the outcome?		Y / PY / PN / N / NI
**Risk of bias judgement**		Low / Moderate / Serious / Critical / NI
Optional: What is the predicted direction of bias due to classification of interventions?		Favours experimental / Favours comparator / Towards null /Away from null / Unpredictable

Bias due to deviations from intended interventions		
**If your aim for this study is to assess the effect of assignment to intervention, answer questions 4.1 and 4.2**		
4.1. Were there deviations from the intended intervention beyond what would be expected in usual practice?		Y / PY / PN / N / NI
4.2. **If Y/PY to 4.1**: Were these deviations from intended intervention unbalanced between groups *and* likely to have affected the outcome?		NA / Y / PY / PN / N / NI
**If your aim for this study is to assess the effect of starting and adhering to intervention, answer questions 4.3 to 4.6**		
4.3. Were important co-interventions balanced across intervention groups?		Y / PY / PN / N / NI
4.4. Was the intervention implemented successfully for most participants?		Y / PY / PN / N / NI
4.5. Did study participants adhere to the assigned intervention regimen?		Y / PY / PN / N / NI
4.6. **If N/PN to 4.3, 4.4 or 4.5**: Was an appropriate analysis used to estimate the effect of starting and adhering to the intervention?		NA / Y / PY / PN / N / NI
**Risk of bias judgement**		Low / Moderate / Serious / Critical / NI
Optional: What is the predicted direction of bias due to deviations from the intended interventions?		Favours experimental / Favours comparator / Towards null /Away from null / Unpredictable

Bias due to missing data		
5.1 Were outcome data available for all, or nearly all, participants?		Y / PY / PN / N / NI
5.2 Were participants excluded due to missing data on intervention status?		Y / PY / PN / N / NI
5.3 Were participants excluded due to missing data on other variables needed for the analysis?		Y / PY / PN / N / NI
5.4 **If PN/N to 5.1, or Y/PY to 5.2 or 5.3**: Are the proportion of participants and reasons for missing data similar across interventions?		NA / Y / PY / PN / N / NI
5.5 **If PN/N to 5.1, or Y/PY to 5.2 or 5.3**: Is there evidence that results were robust to the presence of missing data?		NA / Y / PY / PN / N / NI
**Risk of bias judgement**		Low / Moderate / Serious / Critical / NI
Optional: What is the predicted direction of bias due to missing data?		Favours experimental / Favours comparator / Towards null /Away from null / Unpredictable

Bias in measurement of outcomes		
6.1 Could the outcome measure have been influenced by knowledge of the intervention received?		Y / PY / PN / N / NI
6.2 Were outcome assessors aware of the intervention received by study participants?		Y / PY / PN / N / NI
6.3 Were the methods of outcome assessment comparable across intervention groups?		Y / PY / PN / N / NI
6.4 Were any systematic errors in measurement of the outcome related to intervention received?		Y / PY / PN / N / NI
**Risk of bias judgement**		Low / Moderate / Serious / Critical / NI
Optional: What is the predicted direction of bias due to measurement of outcomes?		Favours experimental / Favours comparator / Towards null /Away from null / Unpredictable

Bias in selection of the reported result		
Is the reported effect estimate likely to be selected, on the basis of the results, from...<br>7.1. ... multiple outcome *measurements* within the outcome domain?		Y / PY / PN / N / NI
7.2 ... multiple *analyses* of the intervention-outcome relationship?		Y / PY / PN / N / NI
7.3 ... different *subgroups*?		Y / PY / PN / N / NI
**Risk of bias judgement**		Low / Moderate / Serious / Critical / NI
Optional: What is the predicted direction of bias due to selection of the reported result?		Favours experimental / Favours comparator / Towards null /Away from null / Unpredictable

Overall bias		
**Risk of bias judgement**		Low / Moderate (recurrence) / Serious (residual) / Critical / NI
Optional: What is the overall predicted direction of bias for this outcome?		Favours experimental / Favours comparator / Towards null /Away from null / Unpredictable

This work is licensed under a Creative Commons Attribution-NonCommercial-NoDerivatives 4.0 International License.

Domain	Recurrence
Bias due to confounding	Moderate risk
Bias in selection of participants into the study	Low risk
Bias in classification of interventions	Low risk
Bias due to deviations from intended interventions	Low risk
Bias due to missing data	Low risk
Bias in measurement of outcomes	Low risk
Bias in selection of the reported result	Moderate risk
**Overall**	**Moderate risk**

Giannella 2022

Specify a target randomized trial specific to the study

Design	Individually randomized / Cluster randomized / Matched (e.g. cross-over)
Participants	Patients with histopathological diagnosis of AIS after cervical excision
Experimental intervention	Conservative treatment
Comparator	Demolitive treatment (hysterectomy)

Is your aim for this study…?

- ■ to assess the effect of *assignment to* intervention
- ☐ to assess the effect of *starting and adhering to* intervention

Specify the outcome

Specify which outcome is being assessed for risk of bias (typically from among those earmarked for the Summary of Findings table). Specify whether this is a proposed benefit or harm of intervention.

Recurrence

Specify the numerical result being assessed

In case of multiple alternative analyses being presented, specify the numeric result (e.g. RR = 1.52 (95% CI 0.83 to 2.77) and/or a reference (e.g. to a table, figure or paragraph) that uniquely defines the result being assessed.

Preliminary consideration of confounders

Complete a row for each important confounding domain (i) listed in the review protocol; and (ii) relevant to the setting of this particular study, or which the study authors identified as potentially important.

"Important" confounding domains are those for which, in the context of this study, adjustment is expected to lead to a clinically important change in the estimated effect of the intervention. "Validity" refers to whether the confounding variable or variables fully measure the domain, while "reliability" refers to the precision of the measurement (more measurement error means less reliability).

**(i) Confounding domains listed in the review protocol**				
Confounding domain	Measured variable(s)	Is there evidence that controlling for this variable was unnecessary?*	Is the confounding domain measured validly and reliably by this variable (or these variables)?	OPTIONAL: Is failure to adjust for this variable (alone) expected to favour the experimental intervention or the comparator?
Age	Not evaluated as confounder	No	-	-

**(ii) Additional confounding domains relevant to the setting of this particular study, or which the study authors identified as important**				
Confounding domain	Measured variable(s)	Is there evidence that controlling for this variable was unnecessary?*	Is the confounding domain measured validly and reliably by this variable (or these variables)?	OPTIONAL: Is failure to adjust for this variable (alone) expected to favour the experimental intervention or the comparator?
-	-	-	-	-

* In the context of a particular study, variables can be demonstrated not to be confounders and so not included in the analysis: (a) if they are not predictive of the outcome; (b) if they are not predictive of intervention; or (c) because adjustment makes no or minimal difference to the estimated effect of the primary parameter. Note that "no statistically significant association" is not the same as "not predictive".

Preliminary consideration of co-interventions

Complete a row for each important co-intervention (i) listed in the review protocol; and (ii) relevant to the setting of this particular study, or which the study authors identified as important.

"Important" co-interventions are those for which, in the context of this study, adjustment is expected to lead to a clinically important change in the estimated effect of the intervention.

**(i) Co-interventions listed in the review protocol**		
Co-intervention	Is there evidence that controlling for this co-intervention was unnecessary (e.g. because it was not administered)?	Is presence of this co-intervention likely to favour outcomes in the experimental intervention or the comparator
None	-	-

**(ii) Additional co-interventions relevant to the setting of this particular study, or which the study authors identified as important**		
Co-intervention	Is there evidence that controlling for this co-intervention was unnecessary (e.g. because it was not administered)?	Is presence of this co-intervention likely to favour outcomes in the experimental intervention or the comparator
None	-	-

Risk of bias assessment

Responses underlined in green are potential markers for low risk of bias, and responses in red are potential markers for a risk of bias. Where questions relate only to sign posts to other questions, no formatting is used.

Signalling questions	Description	Response options
**Bias due to confounding**		
1.1 Is there potential for confounding of the effect of intervention in this study?<br>**If N/PN to 1.1:** the study can be considered to be at low risk of bias due to confounding and no further signalling questions need be considered		Y / PY / PN / N
**If Y/PY to 1.1**: determine whether there is a need to assess time-varying confounding:		
1.2. Was the analysis based on splitting participants' follow up time according to intervention received?<br>**If N/PN**, answer questions relating to baseline confounding (1.4 to 1.6)<br>**If Y/PY**, go to question 1.3.		NA / Y / PY / PN / N / NI
1.3. Were intervention discontinuations or switches likely to be related to factors that are prognostic for the outcome?<br>**If N/PN**, answer questions relating to baseline confounding (1.4 to 1.6)<br>**If Y/PY**, answer questions relating to both baseline and time-varying confounding (1.7 and 1.8)		NA / Y / PY / PN / N / NI

**Questions relating to baseline confounding only**		
1.4. Did the authors use an appropriate analysis method that controlled for all the important confounding domains?		NA / Y / PY / PN / N / NI
1.5. **If Y/PY to 1.4**: Were confounding domains that were controlled for measured validly and reliably by the variables available in this study?		NA / Y / PY / PN / N / NI
1.6. Did the authors control for any post-intervention variables that could have been affected by the intervention?		NA / Y / PY / PN / N / NI
**Questions relating to baseline and time-varying confounding**		
1.7. Did the authors use an appropriate analysis method that controlled for all the important confounding domains and for time-varying confounding?		NA / Y / PY / PN / N / NI
1.8. **If Y/PY to 1.7**: Were confounding domains that were controlled for measured validly and reliably by the variables available in this study?		NA / Y / PY / PN / N / NI
**Risk of bias judgement**		Low / Moderate / Serious / Critical / NI
Optional: What is the predicted direction of bias due to confounding?		Favours experimental / Favours comparator / Unpredictable

Bias in selection of participants into the study		
2.1. Was selection of participants into the study (or into the analysis) based on participant characteristics observed after the start of intervention?<br>**If N/PN to 2.1:** go to 2.4		Y / PY / PN / N / NI
2.2. **If Y/PY to 2.1**: Were the post-intervention variables that influenced selection likely to be associated with intervention?		NA / Y / PY / PN / N / NI
2.3 **If Y/PY to 2.2**: Were the post-intervention variables that influenced selection likely to be influenced by the outcome or a cause of the outcome?		NA / Y / PY / PN / N / NI
2.4. Do start of follow-up and start of intervention coincide for most participants?		Y / PY / PN / N / NI
2.5. **If Y/PY to 2.2 and 2.3, or N/PN to 2.4**: Were adjustment techniques used that are likely to correct for the presence of selection biases?		NA / Y / PY / PN / N / NI
**Risk of bias judgement**		Low / Moderate / Serious / Critical / NI
Optional: What is the predicted direction of bias due to selection of participants into the study?		Favours experimental / Favours comparator / Towards null /Away from null / Unpredictable

Bias in classification of interventions		
3.1 Were intervention groups clearly defined?		Y / PY / PN / N / NI
3.2 Was the information used to define intervention groups recorded at the start of the intervention?		Y / PY / PN / N / NI
3.3 Could classification of intervention status have been affected by knowledge of the outcome or risk of the outcome?		Y / PY / PN / N / NI
**Risk of bias judgement**		Low / Moderate / Serious / Critical / NI
Optional: What is the predicted direction of bias due to classification of interventions?		Favours experimental / Favours comparator / Towards null /Away from null / Unpredictable

Bias due to deviations from intended interventions		
**If your aim for this study is to assess the effect of assignment to intervention, answer questions 4.1 and 4.2**		
4.1. Were there deviations from the intended intervention beyond what would be expected in usual practice?		Y / PY / PN / N / NI
4.2. **If Y/PY to 4.1**: Were these deviations from intended intervention unbalanced between groups *and* likely to have affected the outcome?		NA / Y / PY / PN / N / NI
**If your aim for this study is to assess the effect of starting and adhering to intervention, answer questions 4.3 to 4.6**		
4.3. Were important co-interventions balanced across intervention groups?		Y / PY / PN / N / NI
4.4. Was the intervention implemented successfully for most participants?		Y / PY / PN / N / NI
4.5. Did study participants adhere to the assigned intervention regimen?		Y / PY / PN / N / NI
4.6. **If N/PN to 4.3, 4.4 or 4.5**: Was an appropriate analysis used to estimate the effect of starting and adhering to the intervention?		NA / Y / PY / PN / N / NI
**Risk of bias judgement**		Low / Moderate / Serious / Critical / NI
Optional: What is the predicted direction of bias due to deviations from the intended interventions?		Favours experimental / Favours comparator / Towards null /Away from null / Unpredictable

Bias due to missing data		
5.1 Were outcome data available for all, or nearly all, participants?		Y / PY / PN / N / NI
5.2 Were participants excluded due to missing data on intervention status?		Y / PY / PN / N / NI
5.3 Were participants excluded due to missing data on other variables needed for the analysis?		Y / PY / PN / N / NI
5.4 **If PN/N to 5.1, or Y/PY to 5.2 or 5.3**: Are the proportion of participants and reasons for missing data similar across interventions?		NA / Y / PY / PN / N / NI
5.5 **If PN/N to 5.1, or Y/PY to 5.2 or 5.3**: Is there evidence that results were robust to the presence of missing data?		NA / Y / PY / PN / N / NI
**Risk of bias judgement**		Low / Moderate / Serious / Critical / NI
Optional: What is the predicted direction of bias due to missing data?		Favours experimental / Favours comparator / Towards null /Away from null / Unpredictable

Bias in measurement of outcomes		
6.1 Could the outcome measure have been influenced by knowledge of the intervention received?		Y / PY / PN / N / NI
6.2 Were outcome assessors aware of the intervention received by study participants?		Y / PY / PN / N / NI
6.3 Were the methods of outcome assessment comparable across intervention groups?		Y / PY / PN / N / NI
6.4 Were any systematic errors in measurement of the outcome related to intervention received?		Y / PY / PN / N / NI
**Risk of bias judgement**		Low / Moderate / Serious / Critical / NI
Optional: What is the predicted direction of bias due to measurement of outcomes?		Favours experimental / Favours comparator / Towards null /Away from null / Unpredictable

Bias in selection of the reported result		
Is the reported effect estimate likely to be selected, on the basis of the results, from...<br>7.1. ... multiple outcome *measurements* within the outcome domain?		Y / PY / PN / N / NI
7.2 ... multiple *analyses* of the intervention-outcome relationship?		Y / PY / PN / N / NI
7.3 ... different *subgroups*?		Y / PY / PN / N / NI
**Risk of bias judgement**		Low / Moderate / Serious / Critical / NI
Optional: What is the predicted direction of bias due to selection of the reported result?		Favours experimental / Favours comparator / Towards null /Away from null / Unpredictable

Overall bias		
**Risk of bias judgement**		Low / Moderate / Serious / Critical / NI
Optional: What is the overall predicted direction of bias for this outcome?		Favours experimental / Favours comparator / Towards null /Away from null / Unpredictable

This work is licensed under a Creative Commons Attribution-NonCommercial-NoDerivatives 4.0 International License.

**Domain**	**Recurrence**
Bias due to confounding	Serious risk
Bias in selection of participants into the study	Low risk
Bias in classification of interventions	Low risk
Bias due to deviations from intended interventions	Low risk
Bias due to missing data	Low risk
Bias in measurement of outcomes	Low risk
Bias in selection of the reported result	Moderate risk
**Overall**	**Serious risk**

Hwang 2004

Specify a target randomized trial specific to the study

Design	Individually randomized / Cluster randomized / Matched (e.g. cross-over)
Participants	Patients with histopathological diagnosis of AIS after cervical excision
Experimental intervention	Conservative treatment
Comparator	Demolitive treatment (hysterectomy)

Is your aim for this study…?

- ■ to assess the effect of *assignment to* intervention
- ☐ to assess the effect of *starting and adhering to* intervention

Specify the outcome

Specify which outcome is being assessed for risk of bias (typically from among those earmarked for the Summary of Findings table). Specify whether this is a proposed benefit or harm of intervention.

Recurrence<br>Residual

Specify the numerical result being assessed

In case of multiple alternative analyses being presented, specify the numeric result (e.g. RR = 1.52 (95% CI 0.83 to 2.77) and/or a reference (e.g. to a table, figure or paragraph) that uniquely defines the result being assessed.

Preliminary consideration of confounders

Complete a row for each important confounding domain (i) listed in the review protocol; and (ii) relevant to the setting of this particular study, or which the study authors identified as potentially important.

"Important" confounding domains are those for which, in the context of this study, adjustment is expected to lead to a clinically important change in the estimated effect of the intervention. "Validity" refers to whether the confounding variable or variables fully measure the domain, while "reliability" refers to the precision of the measurement (more measurement error means less reliability).

**(i) Confounding domains listed in the review protocol**				
Confounding domain	Measured variable(s)	Is there evidence that controlling for this variable was unnecessary?*	Is the confounding domain measured validly and reliably by this variable (or these variables)?	OPTIONAL: Is failure to adjust for this variable (alone) expected to favour the experimental intervention or the comparator?
Age	Not evaluated as confounder	No	-	-

**(ii) Additional confounding domains relevant to the setting of this particular study, or which the study authors identified as important**				
Confounding domain	Measured variable(s)	Is there evidence that controlling for this variable was unnecessary?*	Is the confounding domain measured validly and reliably by this variable (or these variables)?	OPTIONAL: Is failure to adjust for this variable (alone) expected to favour the experimental intervention or the comparator?
-	-	-	-	-

* In the context of a particular study, variables can be demonstrated not to be confounders and so not included in the analysis: (a) if they are not predictive of the outcome; (b) if they are not predictive of intervention; or (c) because adjustment makes no or minimal difference to the estimated effect of the primary parameter. Note that "no statistically significant association" is not the same as "not predictive".

Preliminary consideration of co-interventions

Complete a row for each important co-intervention (i) listed in the review protocol; and (ii) relevant to the setting of this particular study, or which the study authors identified as important.

"Important" co-interventions are those for which, in the context of this study, adjustment is expected to lead to a clinically important change in the estimated effect of the intervention.

**(i) Co-interventions listed in the review protocol**		
Co-intervention	Is there evidence that controlling for this co-intervention was unnecessary (e.g. because it was not administered)?	Is presence of this co-intervention likely to favour outcomes in the experimental intervention or the comparator
None	-	-

**(ii) Additional co-interventions relevant to the setting of this particular study, or which the study authors identified as important**		
Co-intervention	Is there evidence that controlling for this co-intervention was unnecessary (e.g. because it was not administered)?	Is presence of this co-intervention likely to favour outcomes in the experimental intervention or the comparator
None	-	-

Risk of bias assessment

Responses underlined in green are potential markers for low risk of bias, and responses in red are potential markers for a risk of bias. Where questions relate only to sign posts to other questions, no formatting is used.

Signalling questions	Description	Response options
**Bias due to confounding**		
1.1 Is there potential for confounding of the effect of intervention in this study?<br>**If N/PN to 1.1:** the study can be considered to be at low risk of bias due to confounding and no further signalling questions need be considered		Y / PY / PN / N
**If Y/PY to 1.1**: determine whether there is a need to assess time-varying confounding:		
1.2. Was the analysis based on splitting participants' follow up time according to intervention received?<br>**If N/PN**, answer questions relating to baseline confounding (1.4 to 1.6)<br>**If Y/PY**, go to question 1.3.		NA / Y / PY / PN / N / NI
1.3. Were intervention discontinuations or switches likely to be related to factors that are prognostic for the outcome?<br>**If N/PN**, answer questions relating to baseline confounding (1.4 to 1.6)<br>**If Y/PY**, answer questions relating to both baseline and time-varying confounding (1.7 and 1.8)		NA / Y / PY / PN / N / NI

**Questions relating to baseline confounding only**		
1.4. Did the authors use an appropriate analysis method that controlled for all the important confounding domains?		NA / Y / PY / PN / N / NI
1.5. **If Y/PY to 1.4**: Were confounding domains that were controlled for measured validly and reliably by the variables available in this study?		NA / Y / PY / PN / N / NI
1.6. Did the authors control for any post-intervention variables that could have been affected by the intervention?		NA / Y / PY / PN / N / NI
**Questions relating to baseline and time-varying confounding**		
1.7. Did the authors use an appropriate analysis method that controlled for all the important confounding domains and for time-varying confounding?		NA / Y / PY / PN / N / NI
1.8. **If Y/PY to 1.7**: Were confounding domains that were controlled for measured validly and reliably by the variables available in this study?		NA / Y / PY / PN / N / NI
**Risk of bias judgement**		Low / Moderate / Serious / Critical / NI
Optional: What is the predicted direction of bias due to confounding?		Favours experimental / Favours comparator / Unpredictable

Bias in selection of participants into the study		
2.1. Was selection of participants into the study (or into the analysis) based on participant characteristics observed after the start of intervention?<br>**If N/PN to 2.1:** go to 2.4		Y / PY / PN / N / NI
2.2. **If Y/PY to 2.1**: Were the post-intervention variables that influenced selection likely to be associated with intervention?		NA / Y / PY / PN / N / NI
2.3 **If Y/PY to 2.2**: Were the post-intervention variables that influenced selection likely to be influenced by the outcome or a cause of the outcome?		NA / Y / PY / PN / N / NI
2.4. Do start of follow-up and start of intervention coincide for most participants?		Y / PY / PN / N / NI
2.5. **If Y/PY to 2.2 and 2.3, or N/PN to 2.4**: Were adjustment techniques used that are likely to correct for the presence of selection biases?		NA / Y / PY / PN / N / NI
**Risk of bias judgement**		Low / Moderate / Serious / Critical / NI
Optional: What is the predicted direction of bias due to selection of participants into the study?		Favours experimental / Favours comparator / Towards null /Away from null / Unpredictable

Bias in classification of interventions		
3.1 Were intervention groups clearly defined?		Y / PY / PN / N / NI
3.2 Was the information used to define intervention groups recorded at the start of the intervention?		Y / PY / PN / N / NI
3.3 Could classification of intervention status have been affected by knowledge of the outcome or risk of the outcome?		Y / PY / PN / N / NI
**Risk of bias judgement**		Low / Moderate / Serious / Critical / NI
Optional: What is the predicted direction of bias due to classification of interventions?		Favours experimental / Favours comparator / Towards null /Away from null / Unpredictable

Bias due to deviations from intended interventions		
**If your aim for this study is to assess the effect of assignment to intervention, answer questions 4.1 and 4.2**		
4.1. Were there deviations from the intended intervention beyond what would be expected in usual practice?		Y / PY / PN / N / NI
4.2. **If Y/PY to 4.1**: Were these deviations from intended intervention unbalanced between groups *and* likely to have affected the outcome?		NA / Y / PY / PN / N / NI
**If your aim for this study is to assess the effect of starting and adhering to intervention, answer questions 4.3 to 4.6**		
4.3. Were important co-interventions balanced across intervention groups?		Y / PY / PN / N / NI
4.4. Was the intervention implemented successfully for most participants?		Y / PY / PN / N / NI
4.5. Did study participants adhere to the assigned intervention regimen?		Y / PY / PN / N / NI
4.6. **If N/PN to 4.3, 4.4 or 4.5**: Was an appropriate analysis used to estimate the effect of starting and adhering to the intervention?		NA / Y / PY / PN / N / NI
**Risk of bias judgement**		Low / Moderate / Serious / Critical / NI
Optional: What is the predicted direction of bias due to deviations from the intended interventions?		Favours experimental / Favours comparator / Towards null /Away from null / Unpredictable

Bias due to missing data		
5.1 Were outcome data available for all, or nearly all, participants?		Y / PY / PN / N / NI
5.2 Were participants excluded due to missing data on intervention status?		Y / PY / PN / N / NI
5.3 Were participants excluded due to missing data on other variables needed for the analysis?		Y / PY / PN / N / NI
5.4 **If PN/N to 5.1, or Y/PY to 5.2 or 5.3**: Are the proportion of participants and reasons for missing data similar across interventions?		NA / Y / PY / PN / N / NI
5.5 **If PN/N to 5.1, or Y/PY to 5.2 or 5.3**: Is there evidence that results were robust to the presence of missing data?		NA / Y / PY / PN / N / NI
**Risk of bias judgement**		Low / Moderate / Serious / Critical / NI
Optional: What is the predicted direction of bias due to missing data?		Favours experimental / Favours comparator / Towards null /Away from null / Unpredictable

Bias in measurement of outcomes		
6.1 Could the outcome measure have been influenced by knowledge of the intervention received?		Y / PY / PN / N / NI
6.2 Were outcome assessors aware of the intervention received by study participants?		Y / PY / PN / N / NI
6.3 Were the methods of outcome assessment comparable across intervention groups?		Y / PY / PN / N / NI
6.4 Were any systematic errors in measurement of the outcome related to intervention received?		Y / PY / PN / N / NI
**Risk of bias judgement**		Low / Moderate / Serious / Critical / NI
Optional: What is the predicted direction of bias due to measurement of outcomes?		Favours experimental / Favours comparator / Towards null /Away from null / Unpredictable

Bias in selection of the reported result		
Is the reported effect estimate likely to be selected, on the basis of the results, from...<br>7.1. ... multiple outcome *measurements* within the outcome domain?		Y / PY / PN / N / NI
7.2 ... multiple *analyses* of the intervention-outcome relationship?		Y / PY / PN / N / NI
7.3 ... different *subgroups*?		Y / PY / PN / N / NI
**Risk of bias judgement**		Low / Moderate / Serious / Critical / NI
Optional: What is the predicted direction of bias due to selection of the reported result?		Favours experimental / Favours comparator / Towards null /Away from null / Unpredictable

Overall bias		
**Risk of bias judgement**		Low / Moderate / Serious / Critical / NI
Optional: What is the overall predicted direction of bias for this outcome?		Favours experimental / Favours comparator / Towards null /Away from null / Unpredictable

This work is licensed under a Creative Commons Attribution-NonCommercial-NoDerivatives 4.0 International License.

**Domain**	**Recurrence**	**Residual**
Bias due to confounding	Serious risk	Serious risk
Bias in selection of participants into the study	Low risk	Low risk
Bias in classification of interventions	Low risk	Low risk
Bias due to deviations from intended interventions	Low risk	Low risk
Bias due to missing data	Low risk	Low risk
Bias in measurement of outcomes	Low risk	Low risk
Bias in selection of the reported result	Moderate risk	Moderate risk
**Overall**	**Serious risk**	**Serious risk**

Im 1995

Specify a target randomized trial specific to the study

Design	Individually randomized / Cluster randomized / Matched (e.g. cross-over)
Participants	Patients with histopathological diagnosis of AIS after cervical excision
Experimental intervention	Conservative treatment
Comparator	Demolitive treatment (hysterectomy)

Is your aim for this study…?

- ■ to assess the effect of *assignment to* intervention
- ☐ to assess the effect of *starting and adhering to* intervention

Specify the outcome

Specify which outcome is being assessed for risk of bias (typically from among those earmarked for the Summary of Findings table). Specify whether this is a proposed benefit or harm of intervention.

Recurrence<br>Residual

Specify the numerical result being assessed

In case of multiple alternative analyses being presented, specify the numeric result (e.g. RR = 1.52 (95% CI 0.83 to 2.77) and/or a reference (e.g. to a table, figure or paragraph) that uniquely defines the result being assessed.

Preliminary consideration of confounders

Complete a row for each important confounding domain (i) listed in the review protocol; and (ii) relevant to the setting of this particular study, or which the study authors identified as potentially important.

"Important" confounding domains are those for which, in the context of this study, adjustment is expected to lead to a clinically important change in the estimated effect of the intervention. "Validity" refers to whether the confounding variable or variables fully measure the domain, while "reliability" refers to the precision of the measurement (more measurement error means less reliability).

**(i) Confounding domains listed in the review protocol**				
Confounding domain	Measured variable(s)	Is there evidence that controlling for this variable was unnecessary?*	Is the confounding domain measured validly and reliably by this variable (or these variables)?	OPTIONAL: Is failure to adjust for this variable (alone) expected to favour the experimental intervention or the comparator?
Age	Not evaluated as confounder	No	-	-

**(ii) Additional confounding domains relevant to the setting of this particular study, or which the study authors identified as important**				
Confounding domain	Measured variable(s)	Is there evidence that controlling for this variable was unnecessary?*	Is the confounding domain measured validly and reliably by this variable (or these variables)?	OPTIONAL: Is failure to adjust for this variable (alone) expected to favour the experimental intervention or the comparator?
-	-	-	-	-

* In the context of a particular study, variables can be demonstrated not to be confounders and so not included in the analysis: (a) if they are not predictive of the outcome; (b) if they are not predictive of intervention; or (c) because adjustment makes no or minimal difference to the estimated effect of the primary parameter. Note that "no statistically significant association" is not the same as "not predictive".

Preliminary consideration of co-interventions

Complete a row for each important co-intervention (i) listed in the review protocol; and (ii) relevant to the setting of this particular study, or which the study authors identified as important.

"Important" co-interventions are those for which, in the context of this study, adjustment is expected to lead to a clinically important change in the estimated effect of the intervention.

**(i) Co-interventions listed in the review protocol**		
Co-intervention	Is there evidence that controlling for this co-intervention was unnecessary (e.g. because it was not administered)?	Is presence of this co-intervention likely to favour outcomes in the experimental intervention or the comparator
None	-	-

**(ii) Additional co-interventions relevant to the setting of this particular study, or which the study authors identified as important**		
Co-intervention	Is there evidence that controlling for this co-intervention was unnecessary (e.g. because it was not administered)?	Is presence of this co-intervention likely to favour outcomes in the experimental intervention or the comparator
None	-	-

Risk of bias assessment

Responses underlined in green are potential markers for low risk of bias, and responses in red are potential markers for a risk of bias. Where questions relate only to sign posts to other questions, no formatting is used.

Signalling questions	Description	Response options
**Bias due to confounding**		
1.1 Is there potential for confounding of the effect of intervention in this study?<br>**If N/PN to 1.1:** the study can be considered to be at low risk of bias due to confounding and no further signalling questions need be considered		Y / PY / PN / N
**If Y/PY to 1.1**: determine whether there is a need to assess time-varying confounding:		
1.2. Was the analysis based on splitting participants' follow up time according to intervention received?<br>**If N/PN**, answer questions relating to baseline confounding (1.4 to 1.6)<br>**If Y/PY**, go to question 1.3.		NA / Y / PY / PN / N / NI
1.3. Were intervention discontinuations or switches likely to be related to factors that are prognostic for the outcome?<br>**If N/PN**, answer questions relating to baseline confounding (1.4 to 1.6)<br>**If Y/PY**, answer questions relating to both baseline and time-varying confounding (1.7 and 1.8)		NA / Y / PY / PN / N / NI

**Questions relating to baseline confounding only**		
1.4. Did the authors use an appropriate analysis method that controlled for all the important confounding domains?		NA / Y / PY / PN / N / NI
1.5. **If Y/PY to 1.4**: Were confounding domains that were controlled for measured validly and reliably by the variables available in this study?		NA / Y / PY / PN / N / NI
1.6. Did the authors control for any post-intervention variables that could have been affected by the intervention?		NA / Y / PY / PN / N / NI
**Questions relating to baseline and time-varying confounding**		
1.7. Did the authors use an appropriate analysis method that controlled for all the important confounding domains and for time-varying confounding?		NA / Y / PY / PN / N / NI
1.8. **If Y/PY to 1.7**: Were confounding domains that were controlled for measured validly and reliably by the variables available in this study?		NA / Y / PY / PN / N / NI
**Risk of bias judgement**		Low / Moderate / Serious / Critical / NI
Optional: What is the predicted direction of bias due to confounding?		Favours experimental / Favours comparator / Unpredictable

Bias in selection of participants into the study		
2.1. Was selection of participants into the study (or into the analysis) based on participant characteristics observed after the start of intervention?<br>**If N/PN to 2.1:** go to 2.4		Y / PY / PN / N / NI
2.2. **If Y/PY to 2.1**: Were the post-intervention variables that influenced selection likely to be associated with intervention?		NA / Y / PY / PN / N / NI
2.3 **If Y/PY to 2.2**: Were the post-intervention variables that influenced selection likely to be influenced by the outcome or a cause of the outcome?		NA / Y / PY / PN / N / NI
2.4. Do start of follow-up and start of intervention coincide for most participants?		Y / PY / PN / N / NI
2.5. **If Y/PY to 2.2 and 2.3, or N/PN to 2.4**: Were adjustment techniques used that are likely to correct for the presence of selection biases?		NA / Y / PY / PN / N / NI
**Risk of bias judgement**		Low / Moderate / Serious / Critical / NI
Optional: What is the predicted direction of bias due to selection of participants into the study?		Favours experimental / Favours comparator / Towards null /Away from null / Unpredictable

Bias in classification of interventions		
3.1 Were intervention groups clearly defined?		Y / PY / PN / N / NI
3.2 Was the information used to define intervention groups recorded at the start of the intervention?		Y / PY / PN / N / NI
3.3 Could classification of intervention status have been affected by knowledge of the outcome or risk of the outcome?		Y / PY / PN / N / NI
**Risk of bias judgement**		Low / Moderate / Serious / Critical / NI
Optional: What is the predicted direction of bias due to classification of interventions?		Favours experimental / Favours comparator / Towards null /Away from null / Unpredictable

Bias due to deviations from intended interventions		
**If your aim for this study is to assess the effect of assignment to intervention, answer questions 4.1 and 4.2**		
4.1. Were there deviations from the intended intervention beyond what would be expected in usual practice?		Y / PY / PN / N / NI
4.2. **If Y/PY to 4.1**: Were these deviations from intended intervention unbalanced between groups *and* likely to have affected the outcome?		NA / Y / PY / PN / N / NI
**If your aim for this study is to assess the effect of starting and adhering to intervention, answer questions 4.3 to 4.6**		
4.3. Were important co-interventions balanced across intervention groups?		Y / PY / PN / N / NI
4.4. Was the intervention implemented successfully for most participants?		Y / PY / PN / N / NI
4.5. Did study participants adhere to the assigned intervention regimen?		Y / PY / PN / N / NI
4.6. **If N/PN to 4.3, 4.4 or 4.5**: Was an appropriate analysis used to estimate the effect of starting and adhering to the intervention?		NA / Y / PY / PN / N / NI
**Risk of bias judgement**		Low / Moderate / Serious / Critical / NI
Optional: What is the predicted direction of bias due to deviations from the intended interventions?		Favours experimental / Favours comparator / Towards null /Away from null / Unpredictable

Bias due to missing data		
5.1 Were outcome data available for all, or nearly all, participants?		Y / PY / PN / N / NI
5.2 Were participants excluded due to missing data on intervention status?		Y / PY / PN / N / NI
5.3 Were participants excluded due to missing data on other variables needed for the analysis?		Y / PY / PN / N / NI
5.4 **If PN/N to 5.1, or Y/PY to 5.2 or 5.3**: Are the proportion of participants and reasons for missing data similar across interventions?		NA / Y / PY / PN / N / NI
5.5 **If PN/N to 5.1, or Y/PY to 5.2 or 5.3**: Is there evidence that results were robust to the presence of missing data?		NA / Y / PY / PN / N / NI
**Risk of bias judgement**		Low / Moderate / Serious / Critical / NI
Optional: What is the predicted direction of bias due to missing data?		Favours experimental / Favours comparator / Towards null /Away from null / Unpredictable

Bias in measurement of outcomes		
6.1 Could the outcome measure have been influenced by knowledge of the intervention received?		Y / PY / PN / N / NI
6.2 Were outcome assessors aware of the intervention received by study participants?		Y / PY / PN / N / NI
6.3 Were the methods of outcome assessment comparable across intervention groups?		Y / PY / PN / N / NI
6.4 Were any systematic errors in measurement of the outcome related to intervention received?		Y / PY / PN / N / NI
**Risk of bias judgement**		Low / Moderate / Serious / Critical / NI
Optional: What is the predicted direction of bias due to measurement of outcomes?		Favours experimental / Favours comparator / Towards null /Away from null / Unpredictable

Bias in selection of the reported result		
Is the reported effect estimate likely to be selected, on the basis of the results, from... 7.1. ... multiple outcome *measurements* within the outcome domain?		Y / PY / PN / N / NI
7.2 ... multiple *analyses* of the intervention-outcome relationship?		Y / PY / PN / N / NI
7.3 ... different *subgroups*?		Y / PY / PN / N / NI
**Risk of bias judgement**		Low / Moderate / Serious / Critical / NI
Optional: What is the predicted direction of bias due to selection of the reported result?		Favours experimental / Favours comparator / Towards null /Away from null / Unpredictable

Overall bias		
**Risk of bias judgement**		Low / Moderate / Serious / Critical / NI
Optional: What is the overall predicted direction of bias for this outcome?		Favours experimental / Favours comparator / Towards null /Away from null / Unpredictable

This work is licensed under a Creative Commons Attribution-NonCommercial-NoDerivatives 4.0 International License.

**Domain**	**Residual**
Bias due to confounding	Serious risk
Bias in selection of participants into the study	Low risk
Bias in classification of interventions	Low risk
Bias due to deviations from intended interventions	Low risk
Bias due to missing data	Low risk
Bias in measurement of outcomes	Low risk
Bias in selection of the reported result	Moderate risk
**Overall**	**Serious risk**

Li 2013

Specify a target randomized trial specific to the study

Design	Individually randomized / Cluster randomized / Matched (e.g. cross-over)
Participants	Patients with histopathological diagnosis of AIS after cervical excision
Experimental intervention	Conservative treatment
Comparator	Demolitive treatment (hysterectomy)

Is your aim for this study…?

- ■ to assess the effect of *assignment to* intervention
- ☐ to assess the effect of *starting and adhering to* intervention

Specify the outcome

Specify which outcome is being assessed for risk of bias (typically from among those earmarked for the Summary of Findings table). Specify whether this is a proposed benefit or harm of intervention.

Recurrence

Specify the numerical result being assessed

In case of multiple alternative analyses being presented, specify the numeric result (e.g. RR = 1.52 (95% CI 0.83 to 2.77) and/or a reference (e.g. to a table, figure or paragraph) that uniquely defines the result being assessed.

Preliminary consideration of confounders

Complete a row for each important confounding domain (i) listed in the review protocol; and (ii) relevant to the setting of this particular study, or which the study authors identified as potentially important.

"Important" confounding domains are those for which, in the context of this study, adjustment is expected to lead to a clinically important change in the estimated effect of the intervention. "Validity" refers to whether the confounding variable or variables fully measure the domain, while "reliability" refers to the precision of the measurement (more measurement error means less reliability).

**(i) Confounding domains listed in the review protocol**				
Confounding domain	Measured variable(s)	Is there evidence that controlling for this variable was unnecessary?*	Is the confounding domain measured validly and reliably by this variable (or these variables)?	OPTIONAL: Is failure to adjust for this variable (alone) expected to favour the experimental intervention or the comparator?
Age	Years	No	Yes	-

**(ii) Additional confounding domains relevant to the setting of this particular study, or which the study authors identified as important**				
Confounding domain	Measured variable(s)	Is there evidence that controlling for this variable was unnecessary?*	Is the confounding domain measured validly and reliably by this variable (or these variables)?	OPTIONAL: Is failure to adjust for this variable (alone) expected to favour the experimental intervention or the comparator?
-	-	-	-	-

* In the context of a particular study, variables can be demonstrated not to be confounders and so not included in the analysis: (a) if they are not predictive of the outcome; (b) if they are not predictive of intervention; or (c) because adjustment makes no or minimal difference to the estimated effect of the primary parameter. Note that "no statistically significant association" is not the same as "not predictive".

Preliminary consideration of co-interventions

Complete a row for each important co-intervention (i) listed in the review protocol; and (ii) relevant to the setting of this particular study, or which the study authors identified as important.

"Important" co-interventions are those for which, in the context of this study, adjustment is expected to lead to a clinically important change in the estimated effect of the intervention.

**(i) Co-interventions listed in the review protocol**		
Co-intervention	Is there evidence that controlling for this co-intervention was unnecessary (e.g. because it was not administered)?	Is presence of this co-intervention likely to favour outcomes in the experimental intervention or the comparator
None	-	-

**(ii) Additional co-interventions relevant to the setting of this particular study, or which the study authors identified as important**		
Co-intervention	Is there evidence that controlling for this co-intervention was unnecessary (e.g. because it was not administered)?	Is presence of this co-intervention likely to favour outcomes in the experimental intervention or the comparator
None	-	-

Risk of bias assessment

Responses underlined in green are potential markers for low risk of bias, and responses in red are potential markers for a risk of bias. Where questions relate only to sign posts to other questions, no formatting is used.

Signalling questions	Description	Response options
**Bias due to confounding**		
1.1 Is there potential for confounding of the effect of intervention in this study?<br>**If N/PN to 1.1:** the study can be considered to be at low risk of bias due to confounding and no further signalling questions need be considered		Y / PY / PN / N
**If Y/PY to 1.1**: determine whether there is a need to assess time-varying confounding:		
1.2. Was the analysis based on splitting participants' follow up time according to intervention received?<br>**If N/PN**, answer questions relating to baseline confounding (1.4 to 1.6)<br>**If Y/PY**, go to question 1.3.		NA / Y / PY / PN / N / NI
1.3. Were intervention discontinuations or switches likely to be related to factors that are prognostic for the outcome?<br>**If N/PN**, answer questions relating to baseline confounding (1.4 to 1.6)<br>**If Y/PY**, answer questions relating to both baseline and time-varying confounding (1.7 and 1.8)		NA / Y / PY / PN / N / NI

**Questions relating to baseline confounding only**		
1.4. Did the authors use an appropriate analysis method that controlled for all the important confounding domains?		NA / Y / PY / PN / N / NI
1.5. **If Y/PY to 1.4**: Were confounding domains that were controlled for measured validly and reliably by the variables available in this study?		NA / Y / PY / PN / N / NI
1.6. Did the authors control for any post-intervention variables that could have been affected by the intervention?		NA / Y / PY / PN / N / NI
**Questions relating to baseline and time-varying confounding**		
1.7. Did the authors use an appropriate analysis method that controlled for all the important confounding domains and for time-varying confounding?		NA / Y / PY / PN / N / NI
1.8. **If Y/PY to 1.7**: Were confounding domains that were controlled for measured validly and reliably by the variables available in this study?		NA / Y / PY / PN / N / NI
**Risk of bias judgement**		Low / Moderate / Serious / Critical / NI
Optional: What is the predicted direction of bias due to confounding?		Favours experimental / Favours comparator / Unpredictable

Bias in selection of participants into the study		
2.1. Was selection of participants into the study (or into the analysis) based on participant characteristics observed after the start of intervention?<br>**If N/PN to 2.1:** go to 2.4		Y / PY / PN / N / NI
2.2. **If Y/PY to 2.1**: Were the post-intervention variables that influenced selection likely to be associated with intervention?		NA / Y / PY / PN / N / NI
2.3 **If Y/PY to 2.2**: Were the post-intervention variables that influenced selection likely to be influenced by the outcome or a cause of the outcome?		NA / Y / PY / PN / N / NI
2.4. Do start of follow-up and start of intervention coincide for most participants?		Y / PY / PN / N / NI
2.5. **If Y/PY to 2.2 and 2.3, or N/PN to 2.4**: Were adjustment techniques used that are likely to correct for the presence of selection biases?		NA / Y / PY / PN / N / NI
**Risk of bias judgement**		Low / Moderate / Serious / Critical / NI
Optional: What is the predicted direction of bias due to selection of participants into the study?		Favours experimental / Favours comparator / Towards null /Away from null / Unpredictable

Bias in classification of interventions		
3.1 Were intervention groups clearly defined?		Y / PY / PN / N / NI
3.2 Was the information used to define intervention groups recorded at the start of the intervention?		Y / PY / PN / N / NI
3.3 Could classification of intervention status have been affected by knowledge of the outcome or risk of the outcome?		Y / PY / PN / N / NI
**Risk of bias judgement**		Low / Moderate / Serious / Critical / NI
Optional: What is the predicted direction of bias due to classification of interventions?		Favours experimental / Favours comparator / Towards null /Away from null / Unpredictable

Bias due to deviations from intended interventions		
**If your aim for this study is to assess the effect of assignment to intervention, answer questions 4.1 and 4.2**		
4.1. Were there deviations from the intended intervention beyond what would be expected in usual practice?		Y / PY / PN / N / NI
4.2. **If Y/PY to 4.1**: Were these deviations from intended intervention unbalanced between groups *and* likely to have affected the outcome?		NA / Y / PY / PN / N / NI
**If your aim for this study is to assess the effect of starting and adhering to intervention, answer questions 4.3 to 4.6**		
4.3. Were important co-interventions balanced across intervention groups?		Y / PY / PN / N / NI
4.4. Was the intervention implemented successfully for most participants?		Y / PY / PN / N / NI
4.5. Did study participants adhere to the assigned intervention regimen?		Y / PY / PN / N / NI
4.6. **If N/PN to 4.3, 4.4 or 4.5**: Was an appropriate analysis used to estimate the effect of starting and adhering to the intervention?		NA / Y / PY / PN / N / NI
**Risk of bias judgement**		Low / Moderate / Serious / Critical / NI
Optional: What is the predicted direction of bias due to deviations from the intended interventions?		Favours experimental / Favours comparator / Towards null /Away from null / Unpredictable

Bias due to missing data		
5.1 Were outcome data available for all, or nearly all, participants?		Y / PY / PN / N / NI
5.2 Were participants excluded due to missing data on intervention status?		Y / PY / PN / N / NI
5.3 Were participants excluded due to missing data on other variables needed for the analysis?		Y / PY / PN / N / NI
5.4 **If PN/N to 5.1, or Y/PY to 5.2 or 5.3**: Are the proportion of participants and reasons for missing data similar across interventions?		NA / Y / PY / PN / N / NI
5.5 **If PN/N to 5.1, or Y/PY to 5.2 or 5.3**: Is there evidence that results were robust to the presence of missing data?		NA / Y / PY / PN / N / NI
**Risk of bias judgement**		Low / Moderate / Serious / Critical / NI
Optional: What is the predicted direction of bias due to missing data?		Favours experimental / Favours comparator / Towards null /Away from null / Unpredictable

Bias in measurement of outcomes		
6.1 Could the outcome measure have been influenced by knowledge of the intervention received?		Y / PY / PN / N / NI
6.2 Were outcome assessors aware of the intervention received by study participants?		Y / PY / PN / N / NI
6.3 Were the methods of outcome assessment comparable across intervention groups?		Y / PY / PN / N / NI
6.4 Were any systematic errors in measurement of the outcome related to intervention received?		Y / PY / PN / N / NI
**Risk of bias judgement**		Low / Moderate / Serious / Critical / NI
Optional: What is the predicted direction of bias due to measurement of outcomes?		Favours experimental / Favours comparator / Towards null /Away from null / Unpredictable

Bias in selection of the reported result		
Is the reported effect estimate likely to be selected, on the basis of the results, from... 7.1. ... multiple outcome *measurements* within the outcome domain?		Y / PY / PN / N / NI
7.2 ... multiple *analyses* of the intervention-outcome relationship?		Y / PY / PN / N / NI
7.3 ... different *subgroups*?		Y / PY / PN / N / NI
**Risk of bias judgement**		Low / Moderate / Serious / Critical / NI
Optional: What is the predicted direction of bias due to selection of the reported result?		Favours experimental / Favours comparator / Towards null /Away from null / Unpredictable

Overall bias		
**Risk of bias judgement**		Low / Moderate / Serious / Critical / NI
Optional: What is the overall predicted direction of bias for this outcome?		Favours experimental / Favours comparator / Towards null /Away from null / Unpredictable

This work is licensed under a Creative Commons Attribution-NonCommercial-NoDerivatives 4.0 International License.

Domain	Recurrence
Bias due to confounding	Serious risk
Bias in selection of participants into the study	Low risk
Bias in classification of interventions	Low risk
Bias due to deviations from intended interventions	Low risk
Bias due to missing data	Serious risk
Bias in measurement of outcomes	Low risk
Bias in selection of the reported result	Moderate risk
**Overall**	**Serious risk**

Liu 2022

Specify a target randomized trial specific to the study

Design	Individually randomized / Cluster randomized / Matched (e.g. cross-over)
Participants	Patients with histopathological diagnosis of AIS after cervical excision
Experimental intervention	Conservative treatment
Comparator	Demolitive treatment (hysterectomy)

Is your aim for this study…?

- ■ to assess the effect of *assignment to* intervention
- ☐ to assess the effect of *starting and adhering to* intervention

Specify the outcome

Specify which outcome is being assessed for risk of bias (typically from among those earmarked for the Summary of Findings table). Specify whether this is a proposed benefit or harm of intervention.

Recurrence

Residual

Specify the numerical result being assessed

In case of multiple alternative analyses being presented, specify the numeric result (e.g. RR = 1.52 (95% CI 0.83 to 2.77) and/or a reference (e.g. to a table, figure or paragraph) that uniquely defines the result being assessed.

Preliminary consideration of confounders

Complete a row for each important confounding domain (i) listed in the review protocol; and (ii) relevant to the setting of this particular study, or which the study authors identified as potentially important.

"Important" confounding domains are those for which, in the context of this study, adjustment is expected to lead to a clinically important change in the estimated effect of the intervention. "Validity" refers to whether the confounding variable or variables fully measure the domain, while "reliability" refers to the precision of the measurement (more measurement error means less reliability).

**(i) Confounding domains listed in the review protocol**				
Confounding domain	Measured variable(s)	Is there evidence that controlling for this variable was unnecessary?*	Is the confounding domain measured validly and reliably by this variable (or these variables)?	OPTIONAL: Is failure to adjust for this variable (alone) expected to favour the experimental intervention or the comparator?
Age	Age ≤ 30 / Age > 30	No	Yes	-

**(ii) Additional confounding domains relevant to the setting of this particular study, or which the study authors identified as important**				
Confounding domain	Measured variable(s)	Is there evidence that controlling for this variable was unnecessary?*	Is the confounding domain measured validly and reliably by this variable (or these variables)?	OPTIONAL: Is failure to adjust for this variable (alone) expected to favour the experimental intervention or the comparator?
Histopathology	Co-existing CIN (yes/no)	No	Yes	-
Pre-operative cytology	Normal / Abnormal	No	Yes	-
Pre-operative HPV status	Positive / Negative	No	Yes	-
Post-operative cytology	Normal / Abnormal	No	Yes	-
Post-operative HPV status	Positive / Negative	No	Yes	-

Colposcopy	Transformation zone I&II / III	No	Yes	-
Excisional procedure	CKC / LEEP	No	Yes	-
Cone length(mm)	≥ 25 mm / < 25 mm	No	Yes	-
Margin	Positive / Negative	No	Yes	-

* In the context of a particular study, variables can be demonstrated not to be confounders and so not included in the analysis: (a) if they are not predictive of the outcome; (b) if they are not predictive of intervention; or (c) because adjustment makes no or minimal difference to the estimated effect of the primary parameter. Note that “no statistically significant association” is not the same as “not predictive”.

Preliminary consideration of co-interventions

Complete a row for each important co-intervention (i) listed in the review protocol; and (ii) relevant to the setting of this particular study, or which the study authors identified as important.

"Important" co-interventions are those for which, in the context of this study, adjustment is expected to lead to a clinically important change in the estimated effect of the intervention.

**(i) Co-interventions listed in the review protocol**		
Co-intervention	Is there evidence that controlling for this co-intervention was unnecessary (e.g. because it was not administered)?	Is presence of this co-intervention likely to favour outcomes in the experimental intervention or the comparator
None	-	-

**(ii) Additional co-interventions relevant to the setting of this particular study, or which the study authors identified as important**		
Co-intervention	Is there evidence that controlling for this co-intervention was unnecessary (e.g. because it was not administered)?	Is presence of this co-intervention likely to favour outcomes in the experimental intervention or the comparator
None	-	-

Risk of bias assessment

Responses underlined in green are potential markers for low risk of bias, and responses in red are potential markers for a risk of bias. Where questions relate only to sign posts to other questions, no formatting is used.

Signalling questions	Description	Response options
**Bias due to confounding**		
1.1 Is there potential for confounding of the effect of intervention in this study?<br>**If N/PN to 1.1:** the study can be considered to be at low risk of bias due to confounding and no further signalling questions need be considered		Y / PY / PN / N
**If Y/PY to 1.1**: determine whether there is a need to assess time-varying confounding:		
1.2. Was the analysis based on splitting participants' follow up time according to intervention received?<br>**If N/PN**, answer questions relating to baseline confounding (1.4 to 1.6)<br>**If Y/PY**, go to question 1.3.		NA / Y / PY / PN / N / NI
1.3. Were intervention discontinuations or switches likely to be related to factors that are prognostic for the outcome?<br>**If N/PN**, answer questions relating to baseline confounding (1.4 to 1.6)<br>**If Y/PY**, answer questions relating to both baseline and time-varying confounding (1.7 and 1.8)		NA / Y / PY / PN / N / NI

**Questions relating to baseline confounding only**		
1.4. Did the authors use an appropriate analysis method that controlled for all the important confounding domains?		NA / Y / PY / PN / N / NI
1.5. **If Y/PY to 1.4**: Were confounding domains that were controlled for measured validly and reliably by the variables available in this study?		NA / Y / PY / PN / N / NI
1.6. Did the authors control for any post-intervention variables that could have been affected by the intervention?		NA / Y / PY / PN / N / NI
**Questions relating to baseline and time-varying confounding**		
1.7. Did the authors use an appropriate analysis method that controlled for all the important confounding domains and for time-varying confounding?		NA / Y / PY / PN / N / NI
1.8. **If Y/PY to 1.7**: Were confounding domains that were controlled for measured validly and reliably by the variables available in this study?		NA / Y / PY / PN / N / NI
**Risk of bias judgement**		Low / Moderate / Serious / Critical / NI
Optional: What is the predicted direction of bias due to confounding?		Favours experimental / Favours comparator / Unpredictable

Bias in selection of participants into the study		
2.1. Was selection of participants into the study (or into the analysis) based on participant characteristics observed after the start of intervention?<br>**If N/PN to 2.1:** go to 2.4		Y / PY / PN / N / NI
2.2. **If Y/PY to 2.1**: Were the post-intervention variables that influenced selection likely to be associated with intervention?		NA / Y / PY / PN / N / NI
2.3 **If Y/PY to 2.2**: Were the post-intervention variables that influenced selection likely to be influenced by the outcome or a cause of the outcome?		NA / Y / PY / PN / N / NI
2.4. Do start of follow-up and start of intervention coincide for most participants?		Y / PY / PN / N / NI
2.5. **If Y/PY to 2.2 and 2.3, or N/PN to 2.4**: Were adjustment techniques used that are likely to correct for the presence of selection biases?		NA / Y / PY / PN / N / NI
**Risk of bias judgement**		Low / Moderate / Serious / Critical / NI
Optional: What is the predicted direction of bias due to selection of participants into the study?		Favours experimental / Favours comparator / Towards null /Away from null / Unpredictable

Bias in classification of interventions		
3.1 Were intervention groups clearly defined?		Y / PY / PN / N / NI
3.2 Was the information used to define intervention groups recorded at the start of the intervention?		Y / PY / PN / N / NI
3.3 Could classification of intervention status have been affected by knowledge of the outcome or risk of the outcome?		Y / PY / PN / N / NI
**Risk of bias judgement**		Low / Moderate / Serious / Critical / NI
Optional: What is the predicted direction of bias due to classification of interventions?		Favours experimental / Favours comparator / Towards null /Away from null / Unpredictable

Bias due to deviations from intended interventions		
**If your aim for this study is to assess the effect of assignment to intervention, answer questions 4.1 and 4.2**		
4.1. Were there deviations from the intended intervention beyond what would be expected in usual practice?		Y / PY / PN / N / NI
4.2. **If Y/PY to 4.1**: Were these deviations from intended intervention unbalanced between groups *and* likely to have affected the outcome?		NA / Y / PY / PN / N / NI
**If your aim for this study is to assess the effect of starting and adhering to intervention, answer questions 4.3 to 4.6**		
4.3. Were important co-interventions balanced across intervention groups?		Y / PY / PN / N / NI
4.4. Was the intervention implemented successfully for most participants?		Y / PY / PN / N / NI
4.5. Did study participants adhere to the assigned intervention regimen?		Y / PY / PN / N / NI
4.6. **If N/PN to 4.3, 4.4 or 4.5**: Was an appropriate analysis used to estimate the effect of starting and adhering to the intervention?		NA / Y / PY / PN / N / NI
**Risk of bias judgement**		Low / Moderate / Serious / Critical / NI
Optional: What is the predicted direction of bias due to deviations from the intended interventions?		Favours experimental / Favours comparator / Towards null /Away from null / Unpredictable

Bias due to missing data		
5.1 Were outcome data available for all, or nearly all, participants?		Y / PY / PN / N / NI
5.2 Were participants excluded due to missing data on intervention status?		Y / PY / PN / N / NI
5.3 Were participants excluded due to missing data on other variables needed for the analysis?		Y / PY / PN / N / NI
5.4 **If PN/N to 5.1, or Y/PY to 5.2 or 5.3**: Are the proportion of participants and reasons for missing data similar across interventions?		NA / Y / PY / PN / N / NI
5.5 **If PN/N to 5.1, or Y/PY to 5.2 or 5.3**: Is there evidence that results were robust to the presence of missing data?		NA / Y / PY / PN / N / NI
**Risk of bias judgement**		Low / Moderate / Serious / Critical / NI
Optional: What is the predicted direction of bias due to missing data?		Favours experimental / Favours comparator / Towards null /Away from null / Unpredictable

Bias in measurement of outcomes		
6.1 Could the outcome measure have been influenced by knowledge of the intervention received?		Y / PY / PN / N / NI
6.2 Were outcome assessors aware of the intervention received by study participants?		Y / PY / PN / N / NI
6.3 Were the methods of outcome assessment comparable across intervention groups?		Y / PY / PN / N / NI
6.4 Were any systematic errors in measurement of the outcome related to intervention received?		Y / PY / PN / N / NI
**Risk of bias judgement**		Low / Moderate / Serious / Critical / NI
Optional: What is the predicted direction of bias due to measurement of outcomes?		Favours experimental / Favours comparator / Towards null /Away from null / Unpredictable

Bias in selection of the reported result		
Is the reported effect estimate likely to be selected, on the basis of the results, from... 7.1. ... multiple outcome *measurements* within the outcome domain?		Y / PY / PN / N / NI
7.2 ... multiple *analyses* of the intervention-outcome relationship?		Y / PY / PN / N / NI
7.3 ... different *subgroups*?		Y / PY / PN / N / NI
**Risk of bias judgement**		Low / Moderate / Serious / Critical / NI
Optional: What is the predicted direction of bias due to selection of the reported result?		Favours experimental / Favours comparator / Towards null /Away from null / Unpredictable

Overall bias		
**Risk of bias judgement**		Low / Moderate / Serious / Critical / NI
Optional: What is the overall predicted direction of bias for this outcome?		Favours experimental / Favours comparator / Towards null /Away from null / Unpredictable

This work is licensed under a Creative Commons Attribution-NonCommercial-NoDerivatives 4.0 International License.

**Domain**	**Recurrence**	**Residual**
Bias due to confounding	Moderate risk	Serious risk
Bias in selection of participants into the study	Low risk	Low risk
Bias in classification of interventions	Low risk	Low risk
Bias due to deviations from intended interventions	Low risk	Low risk
Bias due to missing data	Low risk	Low risk
Bias in measurement of outcomes	Low risk	Low risk
Bias in selection of the reported result	Moderate risk	Moderate risk
**Overall**	**Moderate risk**	**Moderate risk**

Omnes 2003

Specify a target randomized trial specific to the study

Design	Individually randomized / Cluster randomized / Matched (e.g. cross-over)
Participants	Patients with histopathological diagnosis of AIS after cervical excision
Experimental intervention	Conservative treatment
Comparator	Demolitive treatment (hysterectomy)

Is your aim for this study…?

- ■ to assess the effect of *assignment to* intervention
- ☐ to assess the effect of *starting and adhering to* intervention

Specify the outcome

Specify which outcome is being assessed for risk of bias (typically from among those earmarked for the Summary of Findings table). Specify whether this is a proposed benefit or harm of intervention.

Residual

Specify the numerical result being assessed

In case of multiple alternative analyses being presented, specify the numeric result (e.g. RR = 1.52 (95% CI 0.83 to 2.77) and/or a reference (e.g. to a table, figure or paragraph) that uniquely defines the result being assessed.

Preliminary consideration of confounders

Complete a row for each important confounding domain (i) listed in the review protocol; and (ii) relevant to the setting of this particular study, or which the study authors identified as potentially important.

"Important" confounding domains are those for which, in the context of this study, adjustment is expected to lead to a clinically important change in the estimated effect of the intervention. "Validity" refers to whether the confounding variable or variables fully measure the domain, while "reliability" refers to the precision of the measurement (more measurement error means less reliability).

**(i) Confounding domains listed in the review protocol**				
Confounding domain	Measured variable(s)	Is there evidence that controlling for this variable was unnecessary?*	Is the confounding domain measured validly and reliably by this variable (or these variables)?	OPTIONAL: Is failure to adjust for this variable (alone) expected to favour the experimental intervention or the comparator?
Age	Not evaluated as confounder	No	-	-

**(ii) Additional confounding domains relevant to the setting of this particular study, or which the study authors identified as important**				
Confounding domain	Measured variable(s)	Is there evidence that controlling for this variable was unnecessary?*	Is the confounding domain measured validly and reliably by this variable (or these variables)?	OPTIONAL: Is failure to adjust for this variable (alone) expected to favour the experimental intervention or the comparator?
-	-	-	-	-

* In the context of a particular study, variables can be demonstrated not to be confounders and so not included in the analysis: (a) if they are not predictive of the outcome; (b) if they are not predictive of intervention; or (c) because adjustment makes no or minimal difference to the estimated effect of the primary parameter. Note that "no statistically significant association" is not the same as "not predictive".

Preliminary consideration of co-interventions

Complete a row for each important co-intervention (i) listed in the review protocol; and (ii) relevant to the setting of this particular study, or which the study authors identified as important.

"Important" co-interventions are those for which, in the context of this study, adjustment is expected to lead to a clinically important change in the estimated effect of the intervention.

**(i) Co-interventions listed in the review protocol**		
Co-intervention	Is there evidence that controlling for this co-intervention was unnecessary (e.g. because it was not administered)?	Is presence of this co-intervention likely to favour outcomes in the experimental intervention or the comparator
None	-	-

**(ii) Additional co-interventions relevant to the setting of this particular study, or which the study authors identified as important**		
Co-intervention	Is there evidence that controlling for this co-intervention was unnecessary (e.g. because it was not administered)?	Is presence of this co-intervention likely to favour outcomes in the experimental intervention or the comparator
None	-	-

Risk of bias assessment

Responses underlined in green are potential markers for low risk of bias, and responses in red are potential markers for a risk of bias. Where questions relate only to sign posts to other questions, no formatting is used.

Signalling questions	Description	Response options
**Bias due to confounding**		
1.1 Is there potential for confounding of the effect of intervention in this study?<br>**If N/PN to 1.1:** the study can be considered to be at low risk of bias due to confounding and no further signalling questions need be considered		Y / PY / PN / N
**If Y/PY to 1.1**: determine whether there is a need to assess time-varying confounding:		
1.2. Was the analysis based on splitting participants' follow up time according to intervention received?<br>**If N/PN**, answer questions relating to baseline confounding (1.4 to 1.6)<br>**If Y/PY**, go to question 1.3.		NA / Y / PY / PN / N / NI
1.3. Were intervention discontinuations or switches likely to be related to factors that are prognostic for the outcome?<br>**If N/PN**, answer questions relating to baseline confounding (1.4 to 1.6)<br>**If Y/PY**, answer questions relating to both baseline and time-varying confounding (1.7 and 1.8)		NA / Y / PY / PN / N / NI

**Questions relating to baseline confounding only**		
1.4. Did the authors use an appropriate analysis method that controlled for all the important confounding domains?		NA / Y / PY / PN / N / NI
1.5. **If Y/PY to 1.4**: Were confounding domains that were controlled for measured validly and reliably by the variables available in this study?		NA / Y / PY / PN / N / NI
1.6. Did the authors control for any post-intervention variables that could have been affected by the intervention?		NA / Y / PY / PN / N / NI
**Questions relating to baseline and time-varying confounding**		
1.7. Did the authors use an appropriate analysis method that controlled for all the important confounding domains and for time-varying confounding?		NA / Y / PY / PN / N / NI
1.8. **If Y/PY to 1.7**: Were confounding domains that were controlled for measured validly and reliably by the variables available in this study?		NA / Y / PY / PN / N / NI
**Risk of bias judgement**		Low / Moderate / Serious / Critical / NI
Optional: What is the predicted direction of bias due to confounding?		Favours experimental / Favours comparator / Unpredictable

Bias in selection of participants into the study		
2.1. Was selection of participants into the study (or into the analysis) based on participant characteristics observed after the start of intervention?<br>**If N/PN to 2.1:** go to 2.4		Y / PY / PN / N / NI
2.2. **If Y/PY to 2.1**: Were the post-intervention variables that influenced selection likely to be associated with intervention?		NA / Y / PY / PN / N / NI
2.3 **If Y/PY to 2.2**: Were the post-intervention variables that influenced selection likely to be influenced by the outcome or a cause of the outcome?		NA / Y / PY / PN / N / NI
2.4. Do start of follow-up and start of intervention coincide for most participants?		Y / PY / PN / N / NI
2.5. **If Y/PY to 2.2 and 2.3, or N/PN to 2.4**: Were adjustment techniques used that are likely to correct for the presence of selection biases?		NA / Y / PY / PN / N / NI
**Risk of bias judgement**		Low / Moderate / Serious / Critical / NI
Optional: What is the predicted direction of bias due to selection of participants into the study?		Favours experimental / Favours comparator / Towards null /Away from null / Unpredictable

Bias in classification of interventions		
3.1 Were intervention groups clearly defined?		Y / PY / PN / N / NI
3.2 Was the information used to define intervention groups recorded at the start of the intervention?		Y / PY / PN / N / NI
3.3 Could classification of intervention status have been affected by knowledge of the outcome or risk of the outcome?		Y / PY / PN / N / NI
**Risk of bias judgement**		Low / Moderate / Serious / Critical / NI
Optional: What is the predicted direction of bias due to classification of interventions?		Favours experimental / Favours comparator / Towards null /Away from null / Unpredictable

Bias due to deviations from intended interventions		
**If your aim for this study is to assess the effect of assignment to intervention, answer questions 4.1 and 4.2**		
4.1. Were there deviations from the intended intervention beyond what would be expected in usual practice?		Y / PY / PN / N / NI
4.2. **If Y/PY to 4.1**: Were these deviations from intended intervention unbalanced between groups *and* likely to have affected the outcome?		NA / Y / PY / PN / N / NI
**If your aim for this study is to assess the effect of starting and adhering to intervention, answer questions 4.3 to 4.6**		
4.3. Were important co-interventions balanced across intervention groups?		Y / PY / PN / N / NI
4.4. Was the intervention implemented successfully for most participants?		Y / PY / PN / N / NI
4.5. Did study participants adhere to the assigned intervention regimen?		Y / PY / PN / N / NI
4.6. **If N/PN to 4.3, 4.4 or 4.5**: Was an appropriate analysis used to estimate the effect of starting and adhering to the intervention?		NA / Y / PY / PN / N / NI
**Risk of bias judgement**		Low / Moderate / Serious / Critical / NI
Optional: What is the predicted direction of bias due to deviations from the intended interventions?		Favours experimental / Favours comparator / Towards null /Away from null / Unpredictable

Bias due to missing data		
5.1 Were outcome data available for all, or nearly all, participants?		Y / PY / PN / N / NI
5.2 Were participants excluded due to missing data on intervention status?		Y / PY / PN / N / NI
5.3 Were participants excluded due to missing data on other variables needed for the analysis?		Y / PY / PN / N / NI
5.4 **If PN/N to 5.1, or Y/PY to 5.2 or 5.3**: Are the proportion of participants and reasons for missing data similar across interventions?		NA / Y / PY / PN / N / NI
5.5 **If PN/N to 5.1, or Y/PY to 5.2 or 5.3**: Is there evidence that results were robust to the presence of missing data?		NA / Y / PY / PN / N / NI
**Risk of bias judgement**		Low / Moderate / Serious / Critical / NI
Optional: What is the predicted direction of bias due to missing data?		Favours experimental / Favours comparator / Towards null /Away from null / Unpredictable

Bias in measurement of outcomes		
6.1 Could the outcome measure have been influenced by knowledge of the intervention received?		Y / PY / PN / N / NI
6.2 Were outcome assessors aware of the intervention received by study participants?		Y / PY / PN / N / NI
6.3 Were the methods of outcome assessment comparable across intervention groups?		Y / PY / PN / N / NI
6.4 Were any systematic errors in measurement of the outcome related to intervention received?		Y / PY / PN / N / NI
**Risk of bias judgement**		Low / Moderate / Serious / Critical / NI
Optional: What is the predicted direction of bias due to measurement of outcomes?		Favours experimental / Favours comparator / Towards null /Away from null / Unpredictable

Bias in selection of the reported result		
Is the reported effect estimate likely to be selected, on the basis of the results, from...<br>7.1. ... multiple outcome *measurements* within the outcome domain?		Y / PY / PN / N / NI
7.2 ... multiple *analyses* of the intervention-outcome relationship?		Y / PY / PN / N / NI
7.3 ... different *subgroups*?		Y / PY / PN / N / NI
**Risk of bias judgement**		Low / Moderate / Serious / Critical / NI
Optional: What is the predicted direction of bias due to selection of the reported result?		Favours experimental / Favours comparator / Towards null /Away from null / Unpredictable

Overall bias		
**Risk of bias judgement**		Low / Moderate / Serious / Critical / NI
Optional: What is the overall predicted direction of bias for this outcome?		Favours experimental / Favours comparator / Towards null /Away from null / Unpredictable

This work is licensed under a Creative Commons Attribution-NonCommercial-NoDerivatives 4.0 International License.

**Domain**	**Recurrence**
Bias due to confounding	Serious risk
Bias in selection of participants into the study	Low risk
Bias in classification of interventions	Low risk
Bias due to deviations from intended interventions	Low risk
Bias due to missing data	Low risk
Bias in measurement of outcomes	Low risk
Bias in selection of the reported result	Moderate risk
**Overall**	**Serious risk**

Schaafsma 2024

Specify a target randomized trial specific to the study

Design	Individually randomized / Cluster randomized / Matched (e.g. cross-over)
Participants	Patients with histopathological diagnosis of AIS after cervical excision
Experimental intervention	Conservative treatment
Comparator	Demolitive treatment (hysterectomy)

Is your aim for this study…?

- ■ to assess the effect of *assignment to* intervention
- ☐ to assess the effect of *starting and adhering to* intervention

Specify the outcome

Specify which outcome is being assessed for risk of bias (typically from among those earmarked for the Summary of Findings table). Specify whether this is a proposed benefit or harm of intervention.

Recurrence

Specify the numerical result being assessed

In case of multiple alternative analyses being presented, specify the numeric result (e.g. RR = 1.52 (95% CI 0.83 to 2.77) and/or a reference (e.g. to a table, figure or paragraph) that uniquely defines the result being assessed.

Preliminary consideration of confounders

Complete a row for each important confounding domain (i) listed in the review protocol; and (ii) relevant to the setting of this particular study, or which the study authors identified as potentially important.

"Important" confounding domains are those for which, in the context of this study, adjustment is expected to lead to a clinically important change in the estimated effect of the intervention. "Validity" refers to whether the confounding variable or variables fully measure the domain, while "reliability" refers to the precision of the measurement (more measurement error means less reliability).

**(i) Confounding domains listed in the review protocol**				
Confounding domain	Measured variable(s)	Is there evidence that controlling for this variable was unnecessary?*	Is the confounding domain measured validly and reliably by this variable (or these variables)?	OPTIONAL: Is failure to adjust for this variable (alone) expected to favour the experimental intervention or the comparator?
Age	Not evaluated as confounder	No	-	-

**(ii) Additional confounding domains relevant to the setting of this particular study, or which the study authors identified as important**				
Confounding domain	Measured variable(s)	Is there evidence that controlling for this variable was unnecessary?*	Is the confounding domain measured validly and reliably by this variable (or these variables)?	OPTIONAL: Is failure to adjust for this variable (alone) expected to favour the experimental intervention or the comparator?
-	-	-	-	-

* In the context of a particular study, variables can be demonstrated not to be confounders and so not included in the analysis: (a) if they are not predictive of the outcome; (b) if they are not predictive of intervention; or (c) because adjustment makes no or minimal difference to the estimated effect of the primary parameter. Note that "no statistically significant association" is not the same as "not predictive".

Preliminary consideration of co-interventions

Complete a row for each important co-intervention (i) listed in the review protocol; and (ii) relevant to the setting of this particular study, or which the study authors identified as important.

"Important" co-interventions are those for which, in the context of this study, adjustment is expected to lead to a clinically important change in the estimated effect of the intervention.

**(i) Co-interventions listed in the review protocol**		
Co-intervention	Is there evidence that controlling for this co-intervention was unnecessary (e.g. because it was not administered)?	Is presence of this co-intervention likely to favour outcomes in the experimental intervention or the comparator
None	-	-

**(ii) Additional co-interventions relevant to the setting of this particular study, or which the study authors identified as important**		
Co-intervention	Is there evidence that controlling for this co-intervention was unnecessary (e.g. because it was not administered)?	Is presence of this co-intervention likely to favour outcomes in the experimental intervention or the comparator
None	-	-

Risk of bias assessment

Responses underlined in green are potential markers for low risk of bias, and responses in red are potential markers for a risk of bias. Where questions relate only to sign posts to other questions, no formatting is used.

Signalling questions	Description	Response options
**Bias due to confounding**		
1.1 Is there potential for confounding of the effect of intervention in this study?<br>**If N/PN to 1.1:** the study can be considered to be at low risk of bias due to confounding and no further signalling questions need be considered		Y / PY / PN / N
**If Y/PY to 1.1**: determine whether there is a need to assess time-varying confounding:		
1.2. Was the analysis based on splitting participants' follow up time according to intervention received?<br>**If N/PN**, answer questions relating to baseline confounding (1.4 to 1.6)<br>**If Y/PY**, go to question 1.3.		NA / Y / PY / PN / N / NI
1.3. Were intervention discontinuations or switches likely to be related to factors that are prognostic for the outcome?<br>**If N/PN**, answer questions relating to baseline confounding (1.4 to 1.6)<br>**If Y/PY**, answer questions relating to both baseline and time-varying confounding (1.7 and 1.8)		NA / Y / PY / PN / N / NI

**Questions relating to baseline confounding only**		
1.4. Did the authors use an appropriate analysis method that controlled for all the important confounding domains?		NA / Y / PY / PN / N / NI
1.5. **If Y/PY to 1.4**: Were confounding domains that were controlled for measured validly and reliably by the variables available in this study?		NA / Y / PY / PN / N / NI
1.6. Did the authors control for any post-intervention variables that could have been affected by the intervention?		NA / Y / PY / PN / N / NI
**Questions relating to baseline and time-varying confounding**		
1.7. Did the authors use an appropriate analysis method that controlled for all the important confounding domains and for time-varying confounding?		NA / Y / PY / PN / N / NI
1.8. **If Y/PY to 1.7**: Were confounding domains that were controlled for measured validly and reliably by the variables available in this study?		NA / Y / PY / PN / N / NI
**Risk of bias judgement**		Low / Moderate / Serious / Critical / NI
Optional: What is the predicted direction of bias due to confounding?		Favours experimental / Favours comparator / Unpredictable

Bias in selection of participants into the study		
2.1. Was selection of participants into the study (or into the analysis) based on participant characteristics observed after the start of intervention?<br>**If N/PN to 2.1:** go to 2.4		Y / PY / PN / N / NI
2.2. **If Y/PY to 2.1**: Were the post-intervention variables that influenced selection likely to be associated with intervention?		NA / Y / PY / PN / N / NI
2.3 **If Y/PY to 2.2**: Were the post-intervention variables that influenced selection likely to be influenced by the outcome or a cause of the outcome?		NA / Y / PY / PN / N / NI
2.4. Do start of follow-up and start of intervention coincide for most participants?		Y / PY / PN / N / NI
2.5. **If Y/PY to 2.2 and 2.3, or N/PN to 2.4**: Were adjustment techniques used that are likely to correct for the presence of selection biases?		NA / Y / PY / PN / N / NI
**Risk of bias judgement**		Low / Moderate / Serious / Critical / NI
Optional: What is the predicted direction of bias due to selection of participants into the study?		Favours experimental / Favours comparator / Towards null /Away from null / Unpredictable

Bias in classification of interventions		
3.1 Were intervention groups clearly defined?		Y / PY / PN / N / NI
3.2 Was the information used to define intervention groups recorded at the start of the intervention?		Y / PY / PN / N / NI
3.3 Could classification of intervention status have been affected by knowledge of the outcome or risk of the outcome?		Y / PY / PN / N / NI
**Risk of bias judgement**		Low / Moderate / Serious / Critical / NI
Optional: What is the predicted direction of bias due to classification of interventions?		Favours experimental / Favours comparator / Towards null /Away from null / Unpredictable

Bias due to deviations from intended interventions		
**If your aim for this study is to assess the effect of assignment to intervention, answer questions 4.1 and 4.2**		
4.1. Were there deviations from the intended intervention beyond what would be expected in usual practice?		Y / PY / PN / N / NI
4.2. **If Y/PY to 4.1**: Were these deviations from intended intervention unbalanced between groups *and* likely to have affected the outcome?		NA / Y / PY / PN / N / NI
**If your aim for this study is to assess the effect of starting and adhering to intervention, answer questions 4.3 to 4.6**		
4.3. Were important co-interventions balanced across intervention groups?		Y / PY / PN / N / NI
4.4. Was the intervention implemented successfully for most participants?		Y / PY / PN / N / NI
4.5. Did study participants adhere to the assigned intervention regimen?		Y / PY / PN / N / NI
4.6. **If N/PN to 4.3, 4.4 or 4.5**: Was an appropriate analysis used to estimate the effect of starting and adhering to the intervention?		NA / Y / PY / PN / N / NI
**Risk of bias judgement**		Low / Moderate / Serious / Critical / NI
Optional: What is the predicted direction of bias due to deviations from the intended interventions?		Favours experimental / Favours comparator / Towards null /Away from null / Unpredictable

Bias due to missing data		
5.1 Were outcome data available for all, or nearly all, participants?		Y / PY / PN / N / NI
5.2 Were participants excluded due to missing data on intervention status?		Y / PY / PN / N / NI
5.3 Were participants excluded due to missing data on other variables needed for the analysis?		Y / PY / PN / N / NI
5.4 **If PN/N to 5.1, or Y/PY to 5.2 or 5.3**: Are the proportion of participants and reasons for missing data similar across interventions?		NA / Y / PY / PN / N / NI
5.5 **If PN/N to 5.1, or Y/PY to 5.2 or 5.3**: Is there evidence that results were robust to the presence of missing data?		NA / Y / PY / PN / N / NI
**Risk of bias judgement**		Low / Moderate / Serious / Critical / NI
Optional: What is the predicted direction of bias due to missing data?		Favours experimental / Favours comparator / Towards null /Away from null / Unpredictable

Bias in measurement of outcomes		
6.1 Could the outcome measure have been influenced by knowledge of the intervention received?		Y / PY / PN / N / NI
6.2 Were outcome assessors aware of the intervention received by study participants?		Y / PY / PN / N / NI
6.3 Were the methods of outcome assessment comparable across intervention groups?		Y / PY / PN / N / NI
6.4 Were any systematic errors in measurement of the outcome related to intervention received?		Y / PY / PN / N / NI
**Risk of bias judgement**		Low / Moderate / Serious / Critical / NI
Optional: What is the predicted direction of bias due to measurement of outcomes?		Favours experimental / Favours comparator / Towards null /Away from null / Unpredictable

Bias in selection of the reported result		
Is the reported effect estimate likely to be selected, on the basis of the results, from... 7.1. ... multiple outcome *measurements* within the outcome domain?		Y / PY / PN / N / NI
7.2 ... multiple *analyses* of the intervention-outcome relationship?		Y / PY / PN / N / NI
7.3 ... different *subgroups*?		Y / PY / PN / N / NI
**Risk of bias judgement**		Low / Moderate / Serious / Critical / NI
Optional: What is the predicted direction of bias due to selection of the reported result?		Favours experimental / Favours comparator / Towards null /Away from null / Unpredictable

Overall bias		
**Risk of bias judgement**		Low / Moderate / Serious / Critical / NI
Optional: What is the overall predicted direction of bias for this outcome?		Favours experimental / Favours comparator / Towards null /Away from null / Unpredictable

This work is licensed under a Creative Commons Attribution-NonCommercial-NoDerivatives 4.0 International License.

**Domain**	**Recurrence**
Bias due to confounding	Serious risk
Bias in selection of participants into the study	Low risk
Bias in classification of interventions	Low risk
Bias due to deviations from intended interventions	Low risk
Bias due to missing data	Low risk
Bias in measurement of outcomes	Low risk
Bias in selection of the reported result	Moderate risk
**Overall**	**Serious risk**

Shin 2000

Specify a target randomized trial specific to the study

Design	Individually randomized / Cluster randomized / Matched (e.g. cross-over)
Participants	Patients with histopathological diagnosis of AIS after cervical excision
Experimental intervention	Conservative treatment
Comparator	Demolitive treatment (hysterectomy)

Is your aim for this study…?

- ■ to assess the effect of *assignment to* intervention
- ☐ to assess the effect of *starting and adhering to* intervention

Specify the outcome

Specify which outcome is being assessed for risk of bias (typically from among those earmarked for the Summary of Findings table). Specify whether this is a proposed benefit or harm of intervention.

Recurrence<br>Residual

Specify the numerical result being assessed

In case of multiple alternative analyses being presented, specify the numeric result (e.g. RR = 1.52 (95% CI 0.83 to 2.77) and/or a reference (e.g. to a table, figure or paragraph) that uniquely defines the result being assessed.

Preliminary consideration of confounders

Complete a row for each important confounding domain (i) listed in the review protocol; and (ii) relevant to the setting of this particular study, or which the study authors identified as potentially important.

"Important" confounding domains are those for which, in the context of this study, adjustment is expected to lead to a clinically important change in the estimated effect of the intervention. "Validity" refers to whether the confounding variable or variables fully measure the domain, while "reliability" refers to the precision of the measurement (more measurement error means less reliability).

**(i) Confounding domains listed in the review protocol**				
Confounding domain	Measured variable(s)	Is there evidence that controlling for this variable was unnecessary?*	Is the confounding domain measured validly and reliably by this variable (or these variables)?	OPTIONAL: Is failure to adjust for this variable (alone) expected to favour the experimental intervention or the comparator?
Age	Not evaluated as confounder	No	-	-

**(ii) Additional confounding domains relevant to the setting of this particular study, or which the study authors identified as important**				
Confounding domain	Measured variable(s)	Is there evidence that controlling for this variable was unnecessary?*	Is the confounding domain measured validly and reliably by this variable (or these variables)?	OPTIONAL: Is failure to adjust for this variable (alone) expected to favour the experimental intervention or the comparator?
-	-	-	-	-

* In the context of a particular study, variables can be demonstrated not to be confounders and so not included in the analysis: (a) if they are not predictive of the outcome; (b) if they are not predictive of intervention; or (c) because adjustment makes no or minimal difference to the estimated effect of the primary parameter. Note that "no statistically significant association" is not the same as "not predictive".

Preliminary consideration of co-interventions

Complete a row for each important co-intervention (i) listed in the review protocol; and (ii) relevant to the setting of this particular study, or which the study authors identified as important.

"Important" co-interventions are those for which, in the context of this study, adjustment is expected to lead to a clinically important change in the estimated effect of the intervention.

**(i) Co-interventions listed in the review protocol**		
Co-intervention	Is there evidence that controlling for this co-intervention was unnecessary (e.g. because it was not administered)?	Is presence of this co-intervention likely to favour outcomes in the experimental intervention or the comparator
None	-	-

**(ii) Additional co-interventions relevant to the setting of this particular study, or which the study authors identified as important**		
Co-intervention	Is there evidence that controlling for this co-intervention was unnecessary (e.g. because it was not administered)?	Is presence of this co-intervention likely to favour outcomes in the experimental intervention or the comparator
None	-	-

Risk of bias assessment

Responses underlined in green are potential markers for low risk of bias, and responses in red are potential markers for a risk of bias. Where questions relate only to sign posts to other questions, no formatting is used.

Signalling questions	Description	Response options
**Bias due to confounding**		
1.1 Is there potential for confounding of the effect of intervention in this study?<br>**If N/PN to 1.1:** the study can be considered to be at low risk of bias due to confounding and no further signalling questions need be considered		Y / PY / PN / N
**If Y/PY to 1.1**: determine whether there is a need to assess time-varying confounding:		
1.2. Was the analysis based on splitting participants' follow up time according to intervention received?<br>**If N/PN**, answer questions relating to baseline confounding (1.4 to 1.6)<br>**If Y/PY**, go to question 1.3.		NA / Y / PY / PN / N / NI
1.3. Were intervention discontinuations or switches likely to be related to factors that are prognostic for the outcome?<br>**If N/PN**, answer questions relating to baseline confounding (1.4 to 1.6)<br>**If Y/PY**, answer questions relating to both baseline and time-varying confounding (1.7 and 1.8)		NA / Y / PY / PN / N / NI

**Questions relating to baseline confounding only**		
1.4. Did the authors use an appropriate analysis method that controlled for all the important confounding domains?		NA / Y / PY / PN / N / NI
1.5. **If Y/PY to 1.4**: Were confounding domains that were controlled for measured validly and reliably by the variables available in this study?		NA / Y / PY / PN / N / NI
1.6. Did the authors control for any post-intervention variables that could have been affected by the intervention?		NA / Y / PY / PN / N / NI
**Questions relating to baseline and time-varying confounding**		
1.7. Did the authors use an appropriate analysis method that controlled for all the important confounding domains and for time-varying confounding?		NA / Y / PY / PN / N / NI
1.8. **If Y/PY to 1.7**: Were confounding domains that were controlled for measured validly and reliably by the variables available in this study?		NA / Y / PY / PN / N / NI
**Risk of bias judgement**		Low / Moderate / Serious / Critical / NI
Optional: What is the predicted direction of bias due to confounding?		Favours experimental / Favours comparator / Unpredictable

**Bias in selection of participants into the study**		
2.1. Was selection of participants into the study (or into the analysis) based on participant characteristics observed after the start of intervention?<br>**If N/PN to 2.1:** go to 2.4		Y / PY / PN / N / NI
2.2. **If Y/PY to 2.1**: Were the post-intervention variables that influenced selection likely to be associated with intervention?		NA / Y / PY / PN / N / NI
2.3 **If Y/PY to 2.2**: Were the post-intervention variables that influenced selection likely to be influenced by the outcome or a cause of the outcome?		NA / Y / PY / PN / N / NI
2.4. Do start of follow-up and start of intervention coincide for most participants?		Y / PY / PN / N / NI
2.5. **If Y/PY to 2.2 and 2.3, or N/PN to 2.4**: Were adjustment techniques used that are likely to correct for the presence of selection biases?		NA / Y / PY / PN / N / NI
**Risk of bias judgement**		Low / Moderate / Serious / Critical / NI
Optional: What is the predicted direction of bias due to selection of participants into the study?		Favours experimental / Favours comparator / Towards null /Away from null / Unpredictable

Bias in classification of interventions		
3.1 Were intervention groups clearly defined?		Y / PY / PN / N / NI
3.2 Was the information used to define intervention groups recorded at the start of the intervention?		Y / PY / PN / N / NI
3.3 Could classification of intervention status have been affected by knowledge of the outcome or risk of the outcome?		Y / PY / PN / N / NI
**Risk of bias judgement**		Low / Moderate / Serious / Critical / NI
Optional: What is the predicted direction of bias due to classification of interventions?		Favours experimental / Favours comparator / Towards null /Away from null / Unpredictable

Bias due to deviations from intended interventions		
**If your aim for this study is to assess the effect of assignment to intervention, answer questions 4.1 and 4.2**		
4.1. Were there deviations from the intended intervention beyond what would be expected in usual practice?		Y / PY / PN / N / NI
4.2. **If Y/PY to 4.1**: Were these deviations from intended intervention unbalanced between groups *and* likely to have affected the outcome?		NA / Y / PY / PN / N / NI
**If your aim for this study is to assess the effect of starting and adhering to intervention, answer questions 4.3 to 4.6**		
4.3. Were important co-interventions balanced across intervention groups?		Y / PY / PN / N / NI
4.4. Was the intervention implemented successfully for most participants?		Y / PY / PN / N / NI
4.5. Did study participants adhere to the assigned intervention regimen?		Y / PY / PN / N / NI
4.6. **If N/PN to 4.3, 4.4 or 4.5**: Was an appropriate analysis used to estimate the effect of starting and adhering to the intervention?		NA / Y / PY / PN / N / NI
**Risk of bias judgement**		Low / Moderate / Serious / Critical / NI
Optional: What is the predicted direction of bias due to deviations from the intended interventions?		Favours experimental / Favours comparator / Towards null /Away from null / Unpredictable

Bias due to missing data		
5.1 Were outcome data available for all, or nearly all, participants?		Y / PY / PN / N / NI
5.2 Were participants excluded due to missing data on intervention status?		Y / PY / PN / N / NI
5.3 Were participants excluded due to missing data on other variables needed for the analysis?		Y / PY / PN / N / NI
5.4 **If PN/N to 5.1, or Y/PY to 5.2 or 5.3**: Are the proportion of participants and reasons for missing data similar across interventions?		NA / Y / PY / PN / N / NI
5.5 **If PN/N to 5.1, or Y/PY to 5.2 or 5.3**: Is there evidence that results were robust to the presence of missing data?		NA / Y / PY / PN / N / NI
**Risk of bias judgement**		Low / Moderate / Serious / Critical / NI
Optional: What is the predicted direction of bias due to missing data?		Favours experimental / Favours comparator / Towards null /Away from null / Unpredictable

Bias in measurement of outcomes		
6.1 Could the outcome measure have been influenced by knowledge of the intervention received?		Y / PY / PN / N / NI
6.2 Were outcome assessors aware of the intervention received by study participants?		Y / PY / PN / N / NI
6.3 Were the methods of outcome assessment comparable across intervention groups?		Y / PY / PN / N / NI
6.4 Were any systematic errors in measurement of the outcome related to intervention received?		Y / PY / PN / N / NI
**Risk of bias judgement**		Low / Moderate / Serious / Critical / NI
Optional: What is the predicted direction of bias due to measurement of outcomes?		Favours experimental / Favours comparator / Towards null /Away from null / Unpredictable

Bias in selection of the reported result		
Is the reported effect estimate likely to be selected, on the basis of the results, from... 7.1. ... multiple outcome *measurements* within the outcome domain?		Y / PY / PN / N / NI
7.2 ... multiple *analyses* of the intervention-outcome relationship?		Y / PY / PN / N / NI
7.3 ... different *subgroups*?		Y / PY / PN / N / NI
**Risk of bias judgement**		Low / Moderate / Serious / Critical / NI
Optional: What is the predicted direction of bias due to selection of the reported result?		Favours experimental / Favours comparator / Towards null /Away from null / Unpredictable

Overall bias		
**Risk of bias judgement**		Low / Moderate / Serious / Critical / NI
Optional: What is the overall predicted direction of bias for this outcome?		Favours experimental / Favours comparator / Towards null /Away from null / Unpredictable

This work is licensed under a Creative Commons Attribution-NonCommercial-NoDerivatives 4.0 International License.

Domain	Recurrence	Residual
Bias due to confounding	Serious risk	Serious risk
Bias in selection of participants into the study	Low risk	Low risk
Bias in classification of interventions	Low risk	Low risk
Bias due to deviations from intended interventions	Low risk	Low risk
Bias due to missing data	Low risk	Low risk
Bias in measurement of outcomes	Low risk	Low risk
Bias in selection of the reported result	Moderate risk	Moderate risk
**Overall**	**Serious risk**	**Serious risk**

Song 2015

Specify a target randomized trial specific to the study

Design	Individually randomized / Cluster randomized / Matched (e.g. cross-over)
Participants	Patients with histopathological diagnosis of AIS after cervical excision
Experimental intervention	Conservative treatment
Comparator	Demolitive treatment (hysterectomy)

Is your aim for this study…?

- ■ to assess the effect of *assignment to* intervention
- ☐ to assess the effect of *starting and adhering to* intervention

Specify the outcome

Specify which outcome is being assessed for risk of bias (typically from among those earmarked for the Summary of Findings table). Specify whether this is a proposed benefit or harm of intervention.

Recurrence<br>Residual

Specify the numerical result being assessed

In case of multiple alternative analyses being presented, specify the numeric result (e.g. RR = 1.52 (95% CI 0.83 to 2.77) and/or a reference (e.g. to a table, figure or paragraph) that uniquely defines the result being assessed.

Preliminary consideration of confounders

Complete a row for each important confounding domain (i) listed in the review protocol; and (ii) relevant to the setting of this particular study, or which the study authors identified as potentially important.

"Important" confounding domains are those for which, in the context of this study, adjustment is expected to lead to a clinically important change in the estimated effect of the intervention. "Validity" refers to whether the confounding variable or variables fully measure the domain, while "reliability" refers to the precision of the measurement (more measurement error means less reliability).

**(i) Confounding domains listed in the review protocol**				
Confounding domain	Measured variable(s)	Is there evidence that controlling for this variable was unnecessary?*	Is the confounding domain measured validly and reliably by this variable (or these variables)?	OPTIONAL: Is failure to adjust for this variable (alone) expected to favour the experimental intervention or the comparator?
Age	Not evaluated as confounder	No	-	-

**(ii) Additional confounding domains relevant to the setting of this particular study, or which the study authors identified as important**				
Confounding domain	Measured variable(s)	Is there evidence that controlling for this variable was unnecessary?*	Is the confounding domain measured validly and reliably by this variable (or these variables)?	OPTIONAL: Is failure to adjust for this variable (alone) expected to favour the experimental intervention or the comparator?
-	-	-	-	-

* In the context of a particular study, variables can be demonstrated not to be confounders and so not included in the analysis: (a) if they are not predictive of the outcome; (b) if they are not predictive of intervention; or (c) because adjustment makes no or minimal difference to the estimated effect of the primary parameter. Note that "no statistically significant association" is not the same as "not predictive".

Preliminary consideration of co-interventions

Complete a row for each important co-intervention (i) listed in the review protocol; and (ii) relevant to the setting of this particular study, or which the study authors identified as important.

"Important" co-interventions are those for which, in the context of this study, adjustment is expected to lead to a clinically important change in the estimated effect of the intervention.

**(i) Co-interventions listed in the review protocol**		
Co-intervention	Is there evidence that controlling for this co-intervention was unnecessary (e.g. because it was not administered)?	Is presence of this co-intervention likely to favour outcomes in the experimental intervention or the comparator
None	-	-

**(ii) Additional co-interventions relevant to the setting of this particular study, or which the study authors identified as important**		
Co-intervention	Is there evidence that controlling for this co-intervention was unnecessary (e.g. because it was not administered)?	Is presence of this co-intervention likely to favour outcomes in the experimental intervention or the comparator
None	-	-

Risk of bias assessment

Responses underlined in green are potential markers for low risk of bias, and responses in red are potential markers for a risk of bias. Where questions relate only to sign posts to other questions, no formatting is used.

Signalling questions	Description	Response options
**Bias due to confounding**		
1.1 Is there potential for confounding of the effect of intervention in this study?<br><br>**If N/PN to 1.1:** the study can be considered to be at low risk of bias due to confounding and no further signalling questions need be considered		Y / PY / PN / N
**If Y/PY to 1.1**: determine whether there is a need to assess time-varying confounding:		
1.2. Was the analysis based on splitting participants' follow up time according to intervention received?<br><br>**If N/PN**, answer questions relating to baseline confounding (1.4 to 1.6)<br><br>**If Y/PY**, go to question 1.3.		NA / Y / PY / PN / N / NI
1.3. Were intervention discontinuations or switches likely to be related to factors that are prognostic for the outcome?<br><br>**If N/PN**, answer questions relating to baseline confounding (1.4 to 1.6)<br><br>**If Y/PY**, answer questions relating to both baseline and time-varying confounding (1.7 and 1.8)		NA / Y / PY / PN / N / NI

**Questions relating to baseline confounding only**		
1.4. Did the authors use an appropriate analysis method that controlled for all the important confounding domains?		NA / Y / PY / PN / N / NI
1.5. **If Y/PY to 1.4**: Were confounding domains that were controlled for measured validly and reliably by the variables available in this study?		NA / Y / PY / PN / N / NI
1.6. Did the authors control for any post-intervention variables that could have been affected by the intervention?		NA / Y / PY / PN / N / NI
**Questions relating to baseline and time-varying confounding**		
1.7. Did the authors use an appropriate analysis method that controlled for all the important confounding domains and for time-varying confounding?		NA / Y / PY / PN / N / NI
1.8. **If Y/PY to 1.7**: Were confounding domains that were controlled for measured validly and reliably by the variables available in this study?		NA / Y / PY / PN / N / NI
**Risk of bias judgement**		Low / Moderate / Serious / Critical / NI
Optional: What is the predicted direction of bias due to confounding?		Favours experimental / Favours comparator / Unpredictable

Bias in selection of participants into the study		
2.1. Was selection of participants into the study (or into the analysis) based on participant characteristics observed after the start of intervention?<br>**If N/PN to 2.1:** go to 2.4		Y / PY / PN / N / NI
2.2. **If Y/PY to 2.1**: Were the post-intervention variables that influenced selection likely to be associated with intervention?		NA / Y / PY / PN / N / NI
2.3 **If Y/PY to 2.2**: Were the post-intervention variables that influenced selection likely to be influenced by the outcome or a cause of the outcome?		NA / Y / PY / PN / N / NI
2.4. Do start of follow-up and start of intervention coincide for most participants?		Y / PY / PN / N / NI
2.5. **If Y/PY to 2.2 and 2.3, or N/PN to 2.4**: Were adjustment techniques used that are likely to correct for the presence of selection biases?		NA / Y / PY / PN / N / NI
**Risk of bias judgement**		Low / Moderate / Serious / Critical / NI
Optional: What is the predicted direction of bias due to selection of participants into the study?		Favours experimental / Favours comparator / Towards null /Away from null / Unpredictable

Bias in classification of interventions		
3.1 Were intervention groups clearly defined?		Y / PY / PN / N / NI
3.2 Was the information used to define intervention groups recorded at the start of the intervention?		Y / PY / PN / N / NI
3.3 Could classification of intervention status have been affected by knowledge of the outcome or risk of the outcome?		Y / PY / PN / N / NI
**Risk of bias judgement**		Low / Moderate / Serious / Critical / NI
Optional: What is the predicted direction of bias due to classification of interventions?		Favours experimental / Favours comparator / Towards null /Away from null / Unpredictable

Bias due to deviations from intended interventions		
**If your aim for this study is to assess the effect of assignment to intervention, answer questions 4.1 and 4.2**		
4.1. Were there deviations from the intended intervention beyond what would be expected in usual practice?		Y / PY / PN / N / NI
4.2. **If Y/PY to 4.1**: Were these deviations from intended intervention unbalanced between groups *and* likely to have affected the outcome?		NA / Y / PY / PN / N / NI
**If your aim for this study is to assess the effect of starting and adhering to intervention, answer questions 4.3 to 4.6**		
4.3. Were important co-interventions balanced across intervention groups?		Y / PY / PN / N / NI
4.4. Was the intervention implemented successfully for most participants?		Y / PY / PN / N / NI
4.5. Did study participants adhere to the assigned intervention regimen?		Y / PY / PN / N / NI
4.6. **If N/PN to 4.3, 4.4 or 4.5**: Was an appropriate analysis used to estimate the effect of starting and adhering to the intervention?		NA / Y / PY / PN / N / NI
**Risk of bias judgement**		Low / Moderate / Serious / Critical / NI
Optional: What is the predicted direction of bias due to deviations from the intended interventions?		Favours experimental / Favours comparator / Towards null /Away from null / Unpredictable

Bias due to missing data		
5.1 Were outcome data available for all, or nearly all, participants?		Y / PY / PN / N / NI
5.2 Were participants excluded due to missing data on intervention status?		Y / PY / PN / N / NI
5.3 Were participants excluded due to missing data on other variables needed for the analysis?		Y / PY / PN / N / NI
5.4 **If PN/N to 5.1, or Y/PY to 5.2 or 5.3**: Are the proportion of participants and reasons for missing data similar across interventions?		NA / Y / PY / PN / N / NI
5.5 **If PN/N to 5.1, or Y/PY to 5.2 or 5.3**: Is there evidence that results were robust to the presence of missing data?		NA / Y / PY / PN / N / NI
**Risk of bias judgement**		Low / Moderate / Serious / Critical / NI
Optional: What is the predicted direction of bias due to missing data?		Favours experimental / Favours comparator / Towards null /Away from null / Unpredictable

Bias in measurement of outcomes		
6.1 Could the outcome measure have been influenced by knowledge of the intervention received?		Y / PY / PN / N / NI
6.2 Were outcome assessors aware of the intervention received by study participants?		Y / PY / PN / N / NI
6.3 Were the methods of outcome assessment comparable across intervention groups?		Y / PY / PN / N / NI
6.4 Were any systematic errors in measurement of the outcome related to intervention received?		Y / PY / PN / N / NI
**Risk of bias judgement**		Low / Moderate / Serious / Critical / NI
Optional: What is the predicted direction of bias due to measurement of outcomes?		Favours experimental / Favours comparator / Towards null /Away from null / Unpredictable

Bias in selection of the reported result		
Is the reported effect estimate likely to be selected, on the basis of the results, from...<br>7.1. ... multiple outcome *measurements* within the outcome domain?		Y / PY / PN / N / NI
7.2 ... multiple *analyses* of the intervention-outcome relationship?		Y / PY / PN / N / NI
7.3 ... different *subgroups*?		Y / PY / PN / N / NI
**Risk of bias judgement**		Low / Moderate / Serious / Critical / NI
Optional: What is the predicted direction of bias due to selection of the reported result?		Favours experimental / Favours comparator / Towards null /Away from null / Unpredictable

Overall bias		
**Risk of bias judgement**		Low / Moderate / Serious / Critical / NI
Optional: What is the overall predicted direction of bias for this outcome?		Favours experimental / Favours comparator / Towards null /Away from null / Unpredictable

This work is licensed under a Creative Commons Attribution-NonCommercial-NoDerivatives 4.0 International License.

**Domain**	**Recurrence**	**Residual**
Bias due to confounding	Serious risk	Serious risk
Bias in selection of participants into the study	Low risk	Low risk
Bias in classification of interventions	Low risk	Low risk
Bias due to deviations from intended interventions	Low risk	Low risk
Bias due to missing data	Low risk	Low risk
Bias in measurement of outcomes	Low risk	Low risk
Bias in selection of the reported result	Moderate risk	Moderate risk
**Overall**	**Serious risk**	**Serious risk**

Tan 2019

Specify a target randomized trial specific to the study

Design	Individually randomized / Cluster randomized / Matched (e.g. cross-over)
Participants	Patients with histopathological diagnosis of AIS after cervical excision
Experimental intervention	Conservative treatment
Comparator	Demolitive treatment (hysterectomy)

Is your aim for this study…?

- ■ to assess the effect of *assignment to* intervention
- ☐ to assess the effect of *starting and adhering to* intervention

Specify the outcome

Specify which outcome is being assessed for risk of bias (typically from among those earmarked for the Summary of Findings table). Specify whether this is a proposed benefit or harm of intervention.

Recurrence

Specify the numerical result being assessed

In case of multiple alternative analyses being presented, specify the numeric result (e.g. RR = 1.52 (95% CI 0.83 to 2.77) and/or a reference (e.g. to a table, figure or paragraph) that uniquely defines the result being assessed.

Preliminary consideration of confounders

Complete a row for each important confounding domain (i) listed in the review protocol; and (ii) relevant to the setting of this particular study, or which the study authors identified as potentially important.

"Important" confounding domains are those for which, in the context of this study, adjustment is expected to lead to a clinically important change in the estimated effect of the intervention. "Validity" refers to whether the confounding variable or variables fully measure the domain, while "reliability" refers to the precision of the measurement (more measurement error means less reliability).

**(i) Confounding domains listed in the review protocol**				
Confounding domain	Measured variable(s)	Is there evidence that controlling for this variable was unnecessary?*	Is the confounding domain measured validly and reliably by this variable (or these variables)?	OPTIONAL: Is failure to adjust for this variable (alone) expected to favour the experimental intervention or the comparator?
Age	Years	No	Yes	-

**(ii) Additional confounding domains relevant to the setting of this particular study, or which the study authors identified as important**				
Confounding domain	Measured variable(s)	Is there evidence that controlling for this variable was unnecessary?*	Is the confounding domain measured validly and reliably by this variable (or these variables)?	OPTIONAL: Is failure to adjust for this variable (alone) expected to favour the experimental intervention or the comparator?
Depth of specimen excised	Depth (mm)	No	Yes	-
Excisional margin status	Positive / Negative	No	Yes	-

* In the context of a particular study, variables can be demonstrated not to be confounders and so not included in the analysis: (a) if they are not predictive of the outcome; (b) if they are not predictive of intervention; or (c) because adjustment makes no or minimal difference to the estimated effect of the primary parameter. Note that "no statistically significant association" is not the same as "not predictive".

Preliminary consideration of co-interventions

Complete a row for each important co-intervention (i) listed in the review protocol; and (ii) relevant to the setting of this particular study, or which the study authors identified as important.

"Important" co-interventions are those for which, in the context of this study, adjustment is expected to lead to a clinically important change in the estimated effect of the intervention.

**(i) Co-interventions listed in the review protocol**		
Co-intervention	Is there evidence that controlling for this co-intervention was unnecessary (e.g. because it was not administered)?	Is presence of this co-intervention likely to favour outcomes in the experimental intervention or the comparator
None	-	-

**(ii) Additional co-interventions relevant to the setting of this particular study, or which the study authors identified as important**		
Co-intervention	Is there evidence that controlling for this co-intervention was unnecessary (e.g. because it was not administered)?	Is presence of this co-intervention likely to favour outcomes in the experimental intervention or the comparator
None	-	-

Risk of bias assessment

Responses underlined in green are potential markers for low risk of bias, and responses in red are potential markers for a risk of bias. Where questions relate only to sign posts to other questions, no formatting is used.

Signalling questions	Description	Response options
**Bias due to confounding**		
1.1 Is there potential for confounding of the effect of intervention in this study?<br>**If N/PN to 1.1:** the study can be considered to be at low risk of bias due to confounding and no further signalling questions need be considered		Y / PY / PN / N
**If Y/PY to 1.1**: determine whether there is a need to assess time-varying confounding:		
1.2. Was the analysis based on splitting participants' follow up time according to intervention received?<br>**If N/PN**, answer questions relating to baseline confounding (1.4 to 1.6)<br>**If Y/PY**, go to question 1.3.		NA / Y / PY / PN / N / NI
1.3. Were intervention discontinuations or switches likely to be related to factors that are prognostic for the outcome?<br>**If N/PN**, answer questions relating to baseline confounding (1.4 to 1.6)<br>**If Y/PY**, answer questions relating to both baseline and time-varying confounding (1.7 and 1.8)		NA / Y / PY / PN / N / NI

**Questions relating to baseline confounding only**		
1.4. Did the authors use an appropriate analysis method that controlled for all the important confounding domains?		NA / Y / PY / PN / N / NI
1.5. **If Y/PY to 1.4**: Were confounding domains that were controlled for measured validly and reliably by the variables available in this study?		NA / Y / PY / PN / N / NI
1.6. Did the authors control for any post-intervention variables that could have been affected by the intervention?		NA / Y / PY / PN / N / NI
**Questions relating to baseline and time-varying confounding**		
1.7. Did the authors use an appropriate analysis method that controlled for all the important confounding domains and for time-varying confounding?		NA / Y / PY / PN / N / NI
1.8. **If Y/PY to 1.7**: Were confounding domains that were controlled for measured validly and reliably by the variables available in this study?		NA / Y / PY / PN / N / NI
**Risk of bias judgement**		Low / Moderate / Serious / Critical / NI
Optional: What is the predicted direction of bias due to confounding?		Favours experimental / Favours comparator / Unpredictable

**Bias in selection of participants into the study**		
2.1. Was selection of participants into the study (or into the analysis) based on participant characteristics observed after the start of intervention?<br>**If N/PN to 2.1:** go to 2.4		Y / PY / PN / N / NI
2.2. **If Y/PY to 2.1**: Were the post-intervention variables that influenced selection likely to be associated with intervention?		NA / Y / PY / PN / N / NI
2.3 **If Y/PY to 2.2**: Were the post-intervention variables that influenced selection likely to be influenced by the outcome or a cause of the outcome?		NA / Y / PY / PN / N / NI
2.4. Do start of follow-up and start of intervention coincide for most participants?		Y / PY / PN / N / NI
2.5. **If Y/PY to 2.2 and 2.3, or N/PN to 2.4**: Were adjustment techniques used that are likely to correct for the presence of selection biases?		NA / Y / PY / PN / N / NI
**Risk of bias judgement**		Low / Moderate / Serious / Critical / NI
Optional: What is the predicted direction of bias due to selection of participants into the study?		Favours experimental / Favours comparator / Towards null /Away from null / Unpredictable

Bias in classification of interventions		
3.1 Were intervention groups clearly defined?		Y / PY / PN / N / NI
3.2 Was the information used to define intervention groups recorded at the start of the intervention?		Y / PY / PN / N / NI
3.3 Could classification of intervention status have been affected by knowledge of the outcome or risk of the outcome?		Y / PY / PN / N / NI
**Risk of bias judgement**		Low / Moderate / Serious / Critical / NI
Optional: What is the predicted direction of bias due to classification of interventions?		Favours experimental / Favours comparator / Towards null /Away from null / Unpredictable

Bias due to deviations from intended interventions		
**If your aim for this study is to assess the effect of assignment to intervention, answer questions 4.1 and 4.2**		
4.1. Were there deviations from the intended intervention beyond what would be expected in usual practice?		Y / PY / PN / N / NI
4.2. **If Y/PY to 4.1**: Were these deviations from intended intervention unbalanced between groups *and* likely to have affected the outcome?		NA / Y / PY / PN / N / NI
**If your aim for this study is to assess the effect of starting and adhering to intervention, answer questions 4.3 to 4.6**		
4.3. Were important co-interventions balanced across intervention groups?		Y / PY / PN / N / NI
4.4. Was the intervention implemented successfully for most participants?		Y / PY / PN / N / NI
4.5. Did study participants adhere to the assigned intervention regimen?		Y / PY / PN / N / NI
4.6. **If N/PN to 4.3, 4.4 or 4.5**: Was an appropriate analysis used to estimate the effect of starting and adhering to the intervention?		NA / Y / PY / PN / N / NI
**Risk of bias judgement**		Low / Moderate / Serious / Critical / NI
Optional: What is the predicted direction of bias due to deviations from the intended interventions?		Favours experimental / Favours comparator / Towards null /Away from null / Unpredictable

Bias due to missing data		
5.1 Were outcome data available for all, or nearly all, participants?		Y / PY / PN / N / NI
5.2 Were participants excluded due to missing data on intervention status?		Y / PY / PN / N / NI
5.3 Were participants excluded due to missing data on other variables needed for the analysis?		Y / PY / PN / N / NI
5.4 **If PN/N to 5.1, or Y/PY to 5.2 or 5.3**: Are the proportion of participants and reasons for missing data similar across interventions?		NA / Y / PY / PN / N / NI
5.5 **If PN/N to 5.1, or Y/PY to 5.2 or 5.3**: Is there evidence that results were robust to the presence of missing data?		NA / Y / PY / PN / N / NI
**Risk of bias judgement**		Low / Moderate / Serious / Critical / NI
Optional: What is the predicted direction of bias due to missing data?		Favours experimental / Favours comparator / Towards null /Away from null / Unpredictable

Bias in measurement of outcomes		
6.1 Could the outcome measure have been influenced by knowledge of the intervention received?		Y / PY / PN / N / NI
6.2 Were outcome assessors aware of the intervention received by study participants?		Y / PY / PN / N / NI
6.3 Were the methods of outcome assessment comparable across intervention groups?		Y / PY / PN / N / NI
6.4 Were any systematic errors in measurement of the outcome related to intervention received?		Y / PY / PN / N / NI
**Risk of bias judgement**		Low / Moderate / Serious / Critical / NI
Optional: What is the predicted direction of bias due to measurement of outcomes?		Favours experimental / Favours comparator / Towards null /Away from null / Unpredictable

Bias in selection of the reported result		
Is the reported effect estimate likely to be selected, on the basis of the results, from...<br>7.1. ... multiple outcome *measurements* within the outcome domain?		Y / PY / PN / N / NI
7.2 ... multiple *analyses* of the intervention-outcome relationship?		Y / PY / PN / N / NI
7.3 ... different *subgroups*?		Y / PY / PN / N / NI
**Risk of bias judgement**		Low / Moderate / Serious / Critical / NI
Optional: What is the predicted direction of bias due to selection of the reported result?		Favours experimental / Favours comparator / Towards null /Away from null / Unpredictable

Overall bias		
**Risk of bias judgement**		Low / Moderate / Serious / Critical / NI
Optional: What is the overall predicted direction of bias for this outcome?		Favours experimental / Favours comparator / Towards null /Away from null / Unpredictable

This work is licensed under a Creative Commons Attribution-NonCommercial-NoDerivatives 4.0 International License.

**Domain**	**Recurrence**
Bias due to confounding	Moderate risk
Bias in selection of participants into the study	Low risk
Bias in classification of interventions	Low risk
Bias due to deviations from intended interventions	Low risk
Bias due to missing data	Low risk
Bias in measurement of outcomes	Low risk
Bias in selection of the reported result	Moderate risk
**Overall**	**Moderate risk**

Tay 1999

Specify a target randomized trial specific to the study

Design	Individually randomized / Cluster randomized / Matched (e.g. cross-over)
Participants	Patients with histopathological diagnosis of AIS after cervical excision
Experimental intervention	Conservative treatment
Comparator	Demolitive treatment (hysterectomy)

Is your aim for this study…?

- ■ to assess the effect of *assignment to* intervention
- ☐ to assess the effect of *starting and adhering to* intervention

Specify the outcome

Specify which outcome is being assessed for risk of bias (typically from among those earmarked for the Summary of Findings table). Specify whether this is a proposed benefit or harm of intervention.

Residual

Specify the numerical result being assessed

In case of multiple alternative analyses being presented, specify the numeric result (e.g. RR = 1.52 (95% CI 0.83 to 2.77) and/or a reference (e.g. to a table, figure or paragraph) that uniquely defines the result being assessed.

Preliminary consideration of confounders

Complete a row for each important confounding domain (i) listed in the review protocol; and (ii) relevant to the setting of this particular study, or which the study authors identified as potentially important.

"Important" confounding domains are those for which, in the context of this study, adjustment is expected to lead to a clinically important change in the estimated effect of the intervention. "Validity" refers to whether the confounding variable or variables fully measure the domain, while "reliability" refers to the precision of the measurement (more measurement error means less reliability).

**(i) Confounding domains listed in the review protocol**				
Confounding domain	Measured variable(s)	Is there evidence that controlling for this variable was unnecessary?*	Is the confounding domain measured validly and reliably by this variable (or these variables)?	OPTIONAL: Is failure to adjust for this variable (alone) expected to favour the experimental intervention or the comparator?
Age	Not evaluated as confounder	No	-	-

**(ii) Additional confounding domains relevant to the setting of this particular study, or which the study authors identified as important**				
Confounding domain	Measured variable(s)	Is there evidence that controlling for this variable was unnecessary?*	Is the confounding domain measured validly and reliably by this variable (or these variables)?	OPTIONAL: Is failure to adjust for this variable (alone) expected to favour the experimental intervention or the comparator?
-	-	-	-	-

* In the context of a particular study, variables can be demonstrated not to be confounders and so not included in the analysis: (a) if they are not predictive of the outcome; (b) if they are not predictive of intervention; or (c) because adjustment makes no or minimal difference to the estimated effect of the primary parameter. Note that "no statistically significant association" is not the same as "not predictive".

Preliminary consideration of co-interventions

Complete a row for each important co-intervention (i) listed in the review protocol; and (ii) relevant to the setting of this particular study, or which the study authors identified as important.

"Important" co-interventions are those for which, in the context of this study, adjustment is expected to lead to a clinically important change in the estimated effect of the intervention.

**(i) Co-interventions listed in the review protocol**		
Co-intervention	Is there evidence that controlling for this co-intervention was unnecessary (e.g. because it was not administered)?	Is presence of this co-intervention likely to favour outcomes in the experimental intervention or the comparator
None	-	-

**(ii) Additional co-interventions relevant to the setting of this particular study, or which the study authors identified as important**		
Co-intervention	Is there evidence that controlling for this co-intervention was unnecessary (e.g. because it was not administered)?	Is presence of this co-intervention likely to favour outcomes in the experimental intervention or the comparator
None	-	-

Risk of bias assessment

Responses underlined in green are potential markers for low risk of bias, and responses in red are potential markers for a risk of bias. Where questions relate only to sign posts to other questions, no formatting is used.

Signalling questions	Description	Response options
**Bias due to confounding**		
1.1 Is there potential for confounding of the effect of intervention in this study?<br>**If N/PN to 1.1:** the study can be considered to be at low risk of bias due to confounding and no further signalling questions need be considered		Y / PY / PN / N
**If Y/PY to 1.1**: determine whether there is a need to assess time-varying confounding:		
1.2. Was the analysis based on splitting participants' follow up time according to intervention received?<br>**If N/PN**, answer questions relating to baseline confounding (1.4 to 1.6)<br>**If Y/PY**, go to question 1.3.		NA / Y / PY / PN / N / NI
1.3. Were intervention discontinuations or switches likely to be related to factors that are prognostic for the outcome?<br>**If N/PN**, answer questions relating to baseline confounding (1.4 to 1.6)<br>**If Y/PY**, answer questions relating to both baseline and time-varying confounding (1.7 and 1.8)		NA / Y / PY / PN / N / NI

**Questions relating to baseline confounding only**		
1.4. Did the authors use an appropriate analysis method that controlled for all the important confounding domains?		NA / Y / PY / PN / N / NI
1.5. **If Y/PY to 1.4**: Were confounding domains that were controlled for measured validly and reliably by the variables available in this study?		NA / Y / PY / PN / N / NI
1.6. Did the authors control for any post-intervention variables that could have been affected by the intervention?		NA / Y / PY / PN / N / NI
**Questions relating to baseline and time-varying confounding**		
1.7. Did the authors use an appropriate analysis method that controlled for all the important confounding domains and for time-varying confounding?		NA / Y / PY / PN / N / NI
1.8. **If Y/PY to 1.7**: Were confounding domains that were controlled for measured validly and reliably by the variables available in this study?		NA / Y / PY / PN / N / NI
**Risk of bias judgement**		Low / Moderate / Serious / Critical / NI
Optional: What is the predicted direction of bias due to confounding?		Favours experimental / Favours comparator / Unpredictable

Bias in selection of participants into the study		
2.1. Was selection of participants into the study (or into the analysis) based on participant characteristics observed after the start of intervention?<br>**If N/PN to 2.1:** go to 2.4		Y / PY / PN / N / NI
2.2. **If Y/PY to 2.1**: Were the post-intervention variables that influenced selection likely to be associated with intervention?		NA / Y / PY / PN / N / NI
2.3 **If Y/PY to 2.2**: Were the post-intervention variables that influenced selection likely to be influenced by the outcome or a cause of the outcome?		NA / Y / PY / PN / N / NI
2.4. Do start of follow-up and start of intervention coincide for most participants?		Y / PY / PN / N / NI
2.5. **If Y/PY to 2.2 and 2.3, or N/PN to 2.4**: Were adjustment techniques used that are likely to correct for the presence of selection biases?		NA / Y / PY / PN / N / NI
**Risk of bias judgement**		Low / Moderate / Serious / Critical / NI
Optional: What is the predicted direction of bias due to selection of participants into the study?		Favours experimental / Favours comparator / Towards null /Away from null / Unpredictable

Bias in classification of interventions		
3.1 Were intervention groups clearly defined?		Y / PY / PN / N / NI
3.2 Was the information used to define intervention groups recorded at the start of the intervention?		Y / PY / PN / N / NI
3.3 Could classification of intervention status have been affected by knowledge of the outcome or risk of the outcome?		Y / PY / PN / N / NI
**Risk of bias judgement**		Low / Moderate / Serious / Critical / NI
Optional: What is the predicted direction of bias due to classification of interventions?		Favours experimental / Favours comparator / Towards null /Away from null / Unpredictable

Bias due to deviations from intended interventions		
**If your aim for this study is to assess the effect of assignment to intervention, answer questions 4.1 and 4.2**		
4.1. Were there deviations from the intended intervention beyond what would be expected in usual practice?		Y / PY / PN / N / NI
4.2. **If Y/PY to 4.1**: Were these deviations from intended intervention unbalanced between groups *and* likely to have affected the outcome?		NA / Y / PY / PN / N / NI
**If your aim for this study is to assess the effect of starting and adhering to intervention, answer questions 4.3 to 4.6**		
4.3. Were important co-interventions balanced across intervention groups?		Y / PY / PN / N / NI
4.4. Was the intervention implemented successfully for most participants?		Y / PY / PN / N / NI
4.5. Did study participants adhere to the assigned intervention regimen?		Y / PY / PN / N / NI
4.6. **If N/PN to 4.3, 4.4 or 4.5**: Was an appropriate analysis used to estimate the effect of starting and adhering to the intervention?		NA / Y / PY / PN / N / NI
**Risk of bias judgement**		Low / Moderate / Serious / Critical / NI
Optional: What is the predicted direction of bias due to deviations from the intended interventions?		Favours experimental / Favours comparator / Towards null /Away from null / Unpredictable

Bias due to missing data		
5.1 Were outcome data available for all, or nearly all, participants?		Y / PY / PN / N / NI
5.2 Were participants excluded due to missing data on intervention status?		Y / PY / PN / N / NI
5.3 Were participants excluded due to missing data on other variables needed for the analysis?		Y / PY / PN / N / NI
5.4 **If PN/N to 5.1, or Y/PY to 5.2 or 5.3**: Are the proportion of participants and reasons for missing data similar across interventions?		NA / Y / PY / PN / N / NI
5.5 **If PN/N to 5.1, or Y/PY to 5.2 or 5.3**: Is there evidence that results were robust to the presence of missing data?		NA / Y / PY / PN / N / NI
**Risk of bias judgement**		Low / Moderate / Serious / Critical / NI
Optional: What is the predicted direction of bias due to missing data?		Favours experimental / Favours comparator / Towards null /Away from null / Unpredictable

Bias in measurement of outcomes		
6.1 Could the outcome measure have been influenced by knowledge of the intervention received?		Y / PY / PN / N / NI
6.2 Were outcome assessors aware of the intervention received by study participants?		Y / PY / PN / N / NI
6.3 Were the methods of outcome assessment comparable across intervention groups?		Y / PY / PN / N / NI
6.4 Were any systematic errors in measurement of the outcome related to intervention received?		Y / PY / PN / N / NI
**Risk of bias judgement**		Low / Moderate / Serious / Critical / NI
Optional: What is the predicted direction of bias due to measurement of outcomes?		Favours experimental / Favours comparator / Towards null /Away from null / Unpredictable

Bias in selection of the reported result		
Is the reported effect estimate likely to be selected, on the basis of the results, from...		
7.1. ... multiple outcome *measurements* within the outcome domain?		Y / PY / PN / N / NI
7.2 ... multiple *analyses* of the intervention-outcome relationship?		Y / PY / PN / N / NI
7.3 ... different *subgroups*?		Y / PY / PN / N / NI
**Risk of bias judgement**		Low / Moderate / Serious / Critical / NI
Optional: What is the predicted direction of bias due to selection of the reported result?		Favours experimental / Favours comparator / Towards null /Away from null / Unpredictable

Overall bias		
**Risk of bias judgement**		Low / Moderate / Serious / Critical / NI
Optional: What is the overall predicted direction of bias for this outcome?		Favours experimental / Favours comparator / Towards null /Away from null / Unpredictable

This work is licensed under a Creative Commons Attribution-NonCommercial-NoDerivatives 4.0 International License.

**Domain**	**Recurrence**
Bias due to confounding	Serious risk
Bias in selection of participants into the study	Low risk
Bias in classification of interventions	Low risk
Bias due to deviations from intended interventions	Low risk
Bias due to missing data	Low risk
Bias in measurement of outcomes	Low risk
Bias in selection of the reported result	Moderate risk
**Overall**	**Serious risk**

Taylor 2014

Specify a target randomized trial specific to the study

Design	Individually randomized / Cluster randomized / Matched (e.g. cross-over)
Participants	Patients with histopathological diagnosis of AIS after cervical excision
Experimental intervention	Conservative treatment
Comparator	Demolitive treatment (hysterectomy)

Is your aim for this study…?

- ■ to assess the effect of *assignment to* intervention
- ☐ to assess the effect of *starting and adhering to* intervention

Specify the outcome

Specify which outcome is being assessed for risk of bias (typically from among those earmarked for the Summary of Findings table). Specify whether this is a proposed benefit or harm of intervention.

Recurrence<br>Residual

Specify the numerical result being assessed

In case of multiple alternative analyses being presented, specify the numeric result (e.g. RR = 1.52 (95% CI 0.83 to 2.77) and/or a reference (e.g. to a table, figure or paragraph) that uniquely defines the result being assessed.

Preliminary consideration of confounders

Complete a row for each important confounding domain (i) listed in the review protocol; and (ii) relevant to the setting of this particular study, or which the study authors identified as potentially important.

"Important" confounding domains are those for which, in the context of this study, adjustment is expected to lead to a clinically important change in the estimated effect of the intervention. "Validity" refers to whether the confounding variable or variables fully measure the domain, while "reliability" refers to the precision of the measurement (more measurement error means less reliability).

**(i) Confounding domains listed in the review protocol**				
Confounding domain	Measured variable(s)	Is there evidence that controlling for this variable was unnecessary?*	Is the confounding domain measured validly and reliably by this variable (or these variables)?	OPTIONAL: Is failure to adjust for this variable (alone) expected to favour the experimental intervention or the comparator?
Age	Not evaluated as confounder	No	-	-

**(ii) Additional confounding domains relevant to the setting of this particular study, or which the study authors identified as important**				
Confounding domain	Measured variable(s)	Is there evidence that controlling for this variable was unnecessary?*	Is the confounding domain measured validly and reliably by this variable (or these variables)?	OPTIONAL: Is failure to adjust for this variable (alone) expected to favour the experimental intervention or the comparator?
-	-	-	-	-

* In the context of a particular study, variables can be demonstrated not to be confounders and so not included in the analysis: (a) if they are not predictive of the outcome; (b) if they are not predictive of intervention; or (c) because adjustment makes no or minimal difference to the estimated effect of the primary parameter. Note that "no statistically significant association" is not the same as "not predictive".

Preliminary consideration of co-interventions

Complete a row for each important co-intervention (i) listed in the review protocol; and (ii) relevant to the setting of this particular study, or which the study authors identified as important.

"Important" co-interventions are those for which, in the context of this study, adjustment is expected to lead to a clinically important change in the estimated effect of the intervention.

**(i) Co-interventions listed in the review protocol**		
Co-intervention	Is there evidence that controlling for this co-intervention was unnecessary (e.g. because it was not administered)?	Is presence of this co-intervention likely to favour outcomes in the experimental intervention or the comparator
None	-	-

**(ii) Additional co-interventions relevant to the setting of this particular study, or which the study authors identified as important**		
Co-intervention	Is there evidence that controlling for this co-intervention was unnecessary (e.g. because it was not administered)?	Is presence of this co-intervention likely to favour outcomes in the experimental intervention or the comparator
None	-	-

Risk of bias assessment

Responses underlined in green are potential markers for low risk of bias, and responses in red are potential markers for a risk of bias. Where questions relate only to sign posts to other questions, no formatting is used.

Signalling questions	Description	Response options
**Bias due to confounding**		
1.1 Is there potential for confounding of the effect of intervention in this study?<br>**If N/PN to 1.1:** the study can be considered to be at low risk of bias due to confounding and no further signalling questions need be considered		Y / PY / PN / N
**If Y/PY to 1.1**: determine whether there is a need to assess time-varying confounding:		
1.2. Was the analysis based on splitting participants' follow up time according to intervention received?<br>**If N/PN**, answer questions relating to baseline confounding (1.4 to 1.6)<br>**If Y/PY**, go to question 1.3.		NA / Y / PY / PN / N / NI
1.3. Were intervention discontinuations or switches likely to be related to factors that are prognostic for the outcome?<br>**If N/PN**, answer questions relating to baseline confounding (1.4 to 1.6)<br>**If Y/PY**, answer questions relating to both baseline and time-varying confounding (1.7 and 1.8)		NA / Y / PY / PN / N / NI

**Questions relating to baseline confounding only**		
1.4. Did the authors use an appropriate analysis method that controlled for all the important confounding domains?		NA / Y / PY / PN / N / NI
1.5. **If Y/PY to 1.4**: Were confounding domains that were controlled for measured validly and reliably by the variables available in this study?		NA / Y / PY / PN / N / NI
1.6. Did the authors control for any post-intervention variables that could have been affected by the intervention?		NA / Y / PY / PN / N / NI
**Questions relating to baseline and time-varying confounding**		
1.7. Did the authors use an appropriate analysis method that controlled for all the important confounding domains and for time-varying confounding?		NA / Y / PY / PN / N / NI
1.8. **If Y/PY to 1.7**: Were confounding domains that were controlled for measured validly and reliably by the variables available in this study?		NA / Y / PY / PN / N / NI
**Risk of bias judgement**		Low / Moderate / Serious / Critical / NI
Optional: What is the predicted direction of bias due to confounding?		Favours experimental / Favours comparator / Unpredictable

Bias in selection of participants into the study		
2.1. Was selection of participants into the study (or into the analysis) based on participant characteristics observed after the start of intervention?<br>**If N/PN to 2.1:** go to 2.4		Y / PY / PN / N / NI
2.2. **If Y/PY to 2.1**: Were the post-intervention variables that influenced selection likely to be associated with intervention?		NA / Y / PY / PN / N / NI
2.3 **If Y/PY to 2.2**: Were the post-intervention variables that influenced selection likely to be influenced by the outcome or a cause of the outcome?		NA / Y / PY / PN / N / NI
2.4. Do start of follow-up and start of intervention coincide for most participants?		Y / PY / PN / N / NI
2.5. **If Y/PY to 2.2 and 2.3, or N/PN to 2.4**: Were adjustment techniques used that are likely to correct for the presence of selection biases?		NA / Y / PY / PN / N / NI
**Risk of bias judgement**		Low / Moderate / Serious / Critical / NI
Optional: What is the predicted direction of bias due to selection of participants into the study?		Favours experimental / Favours comparator / Towards null /Away from null / Unpredictable

Bias in classification of interventions		
3.1 Were intervention groups clearly defined?		Y / PY / PN / N / NI
3.2 Was the information used to define intervention groups recorded at the start of the intervention?		Y / PY / PN / N / NI
3.3 Could classification of intervention status have been affected by knowledge of the outcome or risk of the outcome?		Y / PY / PN / N / NI
**Risk of bias judgement**		Low / Moderate / Serious / Critical / NI
Optional: What is the predicted direction of bias due to classification of interventions?		Favours experimental / Favours comparator / Towards null /Away from null / Unpredictable

Bias due to deviations from intended interventions		
**If your aim for this study is to assess the effect of assignment to intervention, answer questions 4.1 and 4.2**		
4.1. Were there deviations from the intended intervention beyond what would be expected in usual practice?		Y / PY / PN / N / NI
4.2. **If Y/PY to 4.1**: Were these deviations from intended intervention unbalanced between groups *and* likely to have affected the outcome?		NA / Y / PY / PN / N / NI
**If your aim for this study is to assess the effect of starting and adhering to intervention, answer questions 4.3 to 4.6**		
4.3. Were important co-interventions balanced across intervention groups?		Y / PY / PN / N / NI
4.4. Was the intervention implemented successfully for most participants?		Y / PY / PN / N / NI
4.5. Did study participants adhere to the assigned intervention regimen?		Y / PY / PN / N / NI
4.6. **If N/PN to 4.3, 4.4 or 4.5**: Was an appropriate analysis used to estimate the effect of starting and adhering to the intervention?		NA / Y / PY / PN / N / NI
**Risk of bias judgement**		Low / Moderate / Serious / Critical / NI
Optional: What is the predicted direction of bias due to deviations from the intended interventions?		Favours experimental / Favours comparator / Towards null /Away from null / Unpredictable

Bias due to missing data		
5.1 Were outcome data available for all, or nearly all, participants?		Y / PY / PN / N / NI
5.2 Were participants excluded due to missing data on intervention status?		Y / PY / PN / N / NI
5.3 Were participants excluded due to missing data on other variables needed for the analysis?		Y / PY / PN / N / NI
5.4 **If PN/N to 5.1, or Y/PY to 5.2 or 5.3**: Are the proportion of participants and reasons for missing data similar across interventions?		NA / Y / PY / PN / N / NI
5.5 **If PN/N to 5.1, or Y/PY to 5.2 or 5.3**: Is there evidence that results were robust to the presence of missing data?		NA / Y / PY / PN / N / NI
**Risk of bias judgement**		Low / Moderate / Serious / Critical / NI
Optional: What is the predicted direction of bias due to missing data?		Favours experimental / Favours comparator / Towards null /Away from null / Unpredictable

Bias in measurement of outcomes		
6.1 Could the outcome measure have been influenced by knowledge of the intervention received?		Y / PY / PN / N / NI
6.2 Were outcome assessors aware of the intervention received by study participants?		Y / PY / PN / N / NI
6.3 Were the methods of outcome assessment comparable across intervention groups?		Y / PY / PN / N / NI
6.4 Were any systematic errors in measurement of the outcome related to intervention received?		Y / PY / PN / N / NI
**Risk of bias judgement**		Low / Moderate / Serious / Critical / NI
Optional: What is the predicted direction of bias due to measurement of outcomes?		Favours experimental / Favours comparator / Towards null /Away from null / Unpredictable

Bias in selection of the reported result		
Is the reported effect estimate likely to be selected, on the basis of the results, from... 7.1. ... multiple outcome *measurements* within the outcome domain?		Y / PY / PN / N / NI
7.2 ... multiple *analyses* of the intervention-outcome relationship?		Y / PY / PN / N / NI
7.3 ... different *subgroups*?		Y / PY / PN / N / NI
**Risk of bias judgement**		Low / Moderate / Serious / Critical / NI
Optional: What is the predicted direction of bias due to selection of the reported result?		Favours experimental / Favours comparator / Towards null /Away from null / Unpredictable

Overall bias		
**Risk of bias judgement**		Low / Moderate / Serious / Critical / NI
Optional: What is the overall predicted direction of bias for this outcome?		Favours experimental / Favours comparator / Towards null /Away from null / Unpredictable

This work is licensed under a Creative Commons Attribution-NonCommercial-NoDerivatives 4.0 International License.

**Domain**	**Residual**
Bias due to confounding	Serious risk
Bias in selection of participants into the study	Low risk
Bias in classification of interventions	Low risk
Bias due to deviations from intended interventions	Low risk
Bias due to missing data	Low risk
Bias in measurement of outcomes	Low risk
Bias in selection of the reported result	Moderate risk
**Overall**	**Serious risk**

Wang 2020

Specify a target randomized trial specific to the study

Design	Individually randomized / Cluster randomized / Matched (e.g. cross-over)
Participants	Patients with histopathological diagnosis of AIS after cervical excision
Experimental intervention	Conservative treatment
Comparator	Demolitive treatment (hysterectomy)

Is your aim for this study…?

- ■ to assess the effect of *assignment to* intervention
- ☐ to assess the effect of *starting and adhering to* intervention

Specify the outcome

Specify which outcome is being assessed for risk of bias (typically from among those earmarked for the Summary of Findings table). Specify whether this is a proposed benefit or harm of intervention.

Recurrence

Specify the numerical result being assessed

In case of multiple alternative analyses being presented, specify the numeric result (e.g. RR = 1.52 (95% CI 0.83 to 2.77) and/or a reference (e.g. to a table, figure or paragraph) that uniquely defines the result being assessed.

Preliminary consideration of confounders

Complete a row for each important confounding domain (i) listed in the review protocol; and (ii) relevant to the setting of this particular study, or which the study authors identified as potentially important.

"Important" confounding domains are those for which, in the context of this study, adjustment is expected to lead to a clinically important change in the estimated effect of the intervention. "Validity" refers to whether the confounding variable or variables fully measure the domain, while "reliability" refers to the precision of the measurement (more measurement error means less reliability).

**(i) Confounding domains listed in the review protocol**				
Confounding domain	Measured variable(s)	Is there evidence that controlling for this variable was unnecessary?*	Is the confounding domain measured validly and reliably by this variable (or these variables)?	OPTIONAL: Is failure to adjust for this variable (alone) expected to favour the experimental intervention or the comparator?
Age	Years	No	Yes	-

**(ii) Additional confounding domains relevant to the setting of this particular study, or which the study authors identified as important**				
Confounding domain	Measured variable(s)	Is there evidence that controlling for this variable was unnecessary?*	Is the confounding domain measured validly and reliably by this variable (or these variables)?	OPTIONAL: Is failure to adjust for this variable (alone) expected to favour the experimental intervention or the comparator?
Menopause	Yes/No	No	Yes	-
Margin involvement	Yes/No	No	Yes	-
Hysterectomy removed	Yes/No	No	Yes	-
Glandular involvement	Yes/No	No	Yes	-
Accompany by other lesions	Yes/No	No	Yes	-

* In the context of a particular study, variables can be demonstrated not to be confounders and so not included in the analysis: (a) if they are not predictive of the outcome; (b) if they are not predictive of intervention; or (c) because adjustment makes no or minimal difference to the estimated effect of the primary parameter. Note that "no statistically significant association" is not the same as "not predictive".

Preliminary consideration of co-interventions

Complete a row for each important co-intervention (i) listed in the review protocol; and (ii) relevant to the setting of this particular study, or which the study authors identified as important.

"Important" co-interventions are those for which, in the context of this study, adjustment is expected to lead to a clinically important change in the estimated effect of the intervention.

**(i) Co-interventions listed in the review protocol**		
Co-intervention	Is there evidence that controlling for this co-intervention was unnecessary (e.g. because it was not administered)?	Is presence of this co-intervention likely to favour outcomes in the experimental intervention or the comparator
None	-	-

**(ii) Additional co-interventions relevant to the setting of this particular study, or which the study authors identified as important**		
Co-intervention	Is there evidence that controlling for this co-intervention was unnecessary (e.g. because it was not administered)?	Is presence of this co-intervention likely to favour outcomes in the experimental intervention or the comparator
None	-	-

Risk of bias assessment

Responses underlined in green are potential markers for low risk of bias, and responses in red are potential markers for a risk of bias. Where questions relate only to sign posts to other questions, no formatting is used.

Signalling questions	Description	Response options
**Bias due to confounding**		
1.1 Is there potential for confounding of the effect of intervention in this study?<br>**If N/PN to 1.1:** the study can be considered to be at low risk of bias due to confounding and no further signalling questions need be considered		Y / PY / PN / N
**If Y/PY to 1.1**: determine whether there is a need to assess time-varying confounding:		
1.2. Was the analysis based on splitting participants' follow up time according to intervention received?<br>**If N/PN**, answer questions relating to baseline confounding (1.4 to 1.6)<br>**If Y/PY**, go to question 1.3.		NA / Y / PY / PN / N / NI
1.3. Were intervention discontinuations or switches likely to be related to factors that are prognostic for the outcome?<br>**If N/PN**, answer questions relating to baseline confounding (1.4 to 1.6)<br>**If Y/PY**, answer questions relating to both baseline and time-varying confounding (1.7 and 1.8)		NA / Y / PY / PN / N / NI

**Questions relating to baseline confounding only**		
1.4. Did the authors use an appropriate analysis method that controlled for all the important confounding domains?		NA / Y / PY / PN / N / NI
1.5. **If Y/PY to 1.4**: Were confounding domains that were controlled for measured validly and reliably by the variables available in this study?		NA / Y / PY / PN / N / NI
1.6. Did the authors control for any post-intervention variables that could have been affected by the intervention?		NA / Y / PY / PN / N / NI
**Questions relating to baseline and time-varying confounding**		
1.7. Did the authors use an appropriate analysis method that controlled for all the important confounding domains and for time-varying confounding?		NA / Y / PY / PN / N / NI
1.8. **If Y/PY to 1.7**: Were confounding domains that were controlled for measured validly and reliably by the variables available in this study?		NA / Y / PY / PN / N / NI
**Risk of bias judgement**		Low / Moderate / Serious / Critical / NI
Optional: What is the predicted direction of bias due to confounding?		Favours experimental / Favours comparator / Unpredictable

Bias in selection of participants into the study		
2.1. Was selection of participants into the study (or into the analysis) based on participant characteristics observed after the start of intervention?<br>**If N/PN to 2.1:** go to 2.4<br>2.2. **If Y/PY to 2.1**: Were the post-intervention variables that influenced selection likely to be associated with intervention?<br>2.3 **If Y/PY to 2.2**: Were the post-intervention variables that influenced selection likely to be influenced by the outcome or a cause of the outcome?		Y / PY / PN / N / NI<br><br>NA / Y / PY / PN / N / NI<br><br>NA / Y / PY / PN / N / NI
2.4. Do start of follow-up and start of intervention coincide for most participants?		Y / PY / PN / N / NI
2.5. **If Y/PY to 2.2 and 2.3, or N/PN to 2.4**: Were adjustment techniques used that are likely to correct for the presence of selection biases?		NA / Y / PY / PN / N / NI
**Risk of bias judgement**		Low / Moderate / Serious / Critical / NI
Optional: What is the predicted direction of bias due to selection of participants into the study?		Favours experimental / Favours comparator / Towards null /Away from null / Unpredictable

Bias in classification of interventions		
3.1 Were intervention groups clearly defined?		Y / PY / PN / N / NI
3.2 Was the information used to define intervention groups recorded at the start of the intervention?		Y / PY / PN / N / NI
3.3 Could classification of intervention status have been affected by knowledge of the outcome or risk of the outcome?		Y / PY / PN / N / NI
**Risk of bias judgement**		Low / Moderate / Serious / Critical / NI
Optional: What is the predicted direction of bias due to classification of interventions?		Favours experimental / Favours comparator / Towards null /Away from null / Unpredictable

Bias due to deviations from intended interventions		
**If your aim for this study is to assess the effect of assignment to intervention, answer questions 4.1 and 4.2**		
4.1. Were there deviations from the intended intervention beyond what would be expected in usual practice?		Y / PY / PN / N / NI
4.2. **If Y/PY to 4.1**: Were these deviations from intended intervention unbalanced between groups *and* likely to have affected the outcome?		NA / Y / PY / PN / N / NI
**If your aim for this study is to assess the effect of starting and adhering to intervention, answer questions 4.3 to 4.6**		
4.3. Were important co-interventions balanced across intervention groups?		Y / PY / PN / N / NI
4.4. Was the intervention implemented successfully for most participants?		Y / PY / PN / N / NI
4.5. Did study participants adhere to the assigned intervention regimen?		Y / PY / PN / N / NI
4.6. **If N/PN to 4.3, 4.4 or 4.5**: Was an appropriate analysis used to estimate the effect of starting and adhering to the intervention?		NA / Y / PY / PN / N / NI
**Risk of bias judgement**		Low / Moderate / Serious / Critical / NI
Optional: What is the predicted direction of bias due to deviations from the intended interventions?		Favours experimental / Favours comparator / Towards null /Away from null / Unpredictable

Bias due to missing data		
5.1 Were outcome data available for all, or nearly all, participants?		Y / PY / PN / N / NI
5.2 Were participants excluded due to missing data on intervention status?		Y / PY / PN / N / NI
5.3 Were participants excluded due to missing data on other variables needed for the analysis?		Y / PY / PN / N / NI
5.4 **If PN/N to 5.1, or Y/PY to 5.2 or 5.3**: Are the proportion of participants and reasons for missing data similar across interventions?		NA / Y / PY / PN / N / NI
5.5 **If PN/N to 5.1, or Y/PY to 5.2 or 5.3**: Is there evidence that results were robust to the presence of missing data?		NA / Y / PY / PN / N / NI
**Risk of bias judgement**		Low / Moderate / Serious / Critical / NI
Optional: What is the predicted direction of bias due to missing data?		Favours experimental / Favours comparator / Towards null /Away from null / Unpredictable

Bias in measurement of outcomes		
6.1 Could the outcome measure have been influenced by knowledge of the intervention received?		Y / PY / PN / N / NI
6.2 Were outcome assessors aware of the intervention received by study participants?		Y / PY / PN / N / NI
6.3 Were the methods of outcome assessment comparable across intervention groups?		Y / PY / PN / N / NI
6.4 Were any systematic errors in measurement of the outcome related to intervention received?		Y / PY / PN / N / NI
**Risk of bias judgement**		Low / Moderate / Serious / Critical / NI
Optional: What is the predicted direction of bias due to measurement of outcomes?		Favours experimental / Favours comparator / Towards null /Away from null / Unpredictable

Bias in selection of the reported result		
Is the reported effect estimate likely to be selected, on the basis of the results, from...<br>7.1. ... multiple outcome *measurements* within the outcome domain?		Y / PY / PN / N / NI
7.2 ... multiple *analyses* of the intervention-outcome relationship?		Y / PY / PN / N / NI
7.3 ... different *subgroups*?		Y / PY / PN / N / NI
**Risk of bias judgement**		Low / Moderate / Serious / Critical / NI
Optional: What is the predicted direction of bias due to selection of the reported result?		Favours experimental / Favours comparator / Towards null /Away from null / Unpredictable

Overall bias		
**Risk of bias judgement**		Low / Moderate / Serious / Critical / NI
Optional: What is the overall predicted direction of bias for this outcome?		Favours experimental / Favours comparator / Towards null /Away from null / Unpredictable

This work is licensed under a Creative Commons Attribution-NonCommercial-NoDerivatives 4.0 International License.

**Domain**	**Recurrence**
Bias due to confounding	Moderate risk
Bias in selection of participants into the study	Low risk
Bias in classification of interventions	Low risk
Bias due to deviations from intended interventions	Low risk
Bias due to missing data	Low risk
Bias in measurement of outcomes	Low risk
Bias in selection of the reported result	Moderate risk
**Overall**	**Moderate risk**

Wolf 1997

Specify a target randomized trial specific to the study

Design	Individually randomized / Cluster randomized / Matched (e.g. cross-over)
Participants	Patients with histopathological diagnosis of AIS after cervical excision
Experimental intervention	Conservative treatment
Comparator	Demolitive treatment (hysterectomy)

Is your aim for this study…?

- ■ to assess the effect of *assignment to* intervention
- ☐ to assess the effect of *starting and adhering to* intervention

Specify the outcome

Specify which outcome is being assessed for risk of bias (typically from among those earmarked for the Summary of Findings table). Specify whether this is a proposed benefit or harm of intervention.

Recurrence<br>Residual

Specify the numerical result being assessed

In case of multiple alternative analyses being presented, specify the numeric result (e.g. RR = 1.52 (95% CI 0.83 to 2.77) and/or a reference (e.g. to a table, figure or paragraph) that uniquely defines the result being assessed.

Preliminary consideration of confounders

Complete a row for each important confounding domain (i) listed in the review protocol; and (ii) relevant to the setting of this particular study, or which the study authors identified as potentially important.

"Important" confounding domains are those for which, in the context of this study, adjustment is expected to lead to a clinically important change in the estimated effect of the intervention. "Validity" refers to whether the confounding variable or variables fully measure the domain, while "reliability" refers to the precision of the measurement (more measurement error means less reliability).

**(i) Confounding domains listed in the review protocol**				
Confounding domain	Measured variable(s)	Is there evidence that controlling for this variable was unnecessary?*	Is the confounding domain measured validly and reliably by this variable (or these variables)?	OPTIONAL: Is failure to adjust for this variable (alone) expected to favour the experimental intervention or the comparator?
Age	Not evaluated as confounder	No	-	-

**(ii) Additional confounding domains relevant to the setting of this particular study, or which the study authors identified as important**				
Confounding domain	Measured variable(s)	Is there evidence that controlling for this variable was unnecessary?*	Is the confounding domain measured validly and reliably by this variable (or these variables)?	OPTIONAL: Is failure to adjust for this variable (alone) expected to favour the experimental intervention or the comparator?
-	-	-	-	-

* In the context of a particular study, variables can be demonstrated not to be confounders and so not included in the analysis: (a) if they are not predictive of the outcome; (b) if they are not predictive of intervention; or (c) because adjustment makes no or minimal difference to the estimated effect of the primary parameter. Note that "no statistically significant association" is not the same as "not predictive".

Preliminary consideration of co-interventions

Complete a row for each important co-intervention (i) listed in the review protocol; and (ii) relevant to the setting of this particular study, or which the study authors identified as important.

"Important" co-interventions are those for which, in the context of this study, adjustment is expected to lead to a clinically important change in the estimated effect of the intervention.

**(i) Co-interventions listed in the review protocol**		
Co-intervention	Is there evidence that controlling for this co-intervention was unnecessary (e.g. because it was not administered)?	Is presence of this co-intervention likely to favour outcomes in the experimental intervention or the comparator
None	-	-

**(ii) Additional co-interventions relevant to the setting of this particular study, or which the study authors identified as important**		
Co-intervention	Is there evidence that controlling for this co-intervention was unnecessary (e.g. because it was not administered)?	Is presence of this co-intervention likely to favour outcomes in the experimental intervention or the comparator
None	-	-

Risk of bias assessment

Responses underlined in green are potential markers for low risk of bias, and responses in red are potential markers for a risk of bias. Where questions relate only to sign posts to other questions, no formatting is used.

Signalling questions	Description	Response options
**Bias due to confounding**		
1.1 Is there potential for confounding of the effect of intervention in this study?<br>**If N/PN to 1.1:** the study can be considered to be at low risk of bias due to confounding and no further signalling questions need be considered		Y / PY / PN / N
**If Y/PY to 1.1**: determine whether there is a need to assess time-varying confounding:		
1.2. Was the analysis based on splitting participants' follow up time according to intervention received?<br>**If N/PN**, answer questions relating to baseline confounding (1.4 to 1.6)<br>**If Y/PY**, go to question 1.3.		NA / Y / PY / PN / N / NI
1.3. Were intervention discontinuations or switches likely to be related to factors that are prognostic for the outcome?<br>**If N/PN**, answer questions relating to baseline confounding (1.4 to 1.6)<br>**If Y/PY**, answer questions relating to both baseline and time-varying confounding (1.7 and 1.8)		NA / Y / PY / PN / N / NI

**Questions relating to baseline confounding only**		
1.4. Did the authors use an appropriate analysis method that controlled for all the important confounding domains?		NA / Y / PY / PN / N / NI
1.5. **If Y/PY to 1.4**: Were confounding domains that were controlled for measured validly and reliably by the variables available in this study?		NA / Y / PY / PN / N / NI
1.6. Did the authors control for any post-intervention variables that could have been affected by the intervention?		NA / Y / PY / PN / N / NI
**Questions relating to baseline and time-varying confounding**		
1.7. Did the authors use an appropriate analysis method that controlled for all the important confounding domains and for time-varying confounding?		NA / Y / PY / PN / N / NI
1.8. **If Y/PY to 1.7**: Were confounding domains that were controlled for measured validly and reliably by the variables available in this study?		NA / Y / PY / PN / N / NI
**Risk of bias judgement**		Low / Moderate / Serious / Critical / NI
Optional: What is the predicted direction of bias due to confounding?		Favours experimental / Favours comparator / Unpredictable

Bias in selection of participants into the study		
2.1. Was selection of participants into the study (or into the analysis) based on participant characteristics observed after the start of intervention?<br>**If N/PN to 2.1:** go to 2.4		Y / PY / PN / N / NI
2.2. **If Y/PY to 2.1**: Were the post-intervention variables that influenced selection likely to be associated with intervention?		NA / Y / PY / PN / N / NI
2.3 **If Y/PY to 2.2**: Were the post-intervention variables that influenced selection likely to be influenced by the outcome or a cause of the outcome?		NA / Y / PY / PN / N / NI
2.4. Do start of follow-up and start of intervention coincide for most participants?		Y / PY / PN / N / NI
2.5. **If Y/PY to 2.2 and 2.3, or N/PN to 2.4**: Were adjustment techniques used that are likely to correct for the presence of selection biases?		NA / Y / PY / PN / N / NI
**Risk of bias judgement**		Low / Moderate / Serious / Critical / NI
Optional: What is the predicted direction of bias due to selection of participants into the study?		Favours experimental / Favours comparator / Towards null /Away from null / Unpredictable

Bias in classification of interventions		
3.1 Were intervention groups clearly defined?		Y / PY / PN / N / NI
3.2 Was the information used to define intervention groups recorded at the start of the intervention?		Y / PY / PN / N / NI
3.3 Could classification of intervention status have been affected by knowledge of the outcome or risk of the outcome?		Y / PY / PN / N / NI
**Risk of bias judgement**		Low / Moderate / Serious / Critical / NI
Optional: What is the predicted direction of bias due to classification of interventions?		Favours experimental / Favours comparator / Towards null /Away from null / Unpredictable

Bias due to deviations from intended interventions		
**If your aim for this study is to assess the effect of assignment to intervention, answer questions 4.1 and 4.2**		
4.1. Were there deviations from the intended intervention beyond what would be expected in usual practice?		Y / PY / PN / N / NI
4.2. **If Y/PY to 4.1**: Were these deviations from intended intervention unbalanced between groups *and* likely to have affected the outcome?		NA / Y / PY / PN / N / NI
**If your aim for this study is to assess the effect of starting and adhering to intervention, answer questions 4.3 to 4.6**		
4.3. Were important co-interventions balanced across intervention groups?		Y / PY / PN / N / NI
4.4. Was the intervention implemented successfully for most participants?		Y / PY / PN / N / NI
4.5. Did study participants adhere to the assigned intervention regimen?		Y / PY / PN / N / NI
4.6. **If N/PN to 4.3, 4.4 or 4.5**: Was an appropriate analysis used to estimate the effect of starting and adhering to the intervention?		NA / Y / PY / PN / N / NI
**Risk of bias judgement**		Low / Moderate / Serious / Critical / NI
Optional: What is the predicted direction of bias due to deviations from the intended interventions?		Favours experimental / Favours comparator / Towards null /Away from null / Unpredictable

Bias due to missing data		
5.1 Were outcome data available for all, or nearly all, participants?		Y / PY / PN / N / NI
5.2 Were participants excluded due to missing data on intervention status?		Y / PY / PN / N / NI
5.3 Were participants excluded due to missing data on other variables needed for the analysis?		Y / PY / PN / N / NI
5.4 **If PN/N to 5.1, or Y/PY to 5.2 or 5.3**: Are the proportion of participants and reasons for missing data similar across interventions?		NA / Y / PY / PN / N / NI
5.5 **If PN/N to 5.1, or Y/PY to 5.2 or 5.3**: Is there evidence that results were robust to the presence of missing data?		NA / Y / PY / PN / N / NI
**Risk of bias judgement**		Low / Moderate / Serious / Critical / NI
Optional: What is the predicted direction of bias due to missing data?		Favours experimental / Favours comparator / Towards null /Away from null / Unpredictable

Bias in measurement of outcomes		
6.1 Could the outcome measure have been influenced by knowledge of the intervention received?		Y / PY / PN / N / NI
6.2 Were outcome assessors aware of the intervention received by study participants?		Y / PY / PN / N / NI
6.3 Were the methods of outcome assessment comparable across intervention groups?		Y / PY / PN / N / NI
6.4 Were any systematic errors in measurement of the outcome related to intervention received?		Y / PY / PN / N / NI
**Risk of bias judgement**		Low / Moderate / Serious / Critical / NI
Optional: What is the predicted direction of bias due to measurement of outcomes?		Favours experimental / Favours comparator / Towards null /Away from null / Unpredictable

Bias in selection of the reported result		
Is the reported effect estimate likely to be selected, on the basis of the results, from...<br>7.1. ... multiple outcome *measurements* within the outcome domain?		Y / PY / PN / N / NI
7.2 ... multiple *analyses* of the intervention-outcome relationship?		Y / PY / PN / N / NI
7.3 ... different *subgroups*?		Y / PY / PN / N / NI
**Risk of bias judgement**		Low / Moderate / Serious / Critical / NI
Optional: What is the predicted direction of bias due to selection of the reported result?		Favours experimental / Favours comparator / Towards null /Away from null / Unpredictable

Overall bias		
**Risk of bias judgement**		Low / Moderate / Serious / Critical / NI
Optional: What is the overall predicted direction of bias for this outcome?		Favours experimental / Favours comparator / Towards null /Away from null / Unpredictable

This work is licensed under a Creative Commons Attribution-NonCommercial-NoDerivatives 4.0 International License.

**Domain**	**Recurrence**
Bias due to confounding	Serious risk
Bias in selection of participants into the study	Low risk
Bias in classification of interventions	Low risk
Bias due to deviations from intended interventions	Low risk
Bias due to missing data	Low risk
Bias in measurement of outcomes	Low risk
Bias in selection of the reported result	Moderate risk
**Overall**	**Serious risk**

Young 2007

Specify a target randomized trial specific to the study

Design	Individually randomized / Cluster randomized / Matched (e.g. cross-over)
Participants	Patients with histopathological diagnosis of AIS after cervical excision
Experimental intervention	Conservative treatment
Comparator	Demolitive treatment (hysterectomy)

Is your aim for this study…?

- ■ to assess the effect of *assignment to* intervention
- ☐ to assess the effect of *starting and adhering to* intervention

Specify the outcome

Specify which outcome is being assessed for risk of bias (typically from among those earmarked for the Summary of Findings table). Specify whether this is a proposed benefit or harm of intervention.

Recurrence<br>Residual

Specify the numerical result being assessed

In case of multiple alternative analyses being presented, specify the numeric result (e.g. RR = 1.52 (95% CI 0.83 to 2.77) and/or a reference (e.g. to a table, figure or paragraph) that uniquely defines the result being assessed.

Preliminary consideration of confounders

Complete a row for each important confounding domain (i) listed in the review protocol; and (ii) relevant to the setting of this particular study, or which the study authors identified as potentially important.

"Important" confounding domains are those for which, in the context of this study, adjustment is expected to lead to a clinically important change in the estimated effect of the intervention. "Validity" refers to whether the confounding variable or variables fully measure the domain, while "reliability" refers to the precision of the measurement (more measurement error means less reliability).

**(i) Confounding domains listed in the review protocol**				
Confounding domain	Measured variable(s)	Is there evidence that controlling for this variable was unnecessary?*	Is the confounding domain measured validly and reliably by this variable (or these variables)?	OPTIONAL: Is failure to adjust for this variable (alone) expected to favour the experimental intervention or the comparator?
Age	Not evaluated as confounder	No	-	-

**(ii) Additional confounding domains relevant to the setting of this particular study, or which the study authors identified as important**				
Confounding domain	Measured variable(s)	Is there evidence that controlling for this variable was unnecessary?*	Is the confounding domain measured validly and reliably by this variable (or these variables)?	OPTIONAL: Is failure to adjust for this variable (alone) expected to favour the experimental intervention or the comparator?
-	-	-	-	-

* In the context of a particular study, variables can be demonstrated not to be confounders and so not included in the analysis: (a) if they are not predictive of the outcome; (b) if they are not predictive of intervention; or (c) because adjustment makes no or minimal difference to the estimated effect of the primary parameter. Note that "no statistically significant association" is not the same as "not predictive".

Preliminary consideration of co-interventions

Complete a row for each important co-intervention (i) listed in the review protocol; and (ii) relevant to the setting of this particular study, or which the study authors identified as important.

"Important" co-interventions are those for which, in the context of this study, adjustment is expected to lead to a clinically important change in the estimated effect of the intervention.

**(i) Co-interventions listed in the review protocol**		
Co-intervention	Is there evidence that controlling for this co-intervention was unnecessary (e.g. because it was not administered)?	Is presence of this co-intervention likely to favour outcomes in the experimental intervention or the comparator
None	-	-

**(ii) Additional co-interventions relevant to the setting of this particular study, or which the study authors identified as important**		
Co-intervention	Is there evidence that controlling for this co-intervention was unnecessary (e.g. because it was not administered)?	Is presence of this co-intervention likely to favour outcomes in the experimental intervention or the comparator
None	-	-

Risk of bias assessment

Responses underlined in green are potential markers for low risk of bias, and responses in red are potential markers for a risk of bias. Where questions relate only to sign posts to other questions, no formatting is used.

Signalling questions	Description	Response options
**Bias due to confounding**		
1.1 Is there potential for confounding of the effect of intervention in this study?<br>**If N/PN to 1.1:** the study can be considered to be at low risk of bias due to confounding and no further signalling questions need be considered		Y / PY / PN / N
**If Y/PY to 1.1**: determine whether there is a need to assess time-varying confounding:		
1.2. Was the analysis based on splitting participants' follow up time according to intervention received?<br>**If N/PN**, answer questions relating to baseline confounding (1.4 to 1.6)<br>**If Y/PY**, go to question 1.3.		NA / Y / PY / PN / N / NI
1.3. Were intervention discontinuations or switches likely to be related to factors that are prognostic for the outcome?<br>**If N/PN**, answer questions relating to baseline confounding (1.4 to 1.6)<br>**If Y/PY**, answer questions relating to both baseline and time-varying confounding (1.7 and 1.8)		NA / Y / PY / PN / N / NI

**Questions relating to baseline confounding only**		
1.4. Did the authors use an appropriate analysis method that controlled for all the important confounding domains?		NA / Y / PY / PN / N / NI
1.5. **If Y/PY to 1.4**: Were confounding domains that were controlled for measured validly and reliably by the variables available in this study?		NA / Y / PY / PN / N / NI
1.6. Did the authors control for any post-intervention variables that could have been affected by the intervention?		NA / Y / PY / PN / N / NI
**Questions relating to baseline and time-varying confounding**		
1.7. Did the authors use an appropriate analysis method that controlled for all the important confounding domains and for time-varying confounding?		NA / Y / PY / PN / N / NI
1.8. **If Y/PY to 1.7**: Were confounding domains that were controlled for measured validly and reliably by the variables available in this study?		NA / Y / PY / PN / N / NI
**Risk of bias judgement**		Low / Moderate / Serious / Critical / NI
Optional: What is the predicted direction of bias due to confounding?		Favours experimental / Favours comparator / Unpredictable

Bias in selection of participants into the study		
2.1. Was selection of participants into the study (or into the analysis) based on participant characteristics observed after the start of intervention?<br>**If N/PN to 2.1:** go to 2.4		Y / PY / PN / N / NI
2.2. **If Y/PY to 2.1**: Were the post-intervention variables that influenced selection likely to be associated with intervention?		NA / Y / PY / PN / N / NI
2.3 **If Y/PY to 2.2**: Were the post-intervention variables that influenced selection likely to be influenced by the outcome or a cause of the outcome?		NA / Y / PY / PN / N / NI
2.4. Do start of follow-up and start of intervention coincide for most participants?		Y / PY / PN / N / NI
2.5. **If Y/PY to 2.2 and 2.3, or N/PN to 2.4**: Were adjustment techniques used that are likely to correct for the presence of selection biases?		NA / Y / PY / PN / N / NI
**Risk of bias judgement**		Low / Moderate / Serious / Critical / NI
Optional: What is the predicted direction of bias due to selection of participants into the study?		Favours experimental / Favours comparator / Towards null /Away from null / Unpredictable

Bias in classification of interventions		
3.1 Were intervention groups clearly defined?		Y / PY / PN / N / NI
3.2 Was the information used to define intervention groups recorded at the start of the intervention?		Y / PY / PN / N / NI
3.3 Could classification of intervention status have been affected by knowledge of the outcome or risk of the outcome?		Y / PY / PN / N / NI
**Risk of bias judgement**		Low / Moderate / Serious / Critical / NI
Optional: What is the predicted direction of bias due to classification of interventions?		Favours experimental / Favours comparator / Towards null /Away from null / Unpredictable

Bias due to deviations from intended interventions		
**If your aim for this study is to assess the effect of assignment to intervention, answer questions 4.1 and 4.2**		
4.1. Were there deviations from the intended intervention beyond what would be expected in usual practice?		Y / PY / PN / N / NI
4.2. **If Y/PY to 4.1**: Were these deviations from intended intervention unbalanced between groups *and* likely to have affected the outcome?		NA / Y / PY / PN / N / NI
**If your aim for this study is to assess the effect of starting and adhering to intervention, answer questions 4.3 to 4.6**		
4.3. Were important co-interventions balanced across intervention groups?		Y / PY / PN / N / NI
4.4. Was the intervention implemented successfully for most participants?		Y / PY / PN / N / NI
4.5. Did study participants adhere to the assigned intervention regimen?		Y / PY / PN / N / NI
4.6. **If N/PN to 4.3, 4.4 or 4.5**: Was an appropriate analysis used to estimate the effect of starting and adhering to the intervention?		NA / Y / PY / PN / N / NI
**Risk of bias judgement**		Low / Moderate / Serious / Critical / NI
Optional: What is the predicted direction of bias due to deviations from the intended interventions?		Favours experimental / Favours comparator / Towards null /Away from null / Unpredictable

Bias due to missing data		
5.1 Were outcome data available for all, or nearly all, participants?		Y / PY / PN / N / NI
5.2 Were participants excluded due to missing data on intervention status?		Y / PY / PN / N / NI
5.3 Were participants excluded due to missing data on other variables needed for the analysis?		Y / PY / PN / N / NI
5.4 **If PN/N to 5.1, or Y/PY to 5.2 or 5.3**: Are the proportion of participants and reasons for missing data similar across interventions?		NA / Y / PY / PN / N / NI
5.5 **If PN/N to 5.1, or Y/PY to 5.2 or 5.3**: Is there evidence that results were robust to the presence of missing data?		NA / Y / PY / PN / N / NI
**Risk of bias judgement**		Low / Moderate / Serious / Critical / NI
Optional: What is the predicted direction of bias due to missing data?		Favours experimental / Favours comparator / Towards null /Away from null / Unpredictable

Bias in measurement of outcomes		
6.1 Could the outcome measure have been influenced by knowledge of the intervention received?		Y / PY / PN / N / NI
6.2 Were outcome assessors aware of the intervention received by study participants?		Y / PY / PN / N / NI
6.3 Were the methods of outcome assessment comparable across intervention groups?		Y / PY / PN / N / NI
6.4 Were any systematic errors in measurement of the outcome related to intervention received?		Y / PY / PN / N / NI
**Risk of bias judgement**		Low / Moderate / Serious / Critical / NI
Optional: What is the predicted direction of bias due to measurement of outcomes?		Favours experimental / Favours comparator / Towards null /Away from null / Unpredictable

Bias in selection of the reported result		
Is the reported effect estimate likely to be selected, on the basis of the results, from...		
7.1. ... multiple outcome *measurements* within the outcome domain?		Y / PY / PN / N / NI
7.2 ... multiple *analyses* of the intervention-outcome relationship?		Y / PY / PN / N / NI
7.3 ... different *subgroups*?		Y / PY / PN / N / NI
**Risk of bias judgement**		Low / Moderate / Serious / Critical / NI
Optional: What is the predicted direction of bias due to selection of the reported result?		Favours experimental / Favours comparator / Towards null /Away from null / Unpredictable

Overall bias		
**Risk of bias judgement**		Low / Moderate / Serious / Critical / NI
Optional: What is the overall predicted direction of bias for this outcome?		Favours experimental / Favours comparator / Towards null /Away from null / Unpredictable

This work is licensed under a Creative Commons Attribution-NonCommercial-NoDerivatives 4.0 International License.

**Domain**	**Recurrence**	**Residual**
Bias due to confounding	Serious risk	Serious risk
Bias in selection of participants into the study	Low risk	Low risk
Bias in classification of interventions	Low risk	Low risk
Bias due to deviations from intended interventions	Low risk	Low risk
Bias due to missing data	Low risk	Low risk
Bias in measurement of outcomes	Low risk	Low risk
Bias in selection of the reported result	Moderate risk	Moderate risk
**Overall**	**Serious risk**	**Serious risk**
