# Supplementary material for: Clinical Outcomes of Cervical Adenocarcinoma In Situ According to Conservative or Demolitive Treatment: A Systematic Review and Meta-Analysis
Source: Cancers (Basel). 2025 May 30;17(11):1839. doi: 10.3390/cancers17111839 (PMC12153624; doi:10.3390/cancers17111839)
Supplement: Supplementary file 1 [file cancers-17-01839-s001.zip › Revision Supplementary File S4.pdf]

Supplementary File S4 - Patients characteristics of the included studies

First author Year	Age (global)	Age (conservative)	Age (demolitive)	Parity (global)	Parity (conservative)	Parity (demolitive)	Menopause	Oral contraceptive use	Smoking
Azodi 1999 [24]	Mean 37 range (19-71)	NA	NA	Mean 1.3 range (0-6)	NA	NA	NA	32%	40%
Baalbergen 2014 [25]	Mean 37 range (31-42)	NA	NA	35% nulliparous	NA	NA	NA	NA	NA
Bryson 2004 [26]	Mean 37 range (24-44)	NA	NA	NA	NA	NA	NA	NA	NA
Dalrymple 2008 [27]	Mean 36 range (22-65)	NA	NA	NA	NA	NA	NA	NA	NA
Dostalek 2023 [28]	Mean 38.7	NA	NA	NA	NA	NA	NA	NA	NA
Giannella 2022 [29]	Mean 40.5 range (34-49)	NA	NA	44.6% nulliparous	NA	NA	17.6%	NA	25.6%
Hwang 2004 [30]	Mean 35.5 range (24-67)	Mean 33.3 range (24-62)	Mean 40.1 range (26-67)	49%	67%	12%	NA	54.2%	23%
Im 1995 [31]	Mean 35 range (26-46)	NA	NA	21%	NA	NA	NA	NA	25%
Li 2013 [32]	Mean 35 range (18-75)	Mean 31 range (18-75)	Mean 40 range (26-63)	NA	NA	NA	NA	NA	NA
Liu 2022 [33]	Mean ± SD 41.5 ± 8.7	Mean ± SD 34.5 ± 5.6	Mean ± SD 41.5 ± 8.7	NA	NA	NA	NA	NA	NA
Omnes 2003 [34]	Mean 40 range (28-51)	Mean 37.6 range (28-50)	Mean 46 range (41-51)	Mean 1.53 range (0-6)	NA	NA	NA	NA	NA

Schaafsma 2025 [35]	Mean 36.3 range (30-50)	Mean 34.6 range (30-40)	Mean 43 range (39-50)	NA	NA	NA	NA	NA	NA
Shin 2000 [36]	Mean 32.1 range (17-72)	Mean 29 range (17-47)	Mean 40 range (25-72)	75.8% nulliparous	77% nulliparous	72.9% nulliparous	NA	NA	NA
Song 2015 [37]	Mean 42 range (25-67)	NA	NA	2 (0-5)	NA	NA	14.1%	NA	NA
Tay 1999 [38]	Mean 44.2 range (32-68)	NA	NA	4.1% nulliparous	NA	NA	NA	NA	NA
Taylor 2014 [39]	Mean 33 range (17-51)	NA	NA	NA	NA	NA	NA	NA	NA
Wang 2020 [40]	Mean 37.7 range (24-71)	NA	NA	NA	NA	NA	13.4%	NA	NA
Wolf 1997 [41]	Mean 35.9 range (24-64)	NA	NA	Median 2 range (0-9)	NA	NA	NA	23%	20%
Young 2007 [42]	Mean 34.3 range (18-73)	Mean 30.4	Mean 40.3	19% nulliparous	NA	NA	NA	39.3%	34%

NA: Not Available
