# Supplementary material for: Clinical Outcomes of Cervical Adenocarcinoma In Situ According to Conservative or Demolitive Treatment: A Systematic Review and Meta-Analysis
Source: Cancers (Basel). 2025 May 30;17(11):1839. doi: 10.3390/cancers17111839 (PMC12153624; doi:10.3390/cancers17111839)
Supplement: Supplementary file 1 [file cancers-17-01839-s001.zip › Revision Supplementary File S5.pdf]

Supplementary File S5 - Clinical characteristics of the included studies

First author Year	HPV positivity	Abnormal cytology	Colposcopic impression	Histology at biopsy	Conization technique	Cone length	Co-existence with squamous disease	ECC performed	ECC abnormal	Margin positivity	Second conization	Pregnancy rate	Follow-up modality	Follow-up duration (months)
Azodi 1999 [24]	NA	55% glandular 25% squamous 18% mixed	NA	50% AIS 5% squamous dysplasia 20% mixed	60.0% CKC 20% LEEP 20% Laser	NA	28.0%	67.5%	41.0%	57.5%	12.0%	NA	NA	38
Baalbergen 2014 [25]	95.8%	60% AGC-FN or adenocarcinoma 28% AGC-NOS	39% CIN 33% glandular pathology 11% normal 9% not mentioned 7% no colposcopy	NA	55.6% CKC 41.9% LEEP 2.5% Laser	NA	43.0%	NA	NA	27.3%	30.2%	28.0%	57% cytology alone 2% cytology + ECC 24% cytology + HR-HPV 3% cytology + ECC + HR-HPV 14% no follow-up	46
Bryson 2004 [26]	NA	NA	no identification of glandular abnormalities	29.0% EGD 71.0% ACIS	100% LEEP	NA	NA	NA	NA	32.3%	9.7%	NA	Brush cytology, colposcopy, ECC	40
Dalrymple 2008 [27]	NA	46.9% glandular abnormalities 28.4% associated squamous lesion	NA	NA	53.7% Laser 46.3% CKC	NA	34.1%	NA	NA	17.1%	1.2%	10.8%	NA	5.6

		24.7% squamous												
Dostalek 2023 [28]	NA	NA	NA	NA	76% Fisher Cone Biopsy Excisor, 21% Electrofrequency 2% CKC 1% simple vaginal trachelectomy	NA	66.1%	NA	NA	22.3%	22.3%	NA	HPV test, cytology	56.6
Giannella 2022 [29]	83.8%	12.2% ASCUS/LSIL 63.5% ASCH+ 12.8% AGC-NOS 2.7% AGC-FN 5.4% AIS	NA	NA	7.4% CKC 24.3% Laser 68.3% LEEP	Median 16.5 mm	NA	NA	NA	28.8%	28.8%	NA	HPV test, cytology	60
Hwang 2004 [30]	NA	54% abnormal glandular cells/ACIS 64% SIL 17% both SIL and abnormal glandular cells	NA	76.4% glandular dysplasia or adenocarcinoma 18.1% SIL	23.8% CKC 48.8% Laser 27.4% LEEP	NA	NA	NA	NA	34.7%	16.4%	NA	NA	60
Im 1995	NA	NA	NA	NA	88.8% CKC	NA	50.0%	NA	NA	33.0%	5.6%	NA	frequent pelvic examination,	20.4

[31]					5.6%<br>Laser<br>5.6%<br>LEEP								cervical<br>cytology	
Li<br>2013<br>[32]	95.3%	11.8%<br>ASCUS<br>8.1%<br>LSIL<br>12.5%<br>HSIL<br>39.7%<br>AIS/AGC<br>10.3%<br>AGC + SIL<br>3.7%<br>AGC + ASCH	NA	NA	Conization,<br>not<br>specified	NA	41.2%	NA	NA	38.2%	31.0%	NA	cytology /<br>histology /<br>HPV test	42
Liu<br>2022<br>[33]	97.9%	67.4%<br>abnormal	60.9%<br>transformation<br>zone type III<br>39.1%<br>type I-II	NA	66.2%<br>CKC<br>33.8%<br>LEEP	NA	68.0%	NA	NA	19.7%	NA	NA	cytology and<br>HPV testing<br>every 3 months<br>within 2 years,<br>every 6 months<br>for 3–5 years,<br>and once a year<br>thereafter	60
Omnes<br>2003<br>[34]	NA	81.3%<br>abnormal	37.5%<br>grade 2<br>abnormality	43.8%<br>AIS<br>31.3%<br>CIN2-3	30%<br>LEEP<br>50%<br>CKC<br>20%<br>trachelecto<br>my	33.3%<br>8 mm<br>33.3%<br>10-25<br>mm<br>33.3%<br>25-38<br>mm	NA	NA	NA	12.5%	12.5%	NA	NA	73.2
Schaafsma<br>2025<br>[35]	NA	NA	NA	NA	42.9%<br>LLETZ<br>57.1%<br>CKC	NA	64.6%	NA	NA	33.4%	23.3%	NA	cytology, HPV<br>DNA	117.6

Shin 2000 [36]	NA	NA	NA	NA	72.7% CKC 27.3% LEEP	NA	42.0%	NA	NA	9.2%	22.0%	21.6%	Cervico-vaginal smears, colposcopy, ectocervical biopsies, and endocervical curettage performed at differing intervals	30
Song 2015 [37]	66.2%	69.0% squamous abnormality 19.7% glandular abnormality 5.6% combined	NA	42.3% squamous lesion 32.4% glandular lesion 7.0% combined	100% LEEP	Mean 23 mm	57.7%	28.2%	25.0%	28.6%	5.6%	NA	NA	57.1
Tay 1999 [38]	NA	90% Abnormal 39% AGC	81% abnormal 29% glandular abnormality	93% abnormal 57% glandular abnormali ty	80% laser, 10% CKC 10% LEETZ	NA	66.6%	16.7%	75.0%	29.2%	4.1%	NA	routine examination, symptom review, pap smear, one patient ECC	20
Taylor 2014 [39]	NA	NA	NA	NA	28.8% LEEP 71.2% CKC	NA	NA	NA	NA	50.0%	23.0%	NA	cytology and endocervical curettage	31.2
Wang 2020 [40]	NA	NA	NA	NA	79.8% CKC 20.2% ESC	NA	66.7%	NA	NA	14.9%	NA	28.3%	NA	36.5
Wolf 1997 [41]	NA	50% glandular abnormality 39%	94.7% abnormal	35% abnormali ty of the glandular epithelium	85% CKC 13% LEEP 2% laser	NA	35.0%	70.5%	NA	46.0%	NA	NA	NA	57

		squamous atypia or dysplasia												
Young 2007 [42]	74%	34% AIS 19% AGC 30% HSIL 4% LSIL 5.4% ASC-US	NA	NA	72.2% CKC 12.5% LEEP 2.8% Laser 12.5% Fisher Excisor	Mean 14.2 mm	45.9%	57.0%	11.0%	30.0%	39.0%	27.5%	NA	25.2

NA: Not Available
