# Supplementary material for: Clinical Outcomes of Cervical Adenocarcinoma In Situ According to Conservative or Demolitive Treatment: A Systematic Review and Meta-Analysis
Source: Cancers (Basel). 2025 May 30;17(11):1839. doi: 10.3390/cancers17111839 (PMC12153624; doi:10.3390/cancers17111839)

Supplementary File S6 - Funnel plots for recurrence during follow-up and residual after positive margin

AIS recurrence

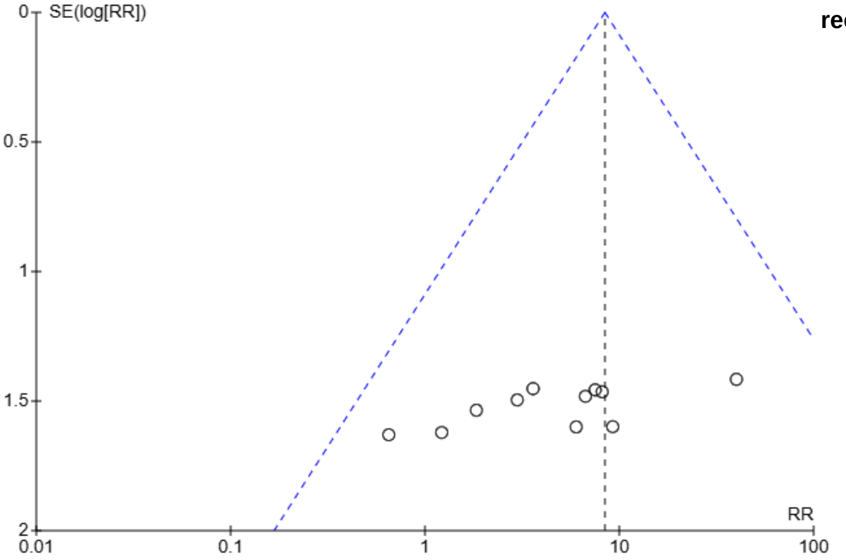

Invasive recurrence

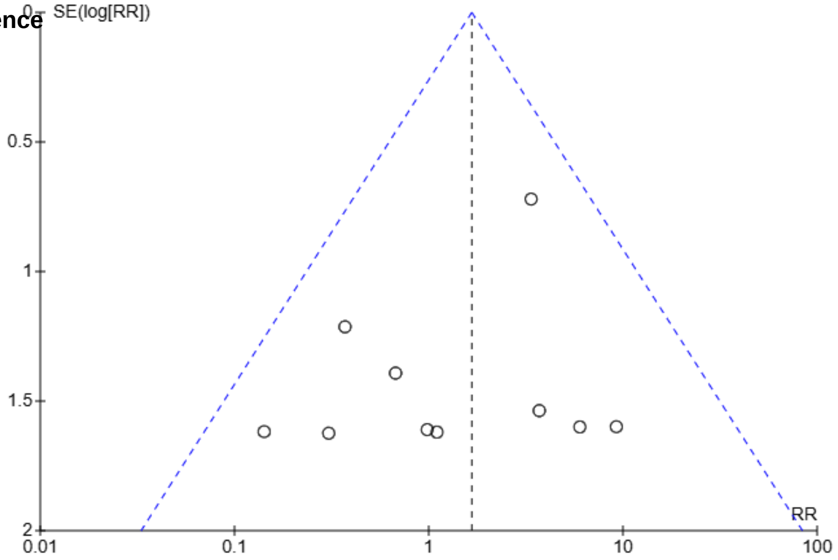

AIS residual

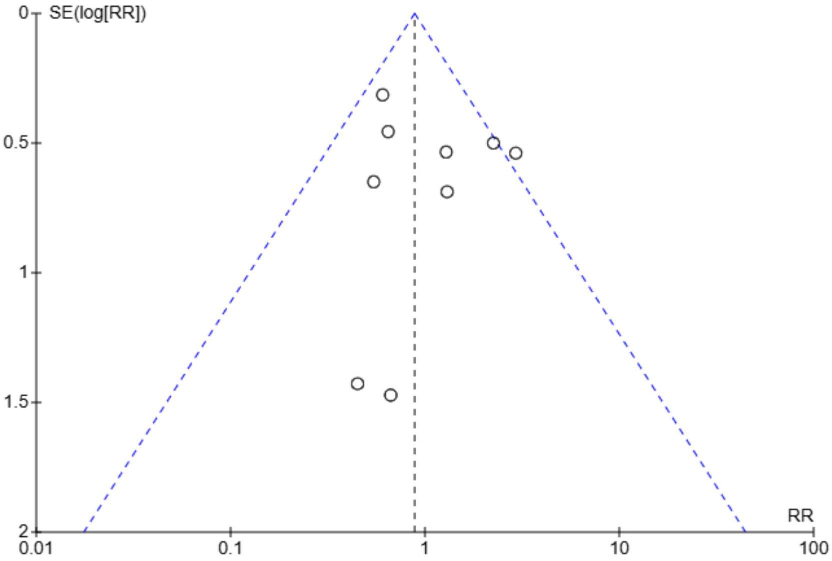

Invasive residual

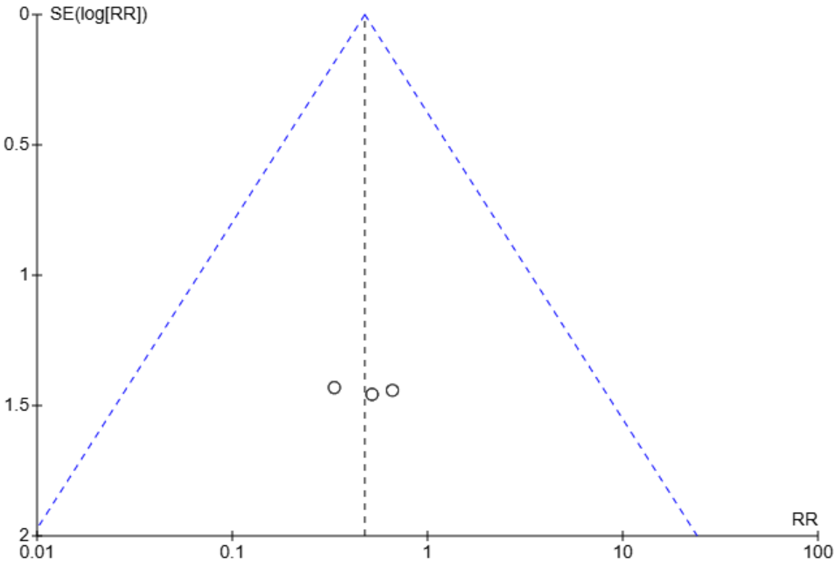

Supplement: Supplementary file 1 [file cancers-17-01839-s001.zip › Revision Supplementary File S6.pdf]
